# Smad1 Promotes Tumorigenicity and Chemoresistance of Glioblastoma by Sequestering p300 From p53

**DOI:** 10.1002/advs.202402258

**Published:** 2024-12-04

**Authors:** Lingli Gong, Daxing Xu, Kaixiang Ni, Jie Li, Wei Mao, Bo Zhang, Zhening Pu, Xiangming Fang, Ying Yin, Li Ji, Jingjing Wang, Yaling Hu, Jiao Meng, Rui Zhang, Jiantong Jiao, Jian Zou

**Affiliations:** ^1^ Department of Laboratory Medicine The Affiliated Wuxi People's Hospital of Nanjing Medical University, Wuxi People's Hospital Wuxi Medical Center Nanjing Medical University Wuxi Jiangsu 214023 China; ^2^ Wuxi Medical Center Nanjing Medical University Wuxi Jiangsu 214023 China; ^3^ Department of Neurosurgery The Affiliated Wuxi People's Hospital of Nanjing Medical University Wuxi Jiangsu 214023 China; ^4^ Center of Clinical Research The Affiliated Wuxi People's Hospital of Nanjing Medical University Wuxi Jiangsu 214023 China; ^5^ Department of Radiology The Affiliated Wuxi People's Hospital of Nanjing Medical University Wuxi Jiangsu 214023 China

**Keywords:** glioblastoma, p300, p53, Smad1

## Abstract

Acetylation is critically required for p53 activation, though it remains poorly understood how p53 acetylation is regulated in glioblastoma (GBM). This study reveals that p53 acetylation is a favorable prognostic marker for GBM, regardless of p53 status, and that Smad1, a key negative regulator of p53 acetylation, is involved in this process. Smad1 forms a complex with p53 and p300, inhibiting p300's interaction with p53 and leading to reduced p53 acetylation and increased Smad1 acetylation in GBM. This results in enhanced tumor growth and resistance to chemotherapy, particularly in tumors with missense mutant p53. Acetylation of K373 is found to be essential for Smad1's oncogenic function but does not confer chemoresistance in the absence of p53. Through molecular docking, it is discovered that Smad1 and p53 both interact with the acetyltransferase domain of p300, but at different amino acid sites. Disturbing the interface of Smad1 through amino acid mutations abolishes the Smad1‐p300 complex and promotes p53 acetylation. Therefore, a small molecule is identified through virtual screening that specifically disrupts the Smad1‐p300 interaction, offering a promising strategy for inhibiting GBM and increasing chemosensitivity by inhibiting Smad1 acetylation and restoring p53 acetylation.

## Graphical abstract

Smad1 forms a complex with p53 and p300, leading to reduced p53 acetylation and increased Smad1 acetylation, promoting tumor growth and chemoresistance in tumors with mutant p53. Disrupting the Smad1‐p300 interaction with a small molecule, cpd.618, inhibits GBM and increases chemosensitivity by restoring p53 acetylation.

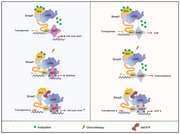

## 1 Introduction

Glioblastomas (GBMs) are the most frequent malignant primary human brain tumors, highly malignant, and difficult to completely cure, leading to poor outcomes and low overall survival due to their resistance to anticancer drugs and diverse disease phenotypes.^[^ ^1^ ^]^ Therefore, it is urgently needed to identify effective diagnostic, prognostic, and treatment indicators of GBM.

The p53 tumor suppressor is one of the most widely studied biomarkers and functional molecules in human cancers.^[^ ^2^, ^3^ ^]^ It is a central node of the cellular stress‐response pathway and crucial for cancer initiation, development, and malignant processes. Functional loss or transformation due to p53 gene/protein alterations is one of the most common molecular events in cancers.^[^ ^2^ ^]^ In human primary GBMs, ≈30% of samples harbor *TP53* mutations.^[^ ^4^ ^]^ *TP53* mutations are predominantly point mutations leading to amino acid substitutions in the DNA binding domain.^[^ ^5^ ^]^ The consensus view is that p53 mutants gain *de novo* functions and that this gain‐of‐function (GOF) contributes to malignant phenotypes in cancers.^[^ ^2^ ^]^ Additionally, p53 is frequently increased and associated with pathological grade glioma and poor overall survival.^[^ ^6^ ^]^ However, p53 mutations do not affect disease free survival or overall survival of GBM patients,^[^ ^7^ ^]^ suggesting that GOF may not fully explain the roles of mutated p53 in GBM.

Aberrant post‐translational modifications are a key molecular event that triggers the loss of p53 tumor suppressive function in tumors.^[^ ^8^ ^]^ p53 is the first identified non‐histone protein with acetylation modification capacity,^[^ ^9^ ^]^ and acetylation is a critical requisite for p53 activation.^[^ ^10^ ^]^ In general, p53 acetylation is carried out by histone acetyltransferase (HATs), including p300/CBP/PCAF or Tip60/MOF/MOZ on multiple lysine residues. Strikingly, mutant p53 can be acetylated at the same residues as wild‐type p53 with different outcomes (codons for those acetylated residues).^[^ ^11^, ^12^ ^]^ Evidence supports that mutant p53 retains the ability of wild‐type p53 after undergoing acetylation at the lysine residues,^[^ ^13^, ^14^, ^15^, ^16^, ^17^ ^]^ shedding light on the regulation of p53 acetylation as a therapeutic target for tumors containing mutant p53. While p53 is not the only substrate acetylated by HATs, its acetylation is influenced by different factors, such as changes in protein structure, protein‐protein interactions, and various physiological or pathological conditions.^[^ ^18^ ^]^ Therefore, it remains poorly understood whether p53 acetylation is associated with prognosis and how p53 acetylation is regulated in GBM.

In this study, we report that p53 acetylation is a favorable prognostic marker for GBM, regardless of p53 status, and is regulated by Smad1, an intracellular effector of bone morphogenetic proteins (BMPs) signaling, in a p300‐dependent manner. By forming a ternary complex with p53 and p300, Smad1 sequesters p300 from p53, leading to sustained hypoacetylation of p53 and hyperacetylation of Smad1, and promoting tumor growth and chemoresistance. A small molecule that directly and exclusively disrupts the Smad1‐p300 complex shows promise in inhibiting GBM and increasing chemosensitivity.

## 2 Result

### 2.1 Smad1 Upregulation is Associated with Tumorigenesis and Poor Prognosis of GBM

**Figure 1:**
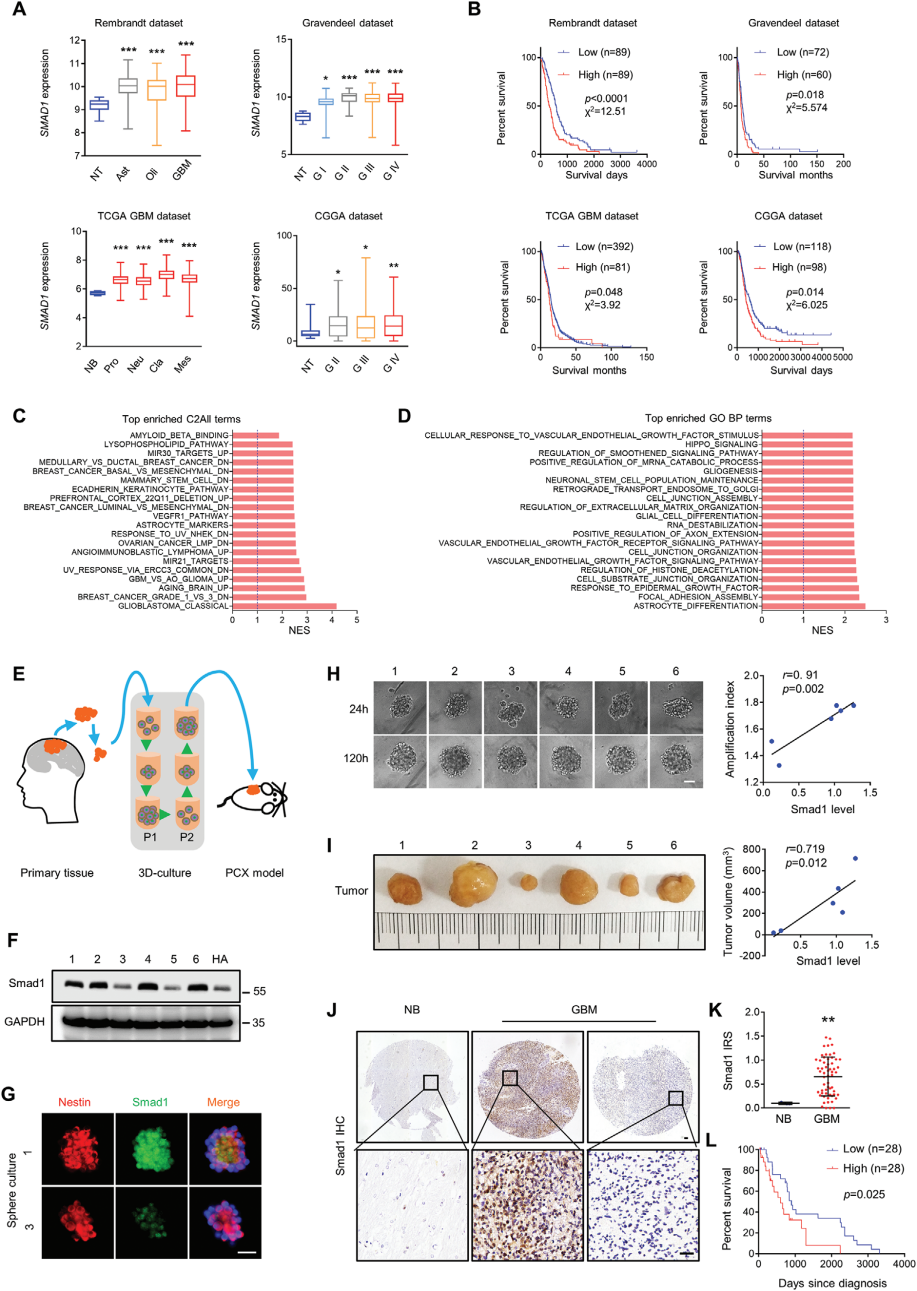
Smad1 is overexpressed in GBM, and its expression is associated with tumorigenesis. A) Indicated glioma datasets showed *SMAD1* is overexpressed in different grades of gliomas and GBM subtypes. B) Overall survival analysis based on *SMAD1* expression in indicated GBM datasets (Log‐rank, *χ^2^* and *p* values indicated). C) Top enriched C2ALL gene categories for the TCGA GBM dataset derived from Gliovis processed by GenePattern based on genes positively correlated with *SMAD1* expression. NES value of 1.0 and NOM *p* < 0.01 were used as visual thresholds. D) Top 10 enriched GO terms (C5: biological process) for the TCGA GBM dataset derived from Gliovis processed by GenePattern. E) Illustration of 3D‐PCX model of patient derived GBM tissues. The patient‐derived GBM cells were cultured in 3D and the second or third passages were used for xenograft models and subsequent analysis. F) Smad1 expression in the second passage of patient‐derived GBM cells was examined by Western blotting. GAPDH served as a loading control. G) Double immunofluorescence (IF) of Smad1 and Nestin in 3D cultured GBM primary cells. Hoechst (blue) stained nuclei. Scale bars, 100 µm. H) Smad1 expression was correlated with the growth of primary GBM cells. 3D‐tumor spheroids growth was recorded (left) and quantitatively analyzed (Spearman correlation analysis, *p* and *r* values indicated). The spheroid growth was reflected in the amplification index determined by calculating the area of sphere relative to 24 h. Scale bar, 100 µm. I) Xenograft tumors derived from 3D cultured patient‐derived GBM cells (left) and the correlation analysis of Smad1 and tumor volume (right; Spearman correlation analysis, *p* and *r* values indicated). J) Immunohistochemistry (IHC) staining for Smad1 in patient‐derived normal brain (NB) and glioblastoma (GBM) tissues. Scale bars, 50 µm. K) Smad1 was frequently increased in GBM as compared to normal brains (^*^ *p* < 0.05, Unpaired t‐test). Smad1 expression level was scored based on an immunoreactivity scoring (IRS) system. L) Overall survival analysis on Smad1 levels in GBM patients (Log‐rank *χ* ^2^ = 5.019, *p* = 0.025). Groups were ranked according to Smad1 median of IRS in GBM tissues.

An overview of TCGA RNA‐seq data derived from the Human Protein Atlas revealed that gliomas had the highest *SMAD1* transcriptional expression among cancers (Figure S1A, Supporting Information). A significant alteration of *SMAD1* was identified across all tumor samples and paired with normal tissues (Figure S1B, Supporting Information). Low‐grade glioma and GBM both had higher *SMAD1* expression. Among cancers with significant *SMAD1* alterations, GBMs had the highest fold change (Figure S1C, Supporting Information). Samples with significant *SMAD1* transcriptional expression were further characterized into different glioma datasets. It was revealed that *SMAD1* was highly expressed in gliomas, covering different histology, grades, and subtypes (**Figure** **1**A). Moreover, survival analysis indicated that higher *SMAD1* predicted poor GBM patient survival outcomes (Figure 1B). To further explore whether SMAD1 was a valuable predictor for GBM prognosis, Gene Set Enrichment Analysis (GSEA) was performed based on genes correlated with *SMAD1* expression derived from the TCGA GBM dataset. According to the C2All gene sets collected, the genes positively correlated with *SMAD1* were enriched in GBM‐related phenotypes (Figure 1C). From the perspective of biological processes (GoBP), the genes positively correlated with *SMAD1* were associated with astrocyte and glial cell differentiation, gliogenesis, and neuronal stem cell population maintenance (Figure 1D). To determine the significance of *SMAD1* in human GBM, we cultured primary cells from patient‐derived GBM tissue resections using the 3D‐culture method (Figure 1E). The second passage of 3D‐cultures was used for xenograft models and analysis. Western blot analysis detected the expression of Smad1 in patient‐derived 3D‐cultures (Figure 1F). Immunofluorescence (IF) illustrated that these patient derived spheres were Nestin positive and observed variable Smad1 expression (Figure 1G). The second passage of spheres was dissociated and cultured according to the 3D method, with continuous observation of sphere growth (Figure 1H). The tumor sphere growth was expressed as an amplification index by calculating the area of the sphere at 120 h relative to the area of the sphere at 24 h. Correlation analysis indicated that Smad1 protein levels were positively correlated with the amplification of the patient‐derived tumor spheres. To examine the correlation between Smad1 and GBM tumorigenesis, the third passage of 3D‐cultures was implanted subcutaneously in nude mice. The xenografts derived from cultures with higher Smad1 expression grew more quickly than those derived from cultures with lower Smad1. As shown in Figure 1I, the expression level of Smad1 in these cells was positively correlated with tumor volume derived from the 3D‐cultures, indicating that higher Smad1 corresponds to stronger tumorigenicity. To examine the general alteration of Smad1 protein in GBM, immunohistochemistry (IHC) was performed using a GBM tissue microarray containing 60 tumor samples and 4 normal samples (Figure 1J). As shown in the representative images, Smad1 was mostly expressed in the nuclei of normal brain and GBM tissues. This indicated that Smad1 protein levels were significantly higher in GBM than in normal brains (Figure 1K). Survival analysis based on the cutoff at a median level of Smad1 in GBM indicated that higher Smad1 was correlated with poorer clinical prognosis (Figure 1L, Log‐rank *χ* ^2^ = 5.019, *p* = 0.025) regardless of age or sex (Table S1 and Supplemental Data 1, Supporting Information). Collectively, these data suggest that Smad1 may be regarded as a valuable prognostic factor for GBM.

### 2.2 Smad1 Upregulation Promotes GBM Tumorigenesis and Chemoresistance In Vitro and In Vivo

**Figure 2:**
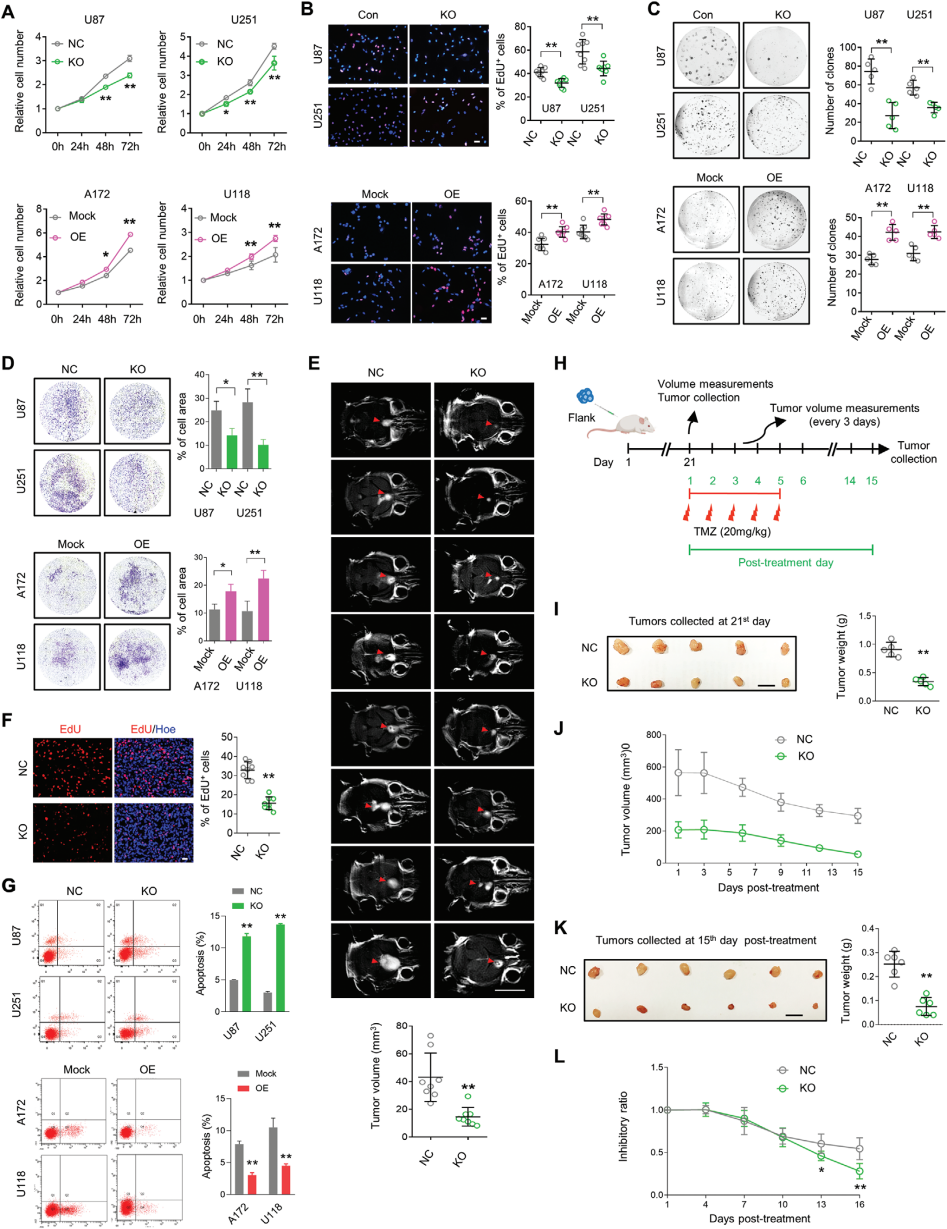
Smad1 promotes GBM tumorigenesis and chemoresistance in vitro and in vivo. A) Cell growth assay of cells with CRISPR/Cas9‐mediated Smad1 depletion (KO, upper) or with ectopic Smad1 expression (OE, lower; *n* = 3, ^*^ *p* < 0.05, ^**^ *p* < 0.01). B) EdU‐labeling assay detecting the DNA replication of cells with Smad1 KO (upper) or OE (lower; *n* = 8, ^**^ *p* < 0.01). Data were expressed as the percentage of EdU positive cells (Red) to total cells indicated by Hoechst labeling (Blue). Bars, 20 µM. C) Colony formation assay of cells with Smad1 KO (upper) or OE (lower; *n* = 5, ^**^ *p* < 0.01). D) Transwell invasion assay of cells with Smad1 KO or OE (lower; *n* = 5, ^**^ *p* < 0.01). E) Representative MRI images of brain tumor xenograft and tumor size quantification results derived from MRI images (*n* = 8, ^**^ *p* < 0.01). Scale bars, 1 mm. F) Images of EdU staining in growing tumors and the comparison of EdU‐positive rates (*n* = 8, ^**^ *p* < 0.01). Bars, 20 µm. G) Apoptosis assay of indicated GBM cells. Cells were treated with Doxorubicin (Dox, 2 µM) for 24 h and apoptotic cells were analyzed by FACS (*n* = 3, ^**^ *p* < 0.01). H) Schematic illustration of the evaluation of in vivo tumor growth derived from Smad1 depleted U87 cells or negative control cells to Temozolomide (TMZ) treatment. Mice were subcutaneously injected with U87 cells containing Smad1 depletion or ectopic constructs, and subsequently received 5 intraperitoneal injections of DMSO or TMZ (20 mg k^−1^g) from the 21^st^ day post U87 injection. Tumor volume was monitored as indicated from the 21^st^ day. Tumors were collected at 21^st^ day after U87 injection and 15 days post TMZ‐treatment, respectively. I) Representative images of subcutaneous xenografts at the 21^st^ day after U87 injection. The right panel showing the tumor weight analysis (*n* = 5, ^**^ *p* < 0.01). J) Quantitative analysis of tumor volume after TMZ treatment. K) Representative images of subcutaneous xenografts on the 15^th^ day after TMZ treatment (left panel). The right panel shows the tumor weight analysis (*n* = 6, ^**^ *p* < 0.01).L) Quantitative analysis of tumor inhibition ratio of TMZ treatment (*n* = 6, ^**^ *p* < 0.01).

To determine whether Smad1 functions on GBM phenotypes, GBM cell lines were selected to establish Smad1 overexpression and depletion models based on Smad1 protein level (Figure S2A, Supporting Information). Smad1‐targeting CRISPR/Cas9 and corresponding controls were used to knockout (KO) *SMAD1* expression in U87 and U251 cells containing higher levels of endogenous Smad1 (Figure S2B, Supporting Information), whereas Smad1 overexpressing cells were generated from U118 and A172 cell lines with relatively lower Smad1 expression (Figure S2C, Supporting Information). Smad1 KO resulted in significant inhibition of cell proliferation and DNA synthesis as evidenced by cell growth assay (**Figure** **2**A), EdU incorporation (Figure 2B), and colony formation assays (Figure 2C). It also resulted in a reduced number of invasive cells as shown by the invasion assay (Figure 2D). Conversely, ectopic Smad1 expression promoted cell proliferation, DNA synthesis, colony formation, and invasion (Figure 2A–D). To assess the effect of Smad1 on tumorigenesis in vivo, we performed an intracranial xenograft assay in nude mice using U87 cells with Smad1 depletion or control vectors. The tumor volumes were monitored by MRI (Figure 2E). Smad1 depletion resulted in a significant decrease in tumor growth (Figure 2E) and tumor cell proliferation (Figure 2F). We subsequently determined the effect of Smad1 on cell apoptosis using Annexin V/7‐aminoactinomycin D (7‐AAD) staining and flow cytometry. GBM cells were treated with a low dose of Doxorubicin (Dox, 2 µM) for 24 h and apoptotic cells were analyzed. As shown in Figure 2G, Dox induced slight apoptosis of U87 and U251 cells, which mainly occurred in the late stage of apoptosis. Smad1 repression promoted Dox‐induced apoptosis in U87 and U251 cells. On the contrary, Smad1 overexpression decreased Dox‐induced apoptosis in A172 and U118 cells, suggesting the protective effect of Smad1 on cellular apoptosis induced by chemotherapy. Next, xenograft assay in nude mice was used to determine whether Smad1 depletion promotes the chemosensitivity of GBM in vivo. U87 cells with Smad1 depletion or control vectors were injected subcutaneously into nude mice. Figure 2H illustrates the schematic time line of the U87 flank models. Briefly, low dose TMZ (20 mg k^−1^g) was injected 21 days post‐injection for 5 days, once a day. The tumor size was measured every 3 days from the 21st day post‐subcutaneous injection. The tumors were collected 21 days after cell injection (Figure 2I) and on the 15th day after TMZ treatment (Figure 2K), respectively. Consistent with the results of the intracranial xenograft, tumor weight analysis of derived tumors on the 21st day after implantation showed that Smad1 depletion significantly inhibited the growth of subcutaneous transplanted tumors (Figure 2I). As shown in Figure 2J, low dose TMZ resulted in a gradually decreased tumor volume among the two groups, and Smad1 KO induced a significantly smaller tumor at the final timepoint (Figure 2K). Considering the different sizes of Smad1 NC and KO tumors before TMZ treatment, the inhibitory rate was calculated by dividing the tumor volume during treatment by the tumor volume before treatment to compare chemosensitivity (Figure 2L). Smad1 knockout resulted in significant tumor regression, confirming the effect of Smad1 KO on chemosensitivity promotion. Collectively, these data provide evidence that Smad1 is an important oncoprotein regulating GBM tumorigenicity and chemosensitivity.

### 2.3 The Regulation of Smad1 in GBM Mechanically Targets the p53 Pathway

**Figure 3:**
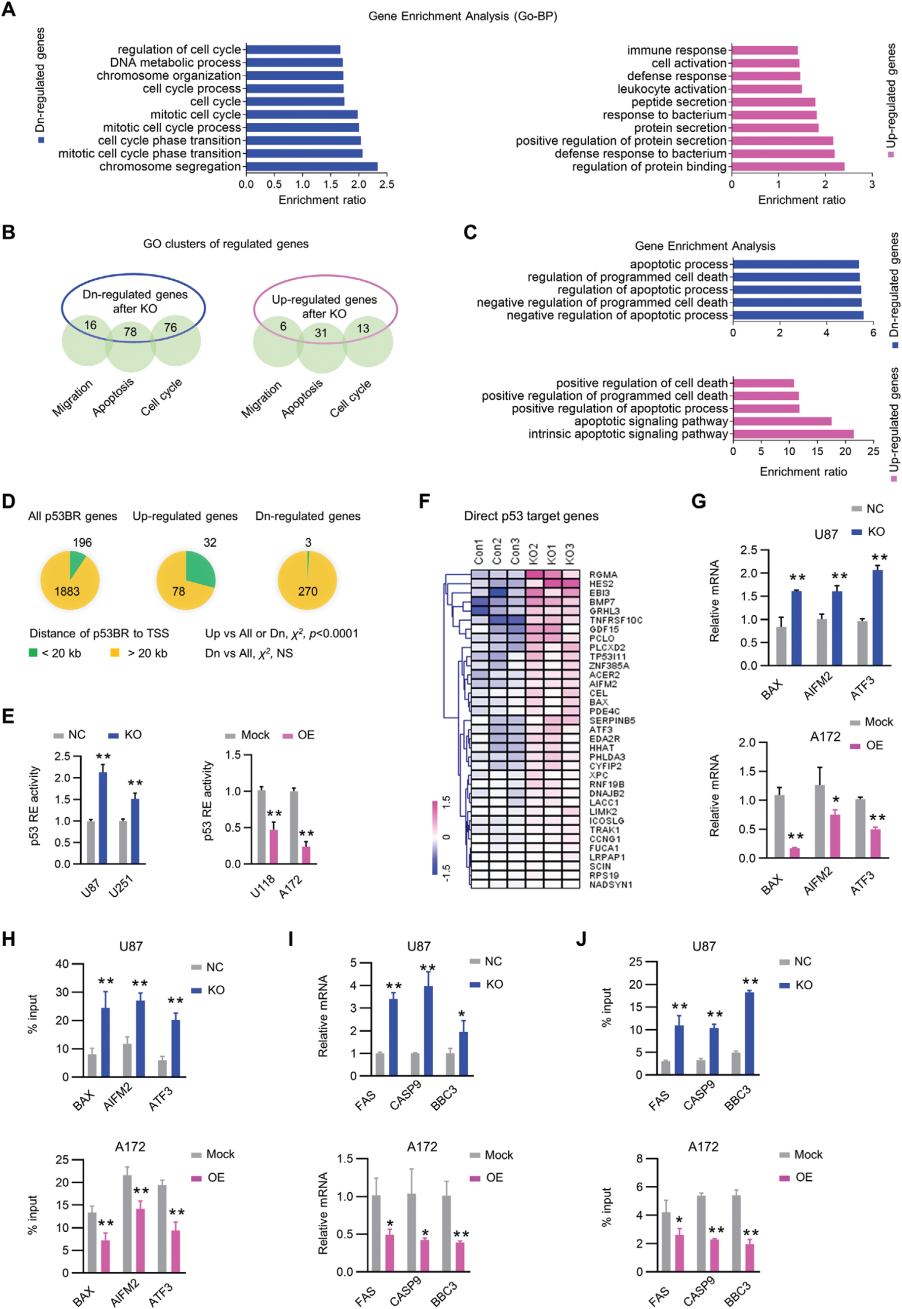
The regulation of Smad1 in GBM mechanically targets p53. A) LncRNA microarray identified Smad1‐regulated genes in different pathways. The histogram indicating the top 10 enriched GO term pathways in Gene Ontology (GO) terms (C5: biological process, BP) according to the significantly changed genes induced by Smad1 depletion (*p* < 0.05). B) The Venn diagram showing the number of Smad1‐regualted genes associated with migration, apoptosis, and cell cycle pathways. C) Gene enrichment analysis indicating the top 5 enriched GO‐BP terms associated with Smad1‐regulated genes collected in B. D) The pie plots showing the number of genes bound by p53 (p53BR to TSS < 20 kb) in the whole genome or in the set of Smad1‐regulated genes (Fisher's exact test). E) Luciferase assay of p53 transcriptional activity in the indicated GBM cells with Smad1 KO (left) or OE (right; *n* = 6, ^**^ *p*<0.01). F) Heat map of direct p53 target genes overlapping significantly upregulated genes after Smad1 depletion. G) qRT‐PCR assay measuring the expression of p53 target genes in GBM cells with Smad1 KO (upper) or OE (lower; *n* = 3, ^*^ *p* < 0.05, ^**^ *p* < 0.01). H) ChIP‐qPCR assay indicating the enrichment of p53 binding to the promoter of target genes in the indicated GBM cells (*n* = 3, ^**^ *p* < 0.01). I) qRT‐PCR assay measuring the expression of p53 targeted genes for apoptosis in GBM cells with Smad1 KO (upper) or OE. Cells were treated with Etoposide (Eto, 10 µM) for 8 h before harvest (lower; *n* = 3, ^*^ *p* < 0.05, ^**^ *p* < 0.01). J) ChIP‐qPCR assay detecting the enrichment of p53 binding to the promoter of target genes in the indicated GBM cells (*n* = 3, ^**^ *p* < 0.01).

To explore the molecular mechanisms underlying Smad1‐mediated tumorigenicity and chemoresistance, we performed cDNA microarray on U87 cells with *SMAD1* depletion or negative control constructs. Applying a 1.5 fold change (*p* < 0.05) as a cutoff, Smad1 KO affected the expression of 1103 genes in U87 cells (604 down‐regulated and 458 up‐regulated; Table S2, Supporting Information). Assessment of Gene Ontology of differentially expressed genes revealed that Smad1 depletion altered several pathways. Genes down‐regulated by Smad1 KO were associated with chromosome organization and cell cycle process, while those up‐regulated by Smad1 depletion were associated with protein binding and secretion (**Figure** **3**A). Smad1 regulated genes involved in functional processes, including cell cycle, apoptosis, and migration, were collected for further analysis (Figure 3B; Table S3, Supporting Information). The enriched top 5 GO‐BP terms indicated the collected down‐regulated genes induced by Smad1 depletion were negatively associated with apoptotic processes and cell death, while the upregulated genes induced by Smad1 depletion positively regulated apoptotic process and cell death (Figure 3C), reinforcing the regulation of Smad1 in chemosensitivity. p53 plays crucial roles in regulating cell cycle, cell apoptosis, and genome stability,^[^ ^19^, ^20^ ^]^ and is deeply involved in the TGF‐β signaling pathway for cell‐fate decisions and cellular homeostatic via acting as an essential partner of Smads.^[^ ^21^ ^]^ Moreover, Smad1 has been documented as an important participant in DNA damage response via involving the Atm‐p53 pathway,^[^ ^22^ ^]^ and is a culprit for colorectal cancer chemoresistance.^[^ ^23^ ^]^ These findings underscore the significance of Smad1‐p53 interaction in tumorigenesis and chemoresistance of cancers. To explore whether Smad1 regulates p53 activity in GBM cells, we further analyzed the occupancy of p53 on the promoters of Smad1 depletion‐induced genes using chromatin immunoprecipitation sequencing (ChIP‐seq) data published previously (GSE46641).^[^ ^24^ ^]^ Of the 2079 genes containing p53 binding peaks, the distance between the p53‐binding region (p53BR) and gene transcription starting site (TSS) among 196 genes was less than 20 kb. Interestingly, 29% (32/110) of the Smad1 depletion‐induced genes had p53 binding regions within 20 kb (Table S4, Supporting Information), a rate significantly higher than the average p53 occupancy in the genome of Smad1 depletion‐reduced genes (*p* < 0.0001, *χ* ^2^) (Figure 3D). We next examined the effects of Smad1 on p53 transcriptional activity using luciferase reporter and qRT‐PCR assay. Using a luciferase reporter containing a p53‐responsive promoter sequence, we observed that Smad1 overexpression or depletion significantly increased or decreased the transcriptional activity of p53 (Figure 3E). To detect the expression of p53 transcriptional targets, we listed genes upregulated after Smad1 KO (*p* < 0.05), identifying those which overlapped as direct p53 target genes (Figure 3F). ^[^ ^25^ ^]^ Three known tumor suppressive genes (*BAX*, *AIFM3, and ATF3*) were selected to evaluate the regulation of Smad1 on p53 transcriptional activity. In the subsequent qRT‐PCR assay, we observed that Smad1 depletion significantly increased mRNA expression of p53 target genes, while Smad1 overexpression suppressed the expression of these genes (Figure 3G). Moreover, we analyzed whether Smad1 inhibited the binding of p53 to target promoters by quantitative ChIP (qChIP). As a result, Smad1 depletion significantly increased the binding of p53 to target promoters, and Smad1 overexpression resulted in a decrease of p53 binding to target promoters (Figure 3H). To determine whether Smad1 promotes chemoresistance via p53 regulation, p53 target genes for apoptosis were examined in Smad1 KO cells treated with Etoposide (Eto). As shown in Figure 3I, FAS, CASP9, and BBC3 were significantly upregulated in Eto treated GBM cells with Smad1 depletion, and qChIP assay indicated an amplification of p53 transactivation in regulating apoptotic genes in the absence of Smad1 (Figure 3J). Conversely, the results obtained in Smad1 OE cells showed the opposite effect. Collectively, these findings suggest that Smad1 exerts oncogenic functions in GBM, which is associated with its negative regulation of p53 transcriptional activity.

### 2.4 Smad1 Binds and Inhibits p53 Acetylation via MH1 and MH2

**Figure 4:**
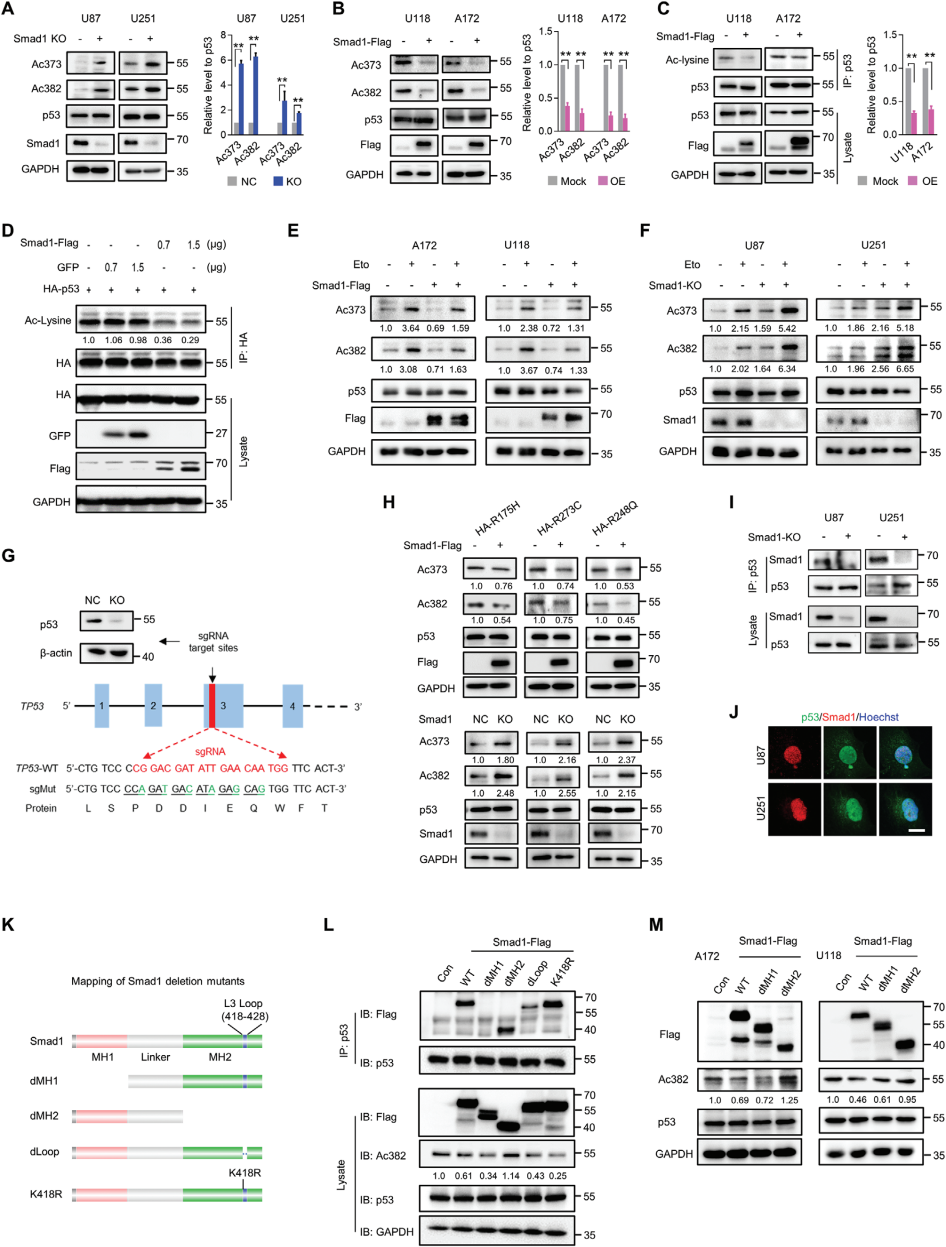
Smad1 binds and impairs p53 acetylation via MH1 and MH2. A) Western blot analysis of p53 Lys373 and Lys382 acetylation (Ac373, Ac382) in U87 and U251 cells with Smad1 KO (left) and the statistical interpretation (right; *n* = 3, ^**^ *p* < 0.01). B) Western blot analysis of p53 acetylation in U118 and A172 cells with Smad1 OE (left) and the statistical interpretation (right; *n* = 3, ^**^ *p* < 0.01). C) Immunoprecipitation (IP) analysis indicating the inhibition of Smad1 on overall p53 acetylation in GBM cells (*n* = 3, ^**^ *p* < 0.01). IP was performed using p53 antibody and the overall acetylation of p53 was detected by immunoblot (IB) using Acetylated‐Lysine antibody (Ac‐Lysine). The input amounts of Smad1 and p53 were also detected as controls using the indicated antibodies. D) IP analysis indicating the inhibition of Smad1 on overall p53 acetylation in 293T cells transfected with indicated vectors. The GFP vector worked as an unrelated control protein. The relative quantification of p53 acetylation was listed under the bands. E) Western blot assay showing Smad1 OE represses p53 acetylation in Etoposide treated cells. Indicated cells were treated with Etoposide (Eto, 10 µM) for 8 h before harvest. The relative quantification of indicated proteins were listed. F) Western blot assay showing Smad1 KO amplifies p53 acetylation in Etoposide treated cells. Indicated cells were treated with Etoposide (Eto, 10 µM) for 8 h before harvest. The relative quantifications of indicated proteins were listed. G) The schematic showing the information of sgRNA targeting p53 DNA sequence and synonymous mutation targeting sgRNA for p53 re‐expression construct (sgMut). Upper left illustration showing the Western blot analysis of p53 in U87 cells with p53 KO. β‐actin worked as a loading control. H) Western blot analysis of p53 acetylation in p53 KO‐U87 cells re‐expressing indicated p53 mutants (upper) with Smad1 OE (upper) or KO (lower). The relative quantifications of indicated proteins were listed. I) IP analysis showing the interaction between Smad1 and p53 in U87 and U251 cells. J) Double IF staining of Smad1 and p53 in U87 and U251 cells. Hoechst stained the nuclei. Bars, 20 µm. K) Schematic diagram of Smad1 protein and its domain deletion mutants or point mutant. L) Mapping the domain of Smad1 for its interaction with p53. 293T cells were co‐transduced with Flag‐tagged Smad1 mutants, followed by IP with anti‐HA antibody and IB analysis with the indicated antibodies. The input amounts of Ac382, p53, Smad1, and GAPDH were also detected as controls using the indicated antibodies. The relative quantification of indicated protein was listed. M) Mapping the domain of Smad1 for its inhibition on p53 acetylation in the indicated GBM cells. The relative quantification of indicated protein was listed.

GSEA results of GO BP (Figure 1D) and GO MF (Table S5, Supporting Information) evaluation based on TCGA‐GBM datasets indicated that genes correlated with Smad1 expression are related to histone deacetylation processes, p53‐binding, and histone acetyltransferase binding. Given that acetylation is critically required for the activation of p53 and subsequent biological processes,^[^ ^10^, ^26^ ^]^ we supposed that Smad1 may exert an inhibitory effect on p53 acetylation. To this end, the alteration of p53 acetylation in GBM cells with Smad1 ectopic expression or depletion was examined. Here, p53 acetylation antibodies targeting two lysine sites (lysine 373 and 382) in the C‐terminal domain (CTD) of p53 were used to monitor the acetylation of p53. Smad1 depletion (**Figure** **4**A) was enhanced but Smad1 overexpression (Figure 4B) decreased p53 acetylation in the indicated GBM cells. Moreover, IP analysis using an acetylated‐lysine antibody revealed that Smad1 overexpression inhibited overall p53 acetylation in U118 and A172 cells (Figure 4C), as well as in 293T cells expressing ectopic p53 and Smad1 (Figure 4D), indicating that Smad1 impairs p53 acetylation across a wide range of lysine sites. Acetylation has been shown to extend p53 half‐life by inhibiting the E3 ligase MDM2 mediated ubiquitination and proteasomal degradation.^[^ ^27^, ^28^ ^]^ However, no significant changes in p53 ubiquitination (Figure S3A, Supporting Information) and half‐life (Figure S3B, Supporting Information) were observed in GBM cells with Smad1 KO or OE. To establish whether Smad1 inhibits the newly occurring acetylation of p53, Eto was utilized as an acetylation inducer.^[^ ^29^, ^30^ ^]^ Figure 4E illustrates that Smad1 overexpression significantly reduced the acetylation of p53 in U118 and A172 cells induced by Eto, while it was notably increased in Smad1 KO cells (Figure 4F). The finding that Smad1 inhibited p53 acetylation in U251 (p53 R273H) and U118 (p53 R213Q) containing p53 missense mutations suggests that Smad1 may exert a negative regulatory effect on the acetylation of both wild‐type and mutant p53. To further confirm the finding that Smad1 impairs the acetylation of mutant p53, we established a p53 KO‐U87 cell line mediated by CRISPR/Cas9 (Figure 4G). Based on this p53‐depleted cell line, the top three p53 hotspot missense mutants in human brain cancers (data were derived from http://www.cancerhotspots.org),^[^ ^31^, ^32^ ^]^ including R273C, R175H, and R248Q, were introduced with the aid of site mutations according to the sgRNA sequence (sgMut). As shown in p53 KO‐U87 cells re‐expressing indicated p53 mutants (Figure 4H), Smad1 overexpression resulted in a decrease, while Smad1 KO led to an increase of mutant p53 acetylaiton. Subsequently, the endogenous interaction between p53 and Smad1 was determined by IP assay in U87 and U251 cells with Smad1 KO (Figure 4I). This interaction occurred in the nuclei (Figure 4J). To determine the interactive region of Smad1 with p53, we established Smad1 truncated mutants according to the features of the Smad1 protein domain derived from UniProt (https://www.uniprot.org) (Figure 4K). The IP analysis showed that Smad1 without the MH2 domain (dMH2, depleting residues 182–496) exhibited comparable p53‐binding ability as full‐length Smad1, whereas MH1 deletion mutants (dMH1, depleting residues 1–331) completely abolished the p53 binding ability (Figure 4L), indicating the necessity of MH1 for Smad1 binding of p53. Considering that the L3 Loop is an important structure mediating the interaction between SMADs and other proteins,^[^ ^33^, ^34^, ^35^ ^]^ the role of the L3 Loop in the interaction between Smad1 and p53 was explored. Although located in the MH2 domain, L3 Loop depletion (dLoop) markedly impaired the p53‐binding ability of Smad1, indicating that the L3 Loop is another important domain affecting Smad1 binding of p53. An additional observation was that the lysine mutant of the L3 Loop (K418R) did not affect the binding of Smad1 to p53. An unexpected observation was that neither the loss of MH1 nor the L3 Loop was able to reverse the inhibition of Smad1 on p53 acetylation, suggesting the inhibition of Smad1 on p53 acetylation is not determined by its p53 binding capacity. In contrast, the deletion of MH2 led to the removal of the inhibitory impact of Smad1 on p53 acetylation. Accordingly, the effect of MH1 and MH2 in Smad1‐mediated inhibition of endogenous p53 acetylation in A172 and U118 cells was further confirmed (Figure 4M). Collectively, these findings suggest that Smad1 binds p53 through the MH1 domain, but hinders p53 acetylation through the MH2 domain.

### 2.5 Smad1 Inhibits p53 Acetylation via p300‐Hijacking Through the MH2 Domain

**Figure 5:**
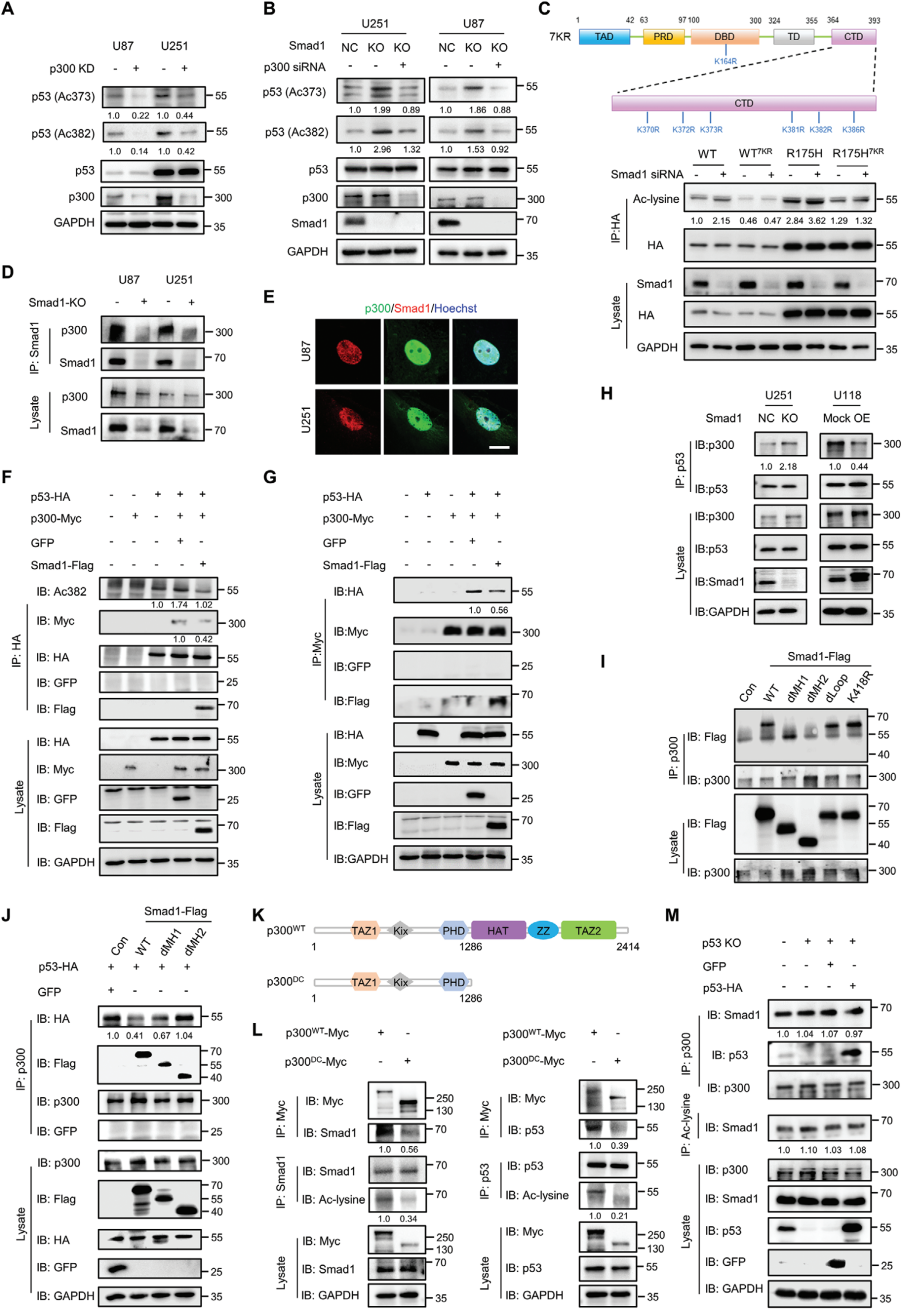
Smad1 inhibits p300 mediated p53 acetylation via p300‐hijacking through the MH2 domain. A) Western blot analysis of p53 acetylation in GBM cells with p300 knockdown (KD). Indicated cells were transfected with p300 siRNA and the level of p53 acetylation and p300 were detected. The relative quantifications of p53 acetylation were listed under the bands. B) Western blot analysis indicating p300 KD inhibited the increase of p53 acetylation induced by Smad1 KO. The relative quantifications of p53 acetylation were listed under the bands. C) Schematic diagram showing p53 protein and 7 lysine residues (K164, K370, K372, K373, K381, K382 and K386) substituted with Arg (R). Lower panel showing the IP analysis of p53 acetylation in p53 KO‐U87 cells re‐expressing indicated HA‐tagged p53 construct. The relative quantification of p53 acetylation was listed. D) IP assay showing the interaction between Smad1 and p300 in GBM cells. E) Double IF staining of Smad1 and p300 in U87 and U251 cells. Hoechst stained the nuclei. Bars, 10 µm. F) IP assay indicating the repression of Smad1 on p300 mediated p53 acetylation. 293T cells were co‐transfected with HA‐p53 and p300‐Myc vectors with or without ectopic Smad1 expression, followed by IP and Western blot analysis using the indicated antibodies. The relative quantifications of p53 acetylation and p300 binding were listed under the bands. G) IP assay showing the inhibitory effect of Smad1 on the interaction between p53 and p300. 293T cells were transfected with the indicated vectors followed by IP using HA antibody. The relative quantification of binding p53 was listed under the bands. H) IP assay showing the inhibitory effect of Smad1 on the endogenous interaction between p53 and p300 in GBM cells with Smad1 KO or OE. The relative quantification of p300 binding was listed under the bands. I) Mapping the domain of Smad1 for its interaction with p300. 293T cells were transfected with Flag‐tagged Smad1 mutants, followed by IP with anti‐p300 antibody and IB analysis with the indicated antibodies. J) Mapping the domain of Smad1 for its effect on the interaction of p53 and p300. 293T cells were co‐transfected with Flag‐tagged Smad1 mutants and HA‐p53, followed by IP with anti‐p300 antibody and IB analysis with the indicated antibodies. The relative quantification of p53 binding was listed under the bands. K) Schematic diagram showing p300 protein and the C‐terminal deletion mutant (p300^DC^). L) IP assay detecting p300 binding Smad1 or p53. U87 cells were transfected with Myc‐p300^WT^ or Myc‐p300^DC^ and followed with IP using indicated antibodies. The relative quantifications of indicated proteins were listed. M) IP assay detecting the interaction between p300 and Smad1, and the acetylation of Smad1 in p53 KO‐U87 cells and p53 re‐expressing cells. U87 with p53 KO was re‐introduced with a p53 construct, the harvested cell lysates were evaluated by IP using p300 or Ac‐lysine antibody. GFP vector served as an unrelated control protein. The relative quantifications of Smad1 were listed.

Due to the physical interaction between the acetyltransferase p300 and Smad1,^[^ ^36^, ^37^, ^38^ ^]^ we examined whether Smad1 inhibits p53 acetylation by impairing p300‐mediated p53 acetylation. In U87 and U251 cells, p300 knock‐down by siRNA resulted in a decrease of p53 acetylation (**Figure** **5**A), and further abolished the increase of p53 acetylation induced by Smad1 depletion (Figure 5B), indicating the involvement of p300 in p53 acetylation in GBM cells. To further confirm the inhibitory effect of Smad1 on overall p300 mediated p53‐acetylaiton, p53 mutants containing a non‐acetylated site mutation (7KR) were constructed, in which 7 lysine residues (K164, K370, K372, K373, K381, K382, and K386) regulated by p300 were converted to Arg (R).^[^ ^39^ ^]^ Considering R175H has the highest occurrence in cancer patients among the hotspot p53 mutations,^[^ ^40^ ^]^ the lentivirus expressing R175H was used to represent a missense mutant p53. The IP analysis with acetylated‐lysine antibody showed that the overall acetylation of wild‐type and R175H evoked by Smad1 knockdown in U87‐p53 KO cells was blocked in 7KR constructs (Figure 5C). This indicated that Smad1 suppresses p300 mediated p53 acetylation overall. Next, we probed the endogenous interaction between Smad1 and p300 (Figure 5D), observing that this interaction occurred in the nuclei of GBM cells (Figure 5E). Subsequent reciprocal IP assay in 293T cells expressing ectopic p53 and p300 showed that Smad1 introduction impaired the interaction between p53 and p300, and subsequent p53 acetylation (Figure 5F,G). In U251 and U118 cells, IP assay showed that Smad1 depletion resulted in an increase of p53 and p300 binding, while ectopic Smad1 weakened the binding of p53 and p300 (Figure 5H). Next, the binding region of Smad1 to p300 was identified based on the truncated mutants of Smad1 (Figure 5I). It showed that MH2 depleted Smad1 (dMH2) failed to bind p300, though other domain deletions or point mutations had no observable effects on Smad1 and p300 interaction. This indicated that MH2 is essential for Smad1 binding p300. Subsequent IP assay in 293T cells co‐expressing ectopic p53 and Smad1 deletion mutants demonstrated that dMH2 failed to inhibit the interaction between p53 and p300, while dMH1 possessed the capacity to inhibit the interaction of p53 and p300 (Figure 5J). These observations, as well as the finding that p300 knockdown had no effect on p53 and Smad1 interaction (Figure S4, Supporting Information), addressed an underlying mechanism of competitive binding between p53 and Smad1 on the same p300 domain. Given the evidence that the C‐terminal domain is critical for the p53‐binding of p300,^[^ ^41^, ^42^ ^]^ a C‐terminal deletion mutant of p300 (del 1287–2414aa; Myc‐p300^DC^) was established (Figure 5K). The IP results showed that both p53 and Smad1 lost the ability to bind p300^DC^ in U87 cells, confirming the assumption that p53 and Smad1 bind p300 at the same domain (Figure 5L). As a result, Myc‐p300^DC^ lost its regulatory ability on p53 acetylation. An interesting finding was that significant Smad1 acetylation was observed in cells expressing ectopic p300^WT^, but not in p300^DC^ expressing cells, suggesting that p300 is a HAT conferring Smad1 acetylation. Considering that competitive inhibition is normally a reciprocal process, we further explored whether p53 inhibits Smad1 binding p300 in U87 cells with depleted p53 and in p53 re‐expressing cells. It showed that the interaction between Smad1 and p300 remained unchanged in cells with p53‐depletion and even in cells with p53 re‐expression (Figure 5M). In other words, p300 bound Smad1 cannot be substituted by p53 in GBM cells. Combined with the observations in Figure 4L,M, these results document that the inhibitory effect of Smad1 on p53 acetylation is dependent on MH2‐mediated p300 adsorption, thus preventing p300 binding and p53 acetylation.

### 2.6 p300‐Mediated Acetylation is Vital for Smad1 Oncofunctions

**Figure 6:**
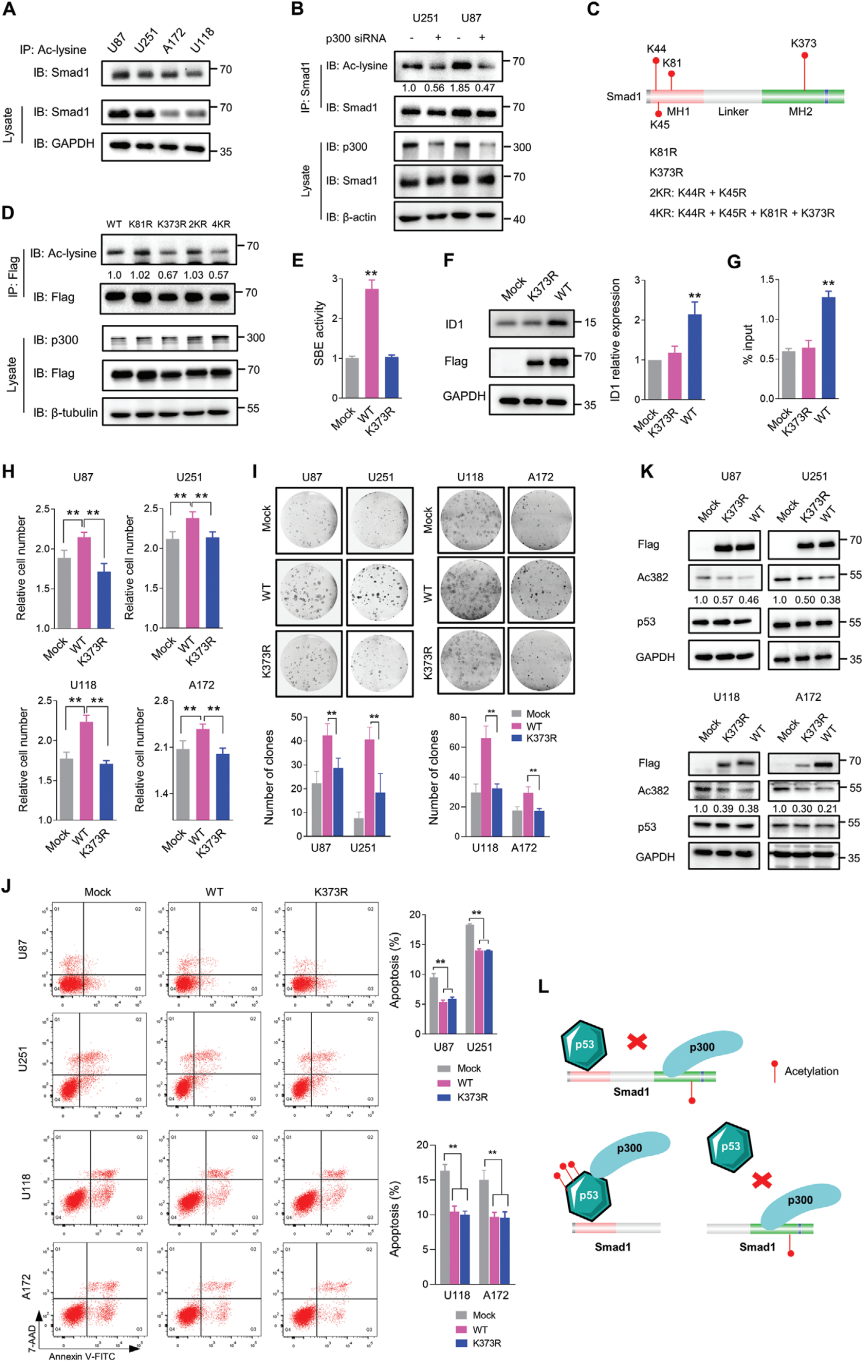
p300‐mediated acetylation is vital for Smad1 oncofunctions. A) IP assay detecting the acetylation of Smad1 in indicated GBM cell lines. B) IP assay indicating Smad1 acetylation is regulated by p300. Indicated cells were transfected with p300 siRNA, followed by IP using anti‐Smad1 antibody and IB using the indicated antibodies. The relative quantification of Smad1 acetylation was listed. C) Schematic diagram of p300‐specific acetylation site prediction of Smad1 and its mutants with unit point or multi‐site mutation of the indicated Lysine (K) residues converted to Arginine (R). D) IP assay detecting the acetylation of Smad1 mutants. U87 cells were transfected with indicated Smad1 constructs, followed by IP using anti‐Flag antibody and IB using the indicated antibodies. The relative Smad1 acetylation was listed under the bands. E) SBE reporter assay monitoring the activity of SMAD responsive transcriptional regulation in U87 cells expressing the indicated constructs (*n* = 3, ^**^ *p* < 0.01). F) Western blot analysis of ID1 expression in U87 cells expressing the indicated constructs (*n* = 3, ^**^ *p* < 0.01). G) ChIP‐qPCR assay detecting the enrichment of p53 binding to the promoter of *ID1* in U87 expressing different Smad1 constructs (*n* = 3, ^**^ *p* < 0.01). H) Cell growth assay of p53 KO cells overexpressing indicated Smad1 constructs. Cells were cultured for 48 h, and followed with a CCK‐8 assay to detect cell number (*n* = 6, ^**^ *p* < 0.01). I) Representative colony formation (left panel) of p53 KO cells overexpressing indicated Smad1 constructs (*n* = 3, ^**^ *p* < 0.01). J) Apoptosis assay of indicated GBM cells. Cells were treated with Dox (2 µM) for 24 h and apoptotic cells were analyzed by FACS (*n* = 3, ^**^ *p* < 0.01). K) Western blot analysis detecting p53 acetylation in indicated GBM cells expressing different Smad1 constructs. The relative quantifications of p53 acetylation were listed. L) Diagram illustrating the relationship among p53, Smad1 and p300 in regulating acetylation of p53 and Smad1. Smad1 binds p53 and p300 via MH1 and MH2 domain, respectively, and destroys the interaction of p53 and p300. MH2 is essential, but MH1 is dispensable for Smad1 mediated p53 acetylation inhibition.

Considering the interaction between Smad1 and p300, we speculated that Smad1 is acetylated by p300 in GBM cells. To this end, the acetylation of Smad1 in GBM cell lines was examined by IP assay using an acetylated‐lysine antibody. As shown in **Figure** **6**A, there was considerable acetylation of Smad1 in GBM cell lines, and p300 knock‐down resulted in an obvious decrease of Smad1 acetylation in U87 and U251 cells (Figure 6B). To explore the acetylation sites of Smad1 regulated by p300, candidate sites were predicted using ASEB, a web service of the lysine‐acetyl‐transferase (KAT)‐specific acetylation site prediction platform (http://cmbi.bjmu.edu.cn/huac).^[^ ^43^ ^]^ Results suggested that p300 could potentially acetylate Smad1 at 4 lysine sites. Based on these lysine sites, we constructed unit point and multi‐site mutants to act as potential non‐acetylation mimics of Smad1, converting the indicated lysine residues to Arginine (R) (Figure 6C). These mutants were expressed in U87 cells, and the acetylation of ectopic Smad1 was detected by IP assay using an acetylated‐lysine antibody (Figure 6D). Findings revealed that the acetylation of K81R, 2KR was comparable to that of wild‐type Smad1, while the acetylation of mutants containing K373R (including K373R and 4KR) was repressed, suggesting K373 is an important site for Smad1 acetylation. Subsequently, the SBE reporter assay indicated that Smad1 overexpression resulted in a significant promotion of SMAD responsive transcriptional activity, while K373R lost this promotion (Figure 6E). Accordingly, K373R failed to induce the expression of ID1, a typical SMAD target gene that was markedly induced by wild‐type Smad1 overexpression (Figure 6F). The ChIP assay revealed that the ectopic overexpression of Smad1 induced a marked increase in Smad1 enrichment at the ID1 promoter, while the introduction of K373R did not result in the same effect (Figure 6G). This provides further evidence that Smad1 directly binds the DNA of target genes, and acetylation modification is essential for Smad1 responsive transcriptional regulation. Subsequent functional study in p53‐KO GBM cells revealed the oncogenic impact of Smad1 independently on p53, and K373R losing the tumor promotion further indicated the importance of acetylation in mediating growth promotion of Smad1 (Figure 6H,I). However, in p53‐KO GBM cells, either wild‐type Smad1 or K373R overexpression failed to exhibit a protective effect against chemotherapy (Figure S5, Supporting Information). On the contrary, in naïve GBM cells, K373R introduction demonstrated its promotion in tumor growth (Figure S6, Supporting Information) and its anti‐apoptotic effect as wild‐type Smad1 done (Figure 6J). Moreover, K373R retained the inhibition on p53‐acetylation (Figure 6K) as wild‐type Smad1 demonstrated in GBM cells, indicating that the acetylation status of Smad1 does not affect its inhibition on p53 acetylation. Thus, these results illustrated that Smad1 contains the pro‐carcinogenic impact unrelated to p53 in tumor growth, while its ability to protect against chemotherapy relies on p53. Along with the observation in Figure 6M that neither p53 depletion nor re‐expression affected Smad1 acetylation in GBM cells, it demonstrates that Smad1 and p53 compete for p300 mediated acetylation, while Smad1 has the priority over p53 (Figure 6L).

### 2.7 Smad1 is Negatively Associated with p53 Acetylation in Primary GBM

**Figure 7:**
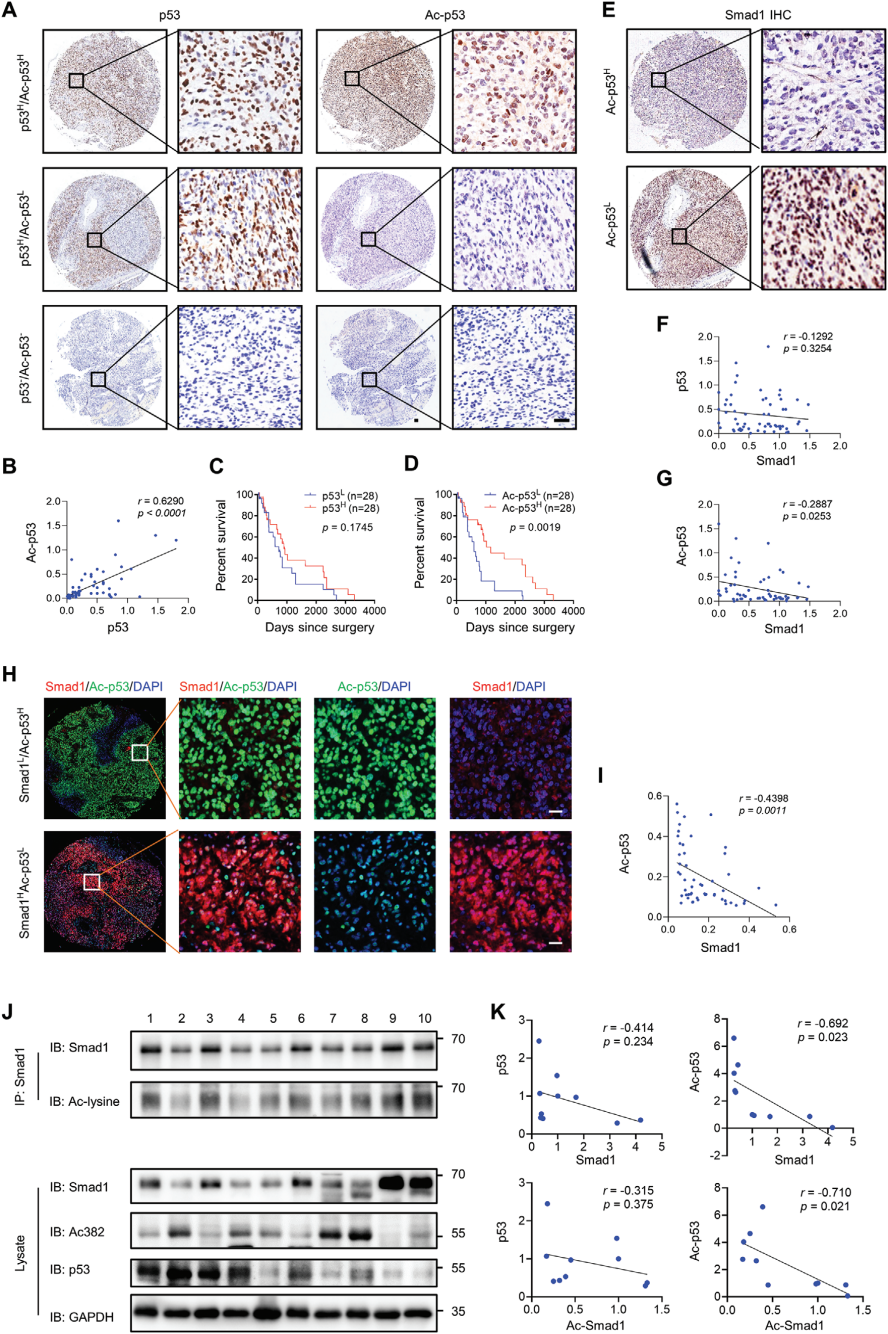
Smad1 is negatively associated with p53 acetylation in primary GBM. A) Representative images of IHC staining of p53 and acetylated p53 (Ac‐p53) in patient‐derived GBM tissue array. Scale bars, 50 µm. B) Correlation analysis between p53 and Ac‐p53 expression (Spearman correlation analysis). C) Overall survival analysis based on p53 expression. Groups were ranked according to p53 median of IRS in GBM tissues. D) Overall survival analysis based on Ac‐p53 levels. Groups were ranked according to Ac‐p53 median of IRS in GBM tissues. E) Representative sections of Smad1 IHC according to low or high Ac‐p53 expression. Scale bars, 50 µm. F) Correlation analysis of p53 and Smad1 expression (Spearman correlation analysis). G) Correlation analysis of Ac‐p53 and Smad1 expression (Spearman correlation analysis). H) Representative images of multiplex immunohistochemistry of Smad1 and Ac‐p53 on the same GBM tissue sections. DAPI labeled nuclei. Scale bars, 50 µm. I) Correlation analysis of Ac‐p53 and Smad1 expression in multiplex immunohistochemistry of GBM tissues (Spearman correlation analysis). J) IP and Western blot assay detecting the acetylation of Smad1 and p53 acetylation in primary GBM tissues using indicated antibodies. K) Correlation analysis of indicated proteins (Spearman correlation analysis).

To further investigate the role of Smad1 in regulating p53 acetylation in vivo, the correlation between Smad1 and p53 acetylation in the GBM tissue array was examined using immunohistochemistry (**Figure** **7**A). A positive correlation between the level of acetylated p53 and p53 expression was observed (Figure 7B). There was no correlation between p53 protein levels and survival of GBM patients based on the cutoff at median expression (Figure 7C; Supplemental Data 2, Supporting Information). On the other hand, a significant correlation was observed between p53 acetylation and survival outcomes (Figure 7D; Supplemental Data 3, Supporting Information). Considering the high mutation rate of *TP53* in GBM,^[^ ^44^ ^]^ these findings suggest that acetylation is a benign indicator even in patients containing mutant p53. Next, we analyzed the correlation between Smad1 and p53 in serial cross‐sections of GBM tissue stained by Smad1, p53, and acetylated p53, respectively (Figure 7E). Although no direct association between p53 and Smad1 was detected (Figure 7F), a negative correlation was observed between Smad1 and p53 acetylation (Figure 7G). To further confirm the findings, we evaluated the correlation between Smad1 and acetylated p53 (Acetylated Lys382) by multiplex immunohistochemistry in the same GBM tissue sections (Figure 7H). The quantitative analysis confirmed the negative relationship between Smad1 and p53 acetylation (Figure 7I). Finally, Western blot and immunoprecipitation analysis of primary GBM tissues (Figure 7J) also supported these findings, showing that the expression of Smad1 was not correlated with p53, but was negatively associated with p53 acetylation (Figure 7K). Additionally, there was a negative association between acetylated p53 and acetylated Smad1, suggesting a competition for acetylation between the two proteins.

### 2.8 Smad1‐p300 Binding Contributes to the Chemoresistance of GBM Cells Containing Missense Mutant p53

**Figure 8:**
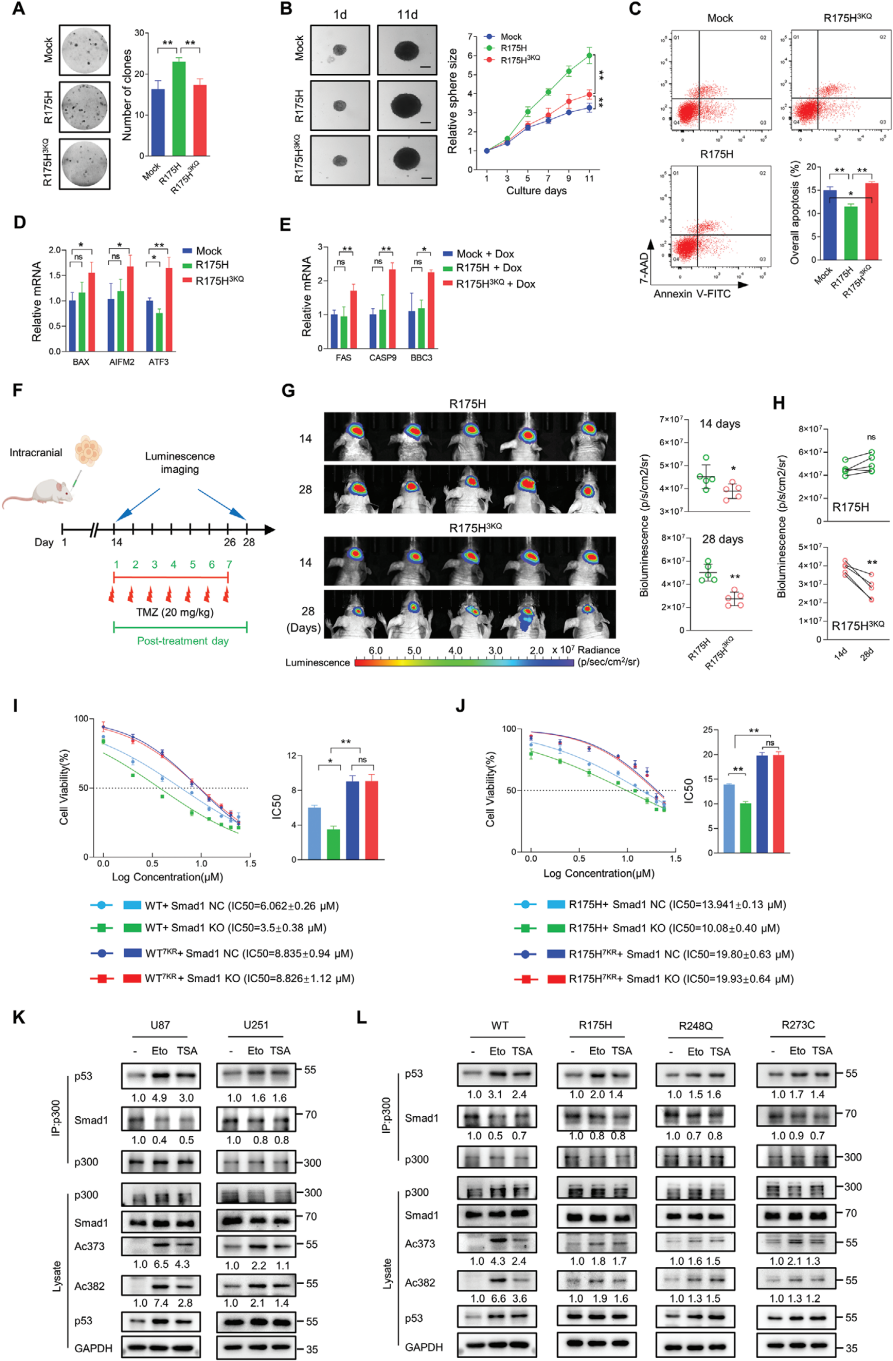
Acetylation alleviates gain of function and chemotherapy resistance of missense mutant p53. A) Colony formation assay of p53 KO‐U87 cells re‐expressing indicated p53 mutants (*n* = 3, ^**^ *p* < 0.01). B) Tumor sphere growth assay of p53 KO‐U87 cells re‐expressing indicated p53 mutants (*n* = 5, ^**^ *p* < 0.01). Bars, 200 µM. C) Apoptosis assay of U87 cells expressing the indicated p53 constructs. Cells were treated with Dox (2 µM) for 24 h and cellular apoptosis was analyzed by FACS. Quantitative analysis showing the overall apoptosis ratio (*n* = 3, ^*^ *p* < 0.05, ^**^ *p* < 0.01). D) qRT‐PCR assay detecting the expression of p53 targeted genes in p53 KO‐U87 cells expressing indicated p53 constructs (*n* = 3, ^*^ *p* < 0.05, ^**^ *p* < 0.01). E) qRT‐PCR assay measuring the expression of p53 targeted genes for apoptosis in p53 KO‐U87 cells expressing indicated p53 constructs. Cells were treated with Dox (2 µM) for 24 h before harvest (*n* = 3, ^*^ *p* < 0.05, ^**^ *p* < 0.01). F) Schematic illustration of the evaluation of in vivo tumor growth derived from U87 cells to TMZ treatment. Mice were intracranially injected with U87 cells expressing R175H or R175H^3KQ^, subsequently receiving 7 intraperitoneal injections of DMSO or TMZ (20 mg k^−1^g) from the 14^th^ day post‐implantation. Tumor volume was monitored at 14^th^ and 28^th^ days post‐implantation using luminescence imaging. G) Images of the derived intracranial tumors and the statistical results of tumor volume (*n* = 5, ^*^ *p* < 0.05, ^**^ *p* < 0.01). H) The statistical results of tumor volume before and after treatment (*n* = 5, ^**^ *p* < 0.01). I) IC50 assay of U87‐p53‐KO cells re‐expressing wild‐type p53 or 7KR mutants with or without Smad1 KO. Cells were treated with Etoposide (Eto, 0, 1, 2, 4, 8, 12, 16, 20, 24 µM,) for 48 h. The bar graph (right panel) shows the IC50 value of *n* = 3 independent experiments (^*^ *p* < 0.05, ^**^ *p* < 0.01). J) IC50 assay of p53 KO‐U87 cells re‐expressing R175H or its 7KR mutants with or without Smad1 KO. Cells were treated with Etoposide (Eto, 0, 1, 2, 4, 8, 12, 16, 20, 24 µM,) for 48 h. The bar graph (right panel) shows the IC50 value of *n* = 3 independent experiments (^**^ *p* < 0.01). K) IP assay detecting the interaction between p300 and p53 or Smad1 in U87 and U251 cells treated with Eto (10 µM) or Trichostatin A (TSA, 2 µM) for 8 h. IP was performed using p300 antibody, and the input amounts of Smad1, p53 and acetylated p53 were detected using indicated antibodies. The relative quantifications of indicated proteins were listed under the bands. L) IP assay detecting the interaction between p300 and p53 or Smad1 in p53 KO‐U87 re‐expressing wild‐type p53 or indicated mutants. The relative quantifications of indicated proteins were listed.

The consensus is that *TP53* missense mutations are associated with malignant cancer phenotypes.^[^ ^45^ ^]^ Based on p53‐KO U87 cells, three hot missense mutants (R175H, R273C, and R248Q) and wild‐type p53 (WT) were introduced using site‐directed mutagenesis with the aid of sgRNA sequences (sgMut). As demonstrated in Figure S7 (Supporting Information), cells harboring p53 missense mutants show superior characteristics in cell growth, proliferation, invasion, and resistance to chemotherapy when compared to cells containing wild‐type p53. Given that acetylation restores mutant p53 DNA binding and transcriptional regulation,^[^ ^14^, ^15^, ^17^ ^]^ our findings in p53 mutant‐containing U251 and U118 cells suggest that the inhibitory effect of Smad1 on p53 acetylation is the basis for the oncogenic effects of mutant p53. The established CTD constitutive acetylation mimics (3KQ) constructed based on wild‐type and R175H (R175H^3KQ^)^[^ ^17^ ^]^ were used to consolidate the understanding. As shown in colony formation (**Figure** **8**A) and 3D‐spheroids assay (Figure 8B), R175H introduction significantly promoted cell growth of p53‐KO U87 cells, while the acetylation simulator (R175H^3KQ^) reversed the effects of R175H. In terms of cellular apoptosis, R175H showed a notable protective effect against Dox‐induced apoptosis, which was then counteracted by its acetylated simulator (Figure 8C). For p53 regulation, R175H did not have the ability to increase the expression of tumor suppressor genes, instead, it has a partially repressive effect. Conversely, R175H^3KQ^ significantly promotes the expression of these tumor suppressors (Figure 8D). In the absence of endogenous p53, treatment with Dox failed to induce changes in the expression of FAS, CASP9, and BBC3 in cells with ectopic R175H. However, in cells with the R175H^3KQ^ mutation, Dox did lead to an increase in the expression of these genes (Figure 8E), suggesting that acetylation may compensate for the loss of p53 activity resulting from missense mutation. To further confirm the in vitro findings, an in vivo tumorigenesis assay was performed using luciferase labeled p53‐KO U87 cells expressing the indicated constructs. Cells were stereotactically implanted into the right hemisphere of the mouse brain and received TMZ treatment once every 2 days from the 14^th^ day post‐implantation (total 7 doses). Intracranial tumorigenesis was monitored by bioluminescence imaging before and 2 days after the last treatment (Figure 8F). Results showed that cells expressing the p53 acetylation mimetic developed relatively smaller tumors in mice both at the start and end of TMZ‐treatment (Figure 8G). Moreover, comparing the volume of the same tumor before and after treatment indicated a greater sensitivity to TMZ in tumors expressing R175H^3KQ^ (Figure 8H). Accordingly, the introduction of wild‐type p53, along with its 3KQ mutant, led to a decrease in tumor growth (Figure S8A,B, Supporting Information) and an enhancement of chemosensitivity both in vitro and in vivo (Figure S8C–E, Supporting Information), with 3KQ showing even greater benefits, which was also reflected in the derived results in p53 target tumor suppressors (Figure S8F, Supporting Information) and genes for apoptosis (Figure S8G, Supporting Information). To further explore whether the effect of Smad1 on chemoresistance is determined by its p53 acetylation inhibition, an IC50 assay was performed in U87‐p53‐KO cells re‐expressing wild‐type p53 or R175H, as well as their 7KR mutants (Figure 8I,J). Smad1 KO increased the sensitivity to Eto in cells expressing wild‐type p53 or R175H, while this effect was not observed in WT^7KR^ or R175H^7KR^ expressing cells. The findings that cells expressing R175H and 7KR exhibited lower sensitivity to chemotherapy suggest the chemoresistance of missense mutant p53 may be due to the binding between Smad1 and p300 in mutant p53 cells, as this interaction is less easily dissociated than in wild‐type p53 cells. To investigate this hypothesis, Eto and HDACi (Trichostatin A, TSA) served as p53 acetylation inducers, and p53 acetylation and p300‐Smad1 interaction were examined. Eto and TSA induced a significant p53 acetylation both in U87 and U251 cells, while the ratio of p53 acetylation promotion in U251 was lower than in U87 cells (Figure 8K). Results also showed that Eto and TSA treatment resulted in a promotion of p53‐p300 interaction and a reduction of Smad1‐p300 binding. A notable finding was that the increase of p53‐p300 binding and consequent decrease of Smad1‐p300 binding in U87 cells was higher than that observed in U251 cells. The feasibility of driving p300‐binding and acetylation of wild‐type p53 was further confirmed in p53 KO U87 cells re‐expressing ectopic wild‐type p53 or different missense mutant p53 (Figure 8L). It indicated that the dissociation of the p300‐Smad1 complex was more pronounced in cells with wild‐type p53, as opposed to those with mutant p53. This difference in dissociation may be explained by the elevated phosphorylation of C‐terminal serine residues of Smad1 in cells re‐expressing missense mutant p53 (Figure S9, Supporting Information).^[^ ^37^ ^]^ Collectively, these observations demonstrate that the development of chemoresistance due to missense mutant p53 is closely associated with the tight binding of Smad1 and p300 and that targeting acetylation can reverse its impact.

### 2.9 A Small Molecule Targeting Smad1‐p300 Binding is an Effective GBM Suppressive Strategy

**Figure 9:**
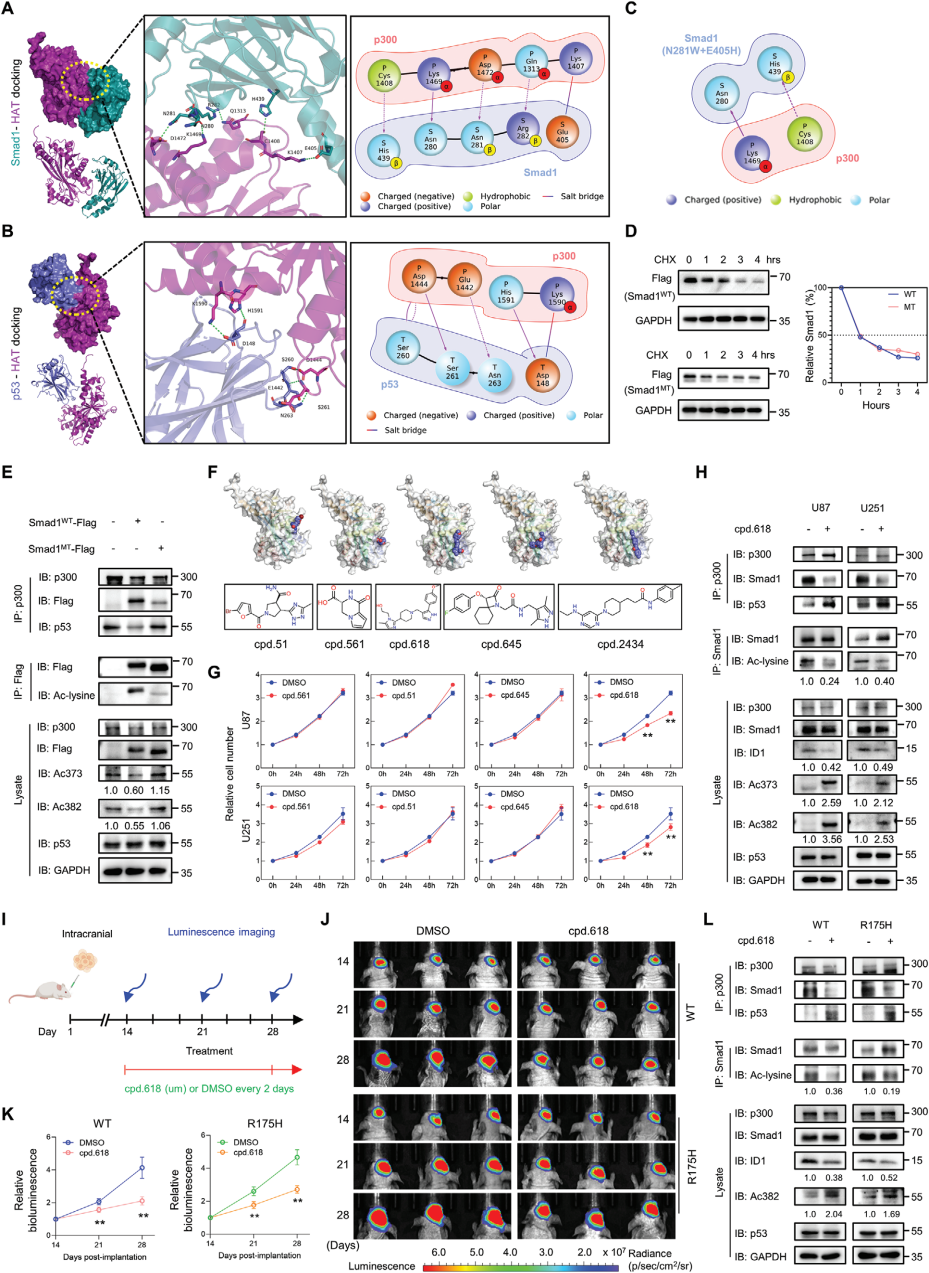
A small molecule targeting Smad1‐p300 interaction is an effective GBM suppressive strategy. A) The binding and interaction modes between p300 HAT domain (purple) and Smad1 (turquoise). The right panel shows the interactive sites and modes of action. B) The binding and interaction modes between p300 HAT domain (purple) and p53 (sky blue). The right panel shows the interactive sites and modes of action. C) Interaction mode between the combinative Smad1 mutation (N281W+E405H) and p300. The types and quantities of key interactions are reduced. D) Protein half‐life assay of wild‐type Smad1 (Smad1^WT^) and its combinative mutation (N281W+E405H; Smad1^MT^) in U87 cells. Cells were treated with Cycloheximide (CHX, 20 µg/mL) for indicated time before harvest, and relative Smad1 level was shown in the right panel. E) IP assay detecting the overall Smad1 acetylation and interaction between p300 and Smad1 or p53 using Flag and p300 antibody in U87 cells expressing Smad1^WT^ or Smad1^MT^. The relative quantifications of p53 acetylation were listed. F) Virtual screening derived five small molecular compounds with strong binding capacity to p300 at Smad1 binding sites but low binding capacity to p300 at p53 binding sites. G) Cell growth assay evaluating the growth inhibition of indicated compounds in U87 and U251 cells (*n* = 6, ^**^ *p* < 0.01). H) IP assay measuring the acetylation of Smad1 and p53, and interaction between p300 and Smad1 or p53 using p300 and Smad1 antibody in U87 and U251 cells. The cells were treated with cpd.618 (100 µM) or DMSO for 24 h before harvest. The relative quantifications of indicated proteins were listed. I) Schematic illustration of the evaluation of in vivo tumor growth inhibition of cpd.618. Mice were intracranially injected with p53 KO‐U87 cells re‐expressing p53 WT or R175H and received intraperitoneal injections of DMSO or cpd.618 (200 µM) from the 14^th^ day post‐implantation. Tumor volume was monitored at 14^th^, 21^st^ and 28^th^ days post‐implantation using luminescence imaging. J) Images of the derived intracranial tumors. K) The statistics of tumor volume (*n* = 5, ^**^ *p* < 0.01). L) IP assay measuring the acetylation of Smad1 and p53, and the interaction between p300 and Smad1 or p53 in the derived tumors. Western blot assay detecting the expression of indicated proteins in the derived tumors. The relative quantifications of indicated proteins were listed.

The strong occupancy of Smad1 MH2 at the C‐terminus of p300 suggests that disrupting the interaction between MH2 and p300 could be a promising approach for rescuing p53 tumor suppression. Therefore, we sought potential small molecule compounds or peptides that block Smad1‐p300 binding without destroying the p53‐p300 interaction. The 3D structure of proteins (Figure S10, Supporting Information) used in this study was obtained from PDB data,^[^ ^46^ ^]^ in which the C‐terminus of p300, including the ZZ domain (6DS6), the TAZ2 domain (3IO2), the histone acetyltransferase (HAT) domain (6V90), Smad1 MH2 (1KHU), and wild‐type p53 (8F2H) were used for molecular docking using HDOCK.^[^ ^47^ ^]^ Molecular docking and configuration were scored by the empirically based iterative scoring function ITScorePP, where a negative score indicates molecular binding, and the larger the absolute value, the stronger the binding ability. The top 10 conformations were scored and analyzed with a Confidence Score (SC) greater than 0.7, indicating reliable docking scores and a high likelihood of molecular binding (Table S6, Supporting Information). The HAT domain of p300 showed a higher binding possibility and strength compared to the other domains. The binding and interaction modes suggested that ZZ and TAZ2 are small fragments with less hydrogen bonding to Smad1 (Figure S11A, Supporting Information), and do not have pockets accommodating the binding of small molecules, making them unsuitable for screening targets. However, the HAT domain contains a number of binding pockets suitable for small molecule screening (**Figure** **9**A), and the specific interaction statistics are presented in Table S7 (Supporting Information). For p53‐p300 interactions, HAT contained higher binding probability and strength than the other two domains (Figure 9B; Figure S11B and Tables S8&S9, Supporting Information;). These data clarify that HAT is a common region for p53 and Smad1 recruiting p300, but specifically binding at interlocking amino acid sites. The analysis in Figure 9A addressed five hot spots responsive to Smad1 binding p300, suggesting alteration of the amino acids at these five sites may significantly impair the interaction between Smad1 and p300. To this end, Rosetta 3.13 software was applied to design and modify amino acids at the hotspots for p300‐Smad1 binding.^[^ ^48^ ^]^ The principle followed was that combinate mutations resulted in minimal p300 affinity (ΔAffinity) while affecting the structural thermal stability (ΔStability) of Smad1 as little as possible. According to Figure 9C and Table S10 (Supporting Information), the combinative mutation at Asn281(N281) and Glu405 (E405) resulted in a significant decline in p300‐affinity, while maintaining Smad1 stability. Thus, we constructed the mutant Smad1 (Smad1^MT^) carrying dual amino acid mutations (N281W+E405H) and then evaluated its stability when expressed in U87 cells. Cycloheximide (CHX) chase assay determined the half‐life of wild‐type Smad1 (Smad1^WT^) was ≈1 h based on protein synthesis inhibition, and the half‐life of Smad1^MT^ was not significantly altered (Figure 9D). However, the ability of Smad1^MT^ to bind to p300 was significantly reduced, consequently leading to a significant inhibition of acetylated Smad1. This also resulted in a loss of the ability to inhibit p53 acetylation (Figure 9E), demonstrating that disrupting the interaction between Smad1 and p300 is sufficient to rescue p53 acetylation. Next, we performed a virtual screening of small molecular compounds against the binding sites of Smad1 with the HAT domain of p300, based on 3355 small molecules from the PPI inhibitor library of Topscience Database (https://www.tsbiochem.com). Five candidate molecules were selected according to their docking scores (Table S11, Supporting Information;), the character with the strongest affinity for p300 at p300/Smad1 interactive surface, and the weakest affinity for p300 at p300/p53 binding surface (Figure 9F; Figure S12, Supporting Information). These compounds can form multiple hydrogen bonding interactions with p300, and the imidazole ring can form cation‐π stacking interactions with basic amino acids such as Lys1469 and Lys1473, which greatly determines the Smad1 binding of p300. Upon being occupied by these compounds, the interaction of Smad1 and p300 was potentially blocked. According to the cell growth assay, cpd.618 was found to be the only compound that indicated growth inhibition capacity (Figure 9G). Colony formation and cellular apoptosis assay provided additional evidence of cell proliferation inhibition and apoptosis promotion by cpd.618 (Figure S13, Supporting Information). Subsequently, the addition of cpd.618 was found to hinder the binding of Smad1‐p300 and promote the interaction of p53‐p300, resulting in an increase of p53 acetylation and a decrease of Smad1 acetylation in U87 and U251 cells (Figure 9H). Therefore, the tumor suppression of cpd.168 was further investigated in in vivo models (Figure 9I). As shown in Figure 9J,K, cpd.618 significantly suppressed the growth of tumors derived from p53 KO‐U87 cells re‐expressing wild‐type p53 (WT). This tumor inhibition was also observed in tumors derived from p53 KO‐U87 cells re‐expressing R175H mutation (R175H). Consequently, cpd.618 treatment caused a reduction in the interaction between Smad1 and p300, along with an increase in the binding of p53 to p300. This led to elevated levels of acetylated p53 and its target genes, as well as decreased levels of ID1 and acetylated Smad1 in the derived tumors. (Figure 9L; Figure S14, Supporting Information). In summary, these results indicate that cpd.618 serves as an efficient strategy for suppressing GBM by disrupting the binding of Smad1 and p300.

## 3 Discussion

**Figure 10:**
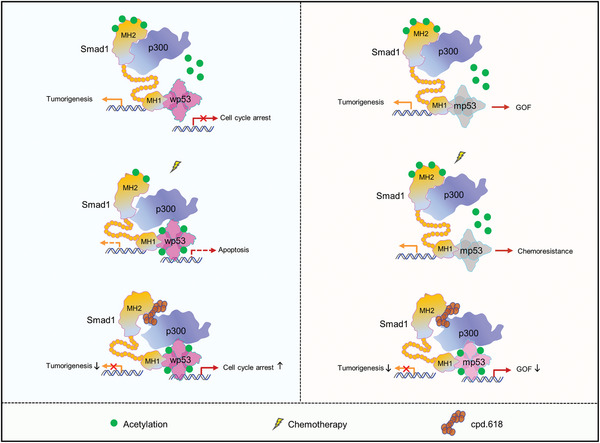
A putative working model of p300‐Smad1/p53 acetylation regulation axis in GBM. Smad1 forms a complex with p53 and p300, leading to reduced p53 acetylation and increased Smad1 acetylation, promoting tumor growth and chemoresistance in tumors with mutant p53. Disrupting the Smad1‐p300 interaction with a small molecule, cpd.618, inhibits GBM tumorigenesis and increase chemosensitivity by restoring p53 acetylation and suppressing Smad1 acetylation.

The role of acetylation in p53 activation is well‐established, but its impact on the malignancy of GBM is not well understood. A newly discovered regulatory mechanism involving p300‐Smad1/p53 acetylation sheds light on the role and regulation of p53 acetylation in GBM (**Figure** **10**). Our research documents that Smad1 acts as an onco‐protein in GBM by hijacking p300, leading to hyperacetylation of Smad1 and hypoacetylation of p53. The tight binding of Smad1 with p300 is not readily dissociated, leading to chemoresistance in tumors with missense mutant p53. p300‐mediated acetylation of Smad1 at K373 is essential for its oncogenic function, suggesting that disrupting the interaction between Smad1 and p300, rather than Smad1 and p53, could be a promising approach to inhibit GBM. A cell‐penetrating small molecule that disrupts the interaction between p300 and Smad1 has been identified, which in turn promotes p53 acetylation and function, reduces gain‐of‐function, and enhances chemosensitivity in patients with mutant p53.

p53 is the first non‐histone protein identified to be acetylated by histone acetyltransferases. p53 acetylation levels correlate with stabilization, activation in response to stress, and sequence‐specific DNA‐binding.^[^ ^49^ ^]^ Of these HATs, p300/CBP is the most well‐studied p53 acetyltransferases.^[^ ^50^, ^51^ ^]^ Recent findings identified multiple cofactors that promote the binding of p300 to p53, including ArhGAP30, BRD7, BRCA1, and WTX.^[^ ^16^, ^52^, ^53^, ^54^ ^]^ Accordingly, multiple cofactors that inhibit p300 mediated p53 acetylation have been disclosed, including MDM2, DDX24, and SET/TAF‐Iβ.^[^ ^55^, ^56^, ^57^ ^]^ This study has found that Smad1 acts as a repressive mechanism of p53/p300 binding and acetylation. The core of the p300‐Smad1/p53 regulatory relationship lies in the interlocking amino acid binding sites of Smad1 and p53 on p300 at the HAT domain. Due to its structural characteristic, Smad1 binds p53 and p300 through its first and last domain, respectively, thereby occupying the region where p300 binds to p53. The poor prognosis of patients with high levels of Smad1 can be attributed to its ability to bind to both p53 and p300, sequestering p300 from p53. This results in the oncogenic role of acetylated Smad1, and a decreased likelihood of p53 acetylation. Patients with mutated p53 and high Smad1 face an even greater challenge, as mutant p53 is less likely to be acetylated and activated due to the strong binding between Smad1 and p300 in cells with mutant p53.

It is well‐known that MDM2 plays a crucial role in mediating ubiquitination and subsequent proteasomal degradation to maintain p53 homeostasis in cells. Acetylation has been shown to extend p53 half‐life by inhibiting ubiquitination by MDM2.^[^ ^27^, ^28^ ^]^ However, the current study did not observe changes in p53 ubiquitination and half‐life in Smad1 KO or OE cells, indicating that the acetylation of p53, regulated by Smad1, does not appear to impact the relationship between p53 and MDM2. It is consistent with the findings from Wei Gu's team, which suggest that p53's transcription and tumor suppression can occur independently of its stability. This is achieved by overcoming the inhibition of its transcriptional activity by various negative regulatory binding proteins.^[^ ^58^ ^]^ Additionally, it is possible that the acetylation sites of p53 affected by Smad1 do not overlap with the ubiquitination sites mediated by MDM2.

Smad1, as well as Smad5,8 are signal transducers and transcriptional modulators that mediate BMP signals.^[^ ^59^ ^]^ In response to BMP, Smad1 is phosphorylated, translocated to the nucleus, and poised to form complexes with other transcriptional regulators. Our data showed that Smad1 is predominately expressed in the nuclei of GBM tissues and cell lines, executing onco‐functions in a BMP or TGF‐β independent manner. The current study intensifies the understanding of the interaction among Smad1, p300, and p53, and is the first report that Smad1 can be acetylated by p300. It highlights that Smad1 has a potent pro‐carcinogenic impact unrelated to p53 and is instead influenced by acetylation at K373, as demonstrated in p53‐KO GBM cells. Either wild‐type Smad1 or K373R overexpression failed to exhibit a protective effect against chemotherapy in p53‐KO GBM cells. These results identify K373 as a decisive acetylation site for the determination of the onco‐functions of Smad1 in GBM, but not associated with chemotherapy protection.

A novel finding of the present study is that missense mutant p53 is less susceptible to acetylation than wild‐type p53, even in the presence of chemical stressors. This feature is mechanically ascribed to the resistance of the Smad1‐p300 complex to dissociation. Missense mutations result in abrogation of p53 tumor suppressive function leading to a GOF that promotes cancers.^[^ ^5^ ^]^ Collective evidence from our laboratory and others indicates that acetylation destabilizes mutant p53, and contributes to structural and chemical remodeling, solubility promotion, and reactivation of mutant p53.^[^ ^15^, ^17^, ^60^, ^61^ ^]^ We propose that the GOF of missense mutant p53 comes from the hard‐to‐drive acetylation because of Smad1 occupying p300, thereby, emphasizing the decisive role of acetylation in determining tumor suppressor properties of p53 in GBM. It highlights that releasing p300 from Smad1 hijacking is a promising strategy for GBM intervention. It is well‐documented that the absence of p53 or the presence of mutant p53 can augment TGF‐β‐induced R‐Smad phosphorylation.^[^ ^62^, ^63^ ^]^ Phosphorylation of the C‐terminal serine residues of Smad1 can enhance its binding to p300/CBP and promote Smad1‐dependent transcription.^[^ ^37^ ^]^ Hence, the present discovery indicates that the enhanced phosphorylation of Smad1 in cells with p53 mutation may lead to a stronger interaction between Smad1 and p300.

The most important contribution of this study is to identify a small molecule, cpd.618, with application potential based on the above‐mentioned findings. This small molecule mechanically inhibits the interaction between p300 to Smad1 without affecting protein stability, thereby promoting the acetylation of p53. It suppresses the proliferation of tumor cells, and its pro‐apoptosis effect occurs closely accompanied by chemotherapy, which is consistent with the functional characteristics of p53 action.^[^ ^19^ ^]^ The tumor growth suppression of cpd.618 is more likely due to the inhibition of Smad1 acetylation in wild‐type p53 containing tumors. However, in tumors with missense mutant p53, its tumor inhibition may be derived from a dual effect of inhibiting Smad1 acetylation suppression and mutant p53 acetylation promotion. Critical challenges need to be addressed before this small molecule has practical value, including evaluation of serum tolerance and protection from degradation, delivery efficiency, tumor targeting, and accumulation in tumor vasculature and the microenvironment. For brain tumors, the key issue is whether therapies can effectively penetrate the blood‐brain barrier. If such is the case with cpd.618, the in vivo tumor inhibition observed in this study may be further amplified. In addition, whether cpd.618 is toxic to normal cells is still not well‐understood, and further careful verification or optimization is needed.

The present study additionally discloses the interaction between p53 and Smad1, identifying the MH1 site as essential for Smad1 binding of p53, but dispensable for p53 acetylation inhibition. The interplay between Smad1 and p53 has been documented in other cancers, demonstrating the ability to suppress tumorigenesis and induce chemoresistance by inhibiting MDM2‐mediated p53 ubiquitination and degradation in the presence of genotoxic stress.^[^ ^22^, ^23^ ^]^ DNA damage activates p53 through a phosphorylation‐acetylation cascade,^[^ ^64^ ^]^ and acetylation increases p53 protein stability.^[^ ^10^ ^]^ The binding of Smad1 to p53 observed in the present study was independent of DNA damage, and p53 stability alteration was not observed, indicating that acetylation and ubiquitination are mutually exclusive but perhaps equally impactful for p53 degradation and cellular localization.^[^ ^65^ ^]^ The interaction of Smad1, p53, and p300 in nuclei suggests that protein modifications other than acetylation are also involved in the subcellular localization. In addition, the unique environment of GBMs may also elicit specific cellular localization, which provides unique conditions for protein interactions.

Collectively, this study uncovers a novel mechanism in glioblastoma (GBM) involving Smad1 exploiting p300 to induce hypoacetylation of p53, resulting in its own acetylation and promotion of oncogenic effects. Additionally, it introduces cpd.618 as a potential anticancer agent that disrupts Smad1‐p300 interaction, facilitating p53 acetylation. This approach aims to inhibit Smad1‐driven oncogenic activities and combat mutant p53‐mediated chemoresistance in GBM.

## 4 Experimental Section

### Clinical Samples and Immunohistochemical Staining

p53, acetylated p53 (Ac‐p53), and Smad1 expression in GBM were analyzed by immunohistochemical (IHC) staining on tissue array. The tissue microarray chips contained a total of 64 samples (60 GBM and 4 normal brain tissues) and clinical data were obtained from the affiliated hospitals. All patient information was obtained and used in accordance with approved protocols reviewed by the Institutional Review Boards of the participating institutions. The clinical characteristics of the patient cohort are listed in Table S1 (Supporting Infomation). Briefly, tissue slides were incubated with primary antibodies (Supplemental Data 5, Supporting Information) and detected by Mouse and Rabbit Specific HRP/DAB (ABC) Detection IHC kit, according to the manufacturer's instructions (Abcam, Cambridge, UK). Cell nuclei were counterstained lightly with crystal violet. Normal mouse or rabbit IgG was used to confirm the IHC specificity. Target protein expression was scored semi‐quantitatively based on an established immunoreactivity scoring (IRS) system with modifications.^[^ ^66^ ^]^ Briefly, IRS was produced as a product of the multiplication of the proportion score (%) and staining intensity score (0–3). The proportion score represented the percentage of positive cells. The intensity score represented the average intensity of staining (0: no staining; 1: yellow, 2: claybank; and 3: tawny). Two blinded pathologists checked and scored the slides independently. The mean IRS core was considered the final IRS (Table S1, Supporting Information).

### Online Cancer Database Analysis

The overview of *SMAD1* RNA expression in cancers was based on TCGA RNA‐seq data derived from The Human Protein Atlas (https://www.proteinatlas.org/ENSG00000170365‐SMAD1/pathology). The alterations of *SMAD1* between various tumors and normal tissues were derived from GENEPIA (http://gepia.cancer‐pku.cn/detail.php?gene = SMAD1). Comparison of *SMAD1* transcript levels between tumor samples and normal tissues was derived from GCBI based on RNA‐seqV2 datasets (https://www.gcbi.com.cn/gcanalyze/html/generadar/search/singlegene/disease/SMAD1). A large cohort expression and survival analysis targeting *SMAD1* expression was performed using a glioma database from the GlioVis data portal (http://gliovis.bioinfo.cnio.es/).

### Cell Lines, Primary Cell Preparation, and Culture Conditions

The human glioma cell lines U87, U118, U251, T98G, A172, TG905, H4 and human embryonic kidney cell line, 293T, were obtained from the Cell Bank of Type Culture Collection of the Chinese Academy of Sciences (Shanghai, China). All cells were cultured in DMEM with 10% fetal bovine serum (FBS; Invitrogen, Carlsbad, CA). These cells were characterized by Genewiz, Inc. (China) using short tandem repeat markers and were confirmed to be mycoplasma‐free (latest tested in 2018). For patient‐derived GBM cell culture, fresh brain GBM tissues were collected after tumor resection. The derived GBM cells were seeded in a spheroid microplate (400 cells/well, Corning, NY). The spheroids were cultured with GBO medium with a minor modification (without insulin) according to a previously published report,^[^ ^67^ ^]^ changing half of the medium every 2 days. The primary spheres were cultured for 10 days to obtain enough cells for passage. The second or third passage of cells was used for subsequent experiments, including tumor xenografts, Western blot assay, and cell growth assay.

### Cell Growth and Colony Formation Assay

Cell growth was assayed using the Cell Counting Kit‐8 Kit (Biotool, Houston, TX). Each experiment was repeated at least six times. For colony formation assay, 800 cells were seeded into each well of a six‐well plate with soft agar (Agarose; Sigma) and maintained in a medium containing 10% FBS for 14 days. The colonies were fixed with methanol and stained with 0.1% crystal violet; the clones containing 50 cells were counted using an inverted microscope.

### EdU Cell Proliferation Assay

DNA replication was analyzed by the EdU incorporation method as described previously.^[^ ^68^ ^]^ Briefly, cells were cultured for 36 h in a 48‐well plate, incubated with EdU (Invitrogen) for 12 h, and fixed. The staining procedure was performed according to the manufacturer's instructions (Invitrogen, Carlsbad, CA). After staining, coverslips were mounted with Gelmount containing Hoechst 33342. The quantitative results were expressed as the percentage of EdU positive cells out of the total number of cells/nuclei.

### 3D‐Spheroid Formation and Growth Measurement

Tumor sphere formation was analyzed as described previously.^[^ ^66^ ^]^ Briefly, the growth of the 3D‐Spheroid cultured cells was monitored by a microscope with a real‐time camera (EVOS FL Auto Imaging System, Life Technologies, Carlsbad, CA, USA). For the sphere growth assay, photographs of tumor spheres were taken at the indicated time points, and the sphere diameter was measured to reflect sphere growth.

### Transwell Invasion Assay

Cell invasion assays were performed using Boyden Transwell chambers (8 mm pore size, BD Biosciences, Mountain View, CA) coated with Matrigel (40 µg, BD) in 24‐well plates. Briefly, 1 × 10^5^ indicated cells were seeded in the upper chamber, and 20% FBS medium was added in the lower chamber. After incubating for 36 h, the cells were fixed in 4% formaldehyde and stained with 0.05% crystal violet. Cells on the upper side of the filters were removed carefully, and the cells on the lower side of the filters were defined as invasive cells. The invasive cells were examined and quantified using the EVOS Cell Imaging Systems (Life Technologies, Grand Island, NY, USA). Cell invasion was expressed as the percentage of migrated cells in the total area.

### Cell Apoptosis Analysis

Flow cytometry was used to detect cell apoptosis according to the Apoptosis Detection Kit instructions (KeyGen Biotech, China). Briefly, cells were cultured for 24 h and incubated with Doxorubicin (Dox, 2 µM, Selleck, Houston, TX) for another 24 h before harvest. Cells incubated with Annexin V‐FITC/ 7‐aminoactinomycin D (7‐AAD) were immediately analyzed by a FACScan flow cytometry (Becton–Dickinson, Mountain View, CA, USA).

### Molecular Docking

To achieve an optimal small molecule that contained the dual functions of inhibiting Smad1‐p300 interaction and promoting p53 acetylation, more precise identification of the binding sites of p300 to Smad1and p53 were analyzed. The structures of selected human proteins were derived from PDB data (https://www.rcsb.org/): Smad1 (PDB: 1KHU), p53 (PDB: 8F2H), p300 ZZ domain (PDB: 6DS6), p300 TAZ2 domain (PDB: 3IO2), and p300 acetyltransferase domain (PDB: 6V90) (Figure S10, Supporting Information). Protein pretreatment was performed using UCSF Chimera to remove water molecules and irrelevant heteroatoms, retaining only the protein structure.^[^ ^69^ ^]^ Protein atomic charges were calculated using AMBER14SB, and amino acid PK values were calculated and assigned under neutral conditions (pH 7.0) using an H++3 online tool.^[^ ^70^ ^]^ Protein‐protein docking using HDOCK^[^ ^47^ ^]^ was employed to identify the specifics and differences between the binding sites of p300 to Smad1 and p53.^[^ ^47^ ^]^ Molecular docking and conformation scoring were performed using the empirically based iterative scoring function ITScorePP, where a negative score indicates molecular binding and a larger absolute value indicates stronger binding. The maximum number of output configurations for docking was set to 100, the top 10 conformations were scored, and a Confidence Score for reliability analysis. A value greater than 0.7 indicates a reliable docking score and a high likelihood of molecular binding, while a value below 0.7 indicates low credibility of docking score and a low likelihood of binding.

### Small Molecule Virtual Screening

To screen protein‐protein interaction inhibitors (PPIs) that interfere with Smad1 to p300 binding, molecular docking was performed using Autodock 4.2 suite^[^ ^71^ ^]^ to achieve candidates that tightly bind at the p300‐Smad1 binding pocket. Using the Smad1 binding site in the HDOCK docking results as the docking center, the 3D coordinates of the docking center were center_x = 41.80, center_y = 14.98, center_z = −31.76, and the docking box was set to be a square box with a 22.5 Å side length. Considering that p53 also binds to p300, to avoid interfering with the binding of p53 and p300, the binding site of p53 to p300 was used as the screening pocket to eliminate molecules with lower scores. The coordinates of the docking centers were set as follows: center_x = 18.65, center_y = 15.04, center_z = −6.61. The Spacing step was set to 0.375, the maximum limit of searching conformations was set to 10 000, and genetic algorithms were used for conformational sampling and scoring. The optimal conformations were selected by conformational ranking based on the docking scores. 3D mapping was performed using PyMOL 2.04,^[^ ^72^ ^]^ and 2D interaction analysis and counting of interaction types, distances, and numbers was performed via the academic version of Maestro (Schrödinger Release 2023‐3: Maestro, Schrödinger, LLC, New York, NY, 2023). The small molecule library selected for the virtual screening was obtained from the PPI inhibitor library in the Topscience database (https://www.tsbiochem.com/), containing 3355 small molecules.

### CRISPR/Cas9 Mediated Gene Deletion

The CRISPR/Cas9 system (Genechem) was used to establish *SMAD1* or *TP53* knockout (KO) in GBM cell lines. Briefly, cells were infected with Lenti‐Cas9 lentivirus and screened by puromycin (Sigma, St. Louis, MO). Single guide RNAs (sgRNAs) for the human *SMAD1* or *TP53* gene were designed and cloned into the GV371 plasmid. GV371 containing sgRNA was delivered into GBM cell lines expressing stable Cas9 by lentivirus. The sequence of sgRNAs and negative controls (NC) were listed in Supplemental Data 4 (Supporting Information). The Cas9/sgRNA‐mediated heterodimerization and digestion were assayed using the Knockout and Mutation Detection Kit (Genechem) according to the manufacturer's instructions. Western blot was performed to confirm the knockout of *SMAD1* or *TP53*.

### Vector Construction and Transduction

Full‐length cDNA encoding human *SMAD1, TP53, and EP300* were amplified by PCR and verified by DNA sequencing. Smad1‐Flag lentivirus was constructed by inserting the cDNA sequence into lentivirus vector H7294 (OBiO Technology, Shanghai, China) with a 3 × Flag‐tag. Wild‐type Smad1‐Flag plasmid was constructed on the GV141 vector (Genechem, Shanghai, China). Smad1 domain‐deletion and K418R mutants were constructed based on the wild‐type Smad1‐Flag plasmid. According to the acetylation predicting information (Figure 7C), Smad1 mutants containing indicated Lysine (K) converting to Arginine (R) were established based on wild‐type Smad1 plasmid. Another Smad1 mutant containing two amino acid replacements (N281W/E405H) was also constructed based on the wild‐type Smad1 vector. p53‐HA lentivirus was constructed by inserting the cDNA sequence into lentivirus vector GV348 (Genechem) with a HA‐tag. The p53 missense mutant (R175H) lentivirus was constructed based on the wild‐type p53 lentivirus. The CTD constitutive acetylation mutants (3KQ) were constructed based on p53 and R175H lentivirus with amino acid mutations to arginine (R) at K373, K381, and K382. The p53 amino acid site mutation (R273C, R248Q) vectors were constructed based on the wild‐type p53‐HA plasmid. For wild‐type p53 and missense mutant p53 re‐expressing in p53 KO‐U87 cells, the sequence for the above mentioned p53 constructs were introduced site mutations according to the sgRNA sequence (sgMut) of *TP53* (Figure 4G). p300 expression plasmid was constructed by inserting cDNA into expression vector GV712 (Genechem) with a Myc tag. The C‐terminal deletion mutant of p300 (del 1287–2414aa; Myc‐p300^CD^) was established based on the p300 expression plasmid. The small interfering RNA (siRNA) sequences were 5′ GCCUUCACAAUUCCGAGACAUTT 3′ (*EP300*) and 5′ GGAUAAAGUUCUUACUCAAUU 3′ (*SMAD1*). All siRNA transfection wasperformed using Lipofectamine 3000 (Thermo‐Fischer Scientific, Grand Island, NY, USA), according to the manufacturer's instructions.

### Transcriptional Reporter Assay

To evaluate p53 transcriptional activity, we used the reporter construct pGL4.38[luc2P/p53 RE/Hygro] (p53 RE‐pGL4.38, Promega, Madison, WI), which contained two copies of a p53 response element (p53 RE) that drives transcription of the luciferase reporter gene luc2P. GBM cells with stable Smad1 KO or OE were transiently transfected with the plasmid. pRL‐TKRenilla luciferase plasmid (Promega) served as a DNA transfection control through all experiments. Twenty‐four hours after transfection, p53 transcriptional activity was evaluated by a luciferase reporter system following our previous protocol.^[^ ^66^ ^]^ The SBE reporter kit (TGF‐β/SMAD signaling pathway) (BPS Bioscience) was transfected into cells separately. The luciferase activity was detected at 48 h after transfection.

### cDNA Microarray and Computational Analysis

lncRNA microarray was performed to detect the mRNA expression profiles of U87 cells under Smad1 depletion constructs or negative controls. Arrays were performed at OE Biotechnology Co., Ltd. (Shanghai, China) using a lncRNA expression array (Agilent Human lncRNA V5; 4*180K, Design ID:076500). Briefly, the total RNA of the indicated cells was exacted using Trizol reagent (Thermo Fisher, MA), quantified by Nanodrop 2000 spectrophotometer (Thermo Fisher), and transcribed to double‐strand cDNA. Subsequently, cDNA was synthesized into cRNA and labeled with Cyanine‐3‐CTP. The labeled cRNAs were hybridized onto the microarray and scanned by the Agilent Scanner G2505C (Agilent Technologies, Pal Alto, CA). Feature Extraction software (version10.7.1.1, Agilent Technologies) was used to analyze array images and to extract raw data. Genespring (version 14.8, Agilent Technologies) was employed to complete a basic analysis with the raw data. The raw data was normalized with the quantile algorithm. The probes with at least 1 out of 2 conditions were flagged as “P” and were chosen for further data analysis. Differentially expressed genes were then identified through fold change as well as *p* value calculated by t‐test. The threshold set for up‐ and down‐regulated genes was a fold change (FC) ≥ 1.5 and a *p* value < 0.05. Gene Set Enrichment Analysis (GSEA) was applied to determine the roles of these differentially expressed mRNAs. Finally, Hierarchical clustering was performed to display the distinguishable genes' expression pattern among samples.

### Double Immunofluorescence (IF) Staining

Double IF staining was performed following our established protocol.^[^ ^66^ ^]^ Antibodies were used to determine the indicated proteins, outlined in Supplemental Data 5 (Supporting Information). Cell nuclei were counterstained with Hoechst 33342 (Invitrogen). The sections were washed, mounted, coverslipped, and examined using the Olympus BX60 light (Olympus, Center Valley, PA, USA) or a laser scanning confocal microscope (Leica Microsystems GmbH, Mannheim, Germany). The specificity of the immunofluorescent labeling was confirmed by primary antiserum omission and normal mouse/rabbit and donkey serum controls.

### Multiplex Immunohistochemistry (mIHC)

For mIHC staining of tissue arrays, an IHC antibody complex containing Smad1, Ac‐p53, and other antibodies (not mentioned in the present study) was used in combination with an Opal 4‐color fluorescent IHC kit (PerkinElmer, USA). The tissues were incubated in primary antibodies, secondary‐HRP (Cell signaling, USA), and Opal working solution (PerkinElmer, USA). The slides were mounted with ProLong Gold Antifade Reagent containing DAPI. The slides were scanned using Vectra Polaris Imaging System (Akoya Biosciences, Marlborough, MA) and images were analyzed by Image J software (National Institutes of Health, USA). The average intensity of Smad1 and Ac‐p53 was analyzed by Image J software. The results were confirmed by two experienced pathologists blinded to the clinicopathological parameters.

### Western Blot Assay

Standard Western blot assays were used to measure protein expression. For protein stability analysis, cells were treated with cycloheximide (CHX, 20 mg mL^−1^; Sigma–Aldrich, St Louis, MO) for the indicated times to block protein synthesis. Cell lysates were performed by standard Western blot assay with the indicated antibodies to detect the target protein degradation. Antibodies used to determine the indicated proteins are shown in Supplemental Data 5 (Supporting Information).

### Immunoprecipitation (IP) Assays

IP experiments were performed to analyze the protein interactions. In brief, cells were transfected with the indicated plasmids, and whole‐cell lysates were precipitated using Protein A/G magnetic beads (Bimake, TX, USA) with the indicated antibodies. Precipitated products were analyzed by Western blot using the indicated antibodies. Antibodies used in IP and subsequent Western blots are shown in Supplemental Data 5 (Supporting Information).

### Reverse‐Transcription Quantitative PCR (RT‐qPCR)

Total RNA was extracted using TRIzol reagent (Thermo‐Fischer Scientific) according to the manufacturer's instructions. cDNA was synthesized with the M‐MLV Reverse Transcriptase Kit (Promega). PCR analyses were conducted to quantify mRNA expression using Real SYBR Mixture (CoWin Bioscience, China) on a QuantStudio 12K Flex Real‐Time PCR System (Applied BioSystems, Foster City, CA, USA). *GAPDH* severed as an internal control. The specific oligonucleotide primer pairs are listed in Supplemental Data 6 (Supporting Information).

### ChIP and ChIP‐qPCR Assays

The ChIP assay was performed using the SimpleChIP Enzymatic Chromatin IP Kit (Cell Signaling) according to the manufacturer's instructions. Briefly, 2 × 10^7^ cells and 5 ug anti‐p53 antibody or anti‐Smad1 antibody were used for each ChIP. The mouse IgG and rabbit anti‐Histone H3 antibodies were applied as negative control and positive control, respectively. DNA digestion was performed using micrococcal nuclease combined with a DNA disruption apparatus. The disruption effect was identified by DNA electrophoresis. After the reversal of cross‐links and DNA purification, the samples were used for quantitative real‐time PCR with specific primers targeting promoters (Supplemental Data 7, Supporting Information). The ID1 promoter primer was purchased from Cell Signaling Technology (89001). ChIP efficiency was expressed according to our previously established method.^[^ ^66^ ^]^

### Animal Experiments

Four weeks‐old female nude mice were obtained from the Shanghai Animal Center, Chinese Academy of Sciences, and maintained under specific pathogen‐free conditions. All mice were randomly assigned. Briefly, U87 cells with SMAD1 KO or Control (5 × 10^5^ cells per injection) were stereotactically implanted into the right hemisphere of individual mouse brains. Tumor formation was observed by magnetic resonance imaging (MRI, Siemens) 28 days after implantation. Twenty‐four hours before the end of the experiments, mice received an intraperitoneal injection of EdU to examine cell proliferation. The mice were sacrificed with an overdose of anesthetic. Whole brains were removed, paraffin‐embedded, sectioned, and stained with hematoxylin and eosin (H&E) or IF stained with EdU according to the manufacturer's instructions (Invitrogen). Images were captured using a laser scanning confocal microscope. To examine the tumorigenicity of GBM cells, the second or third passage of 3D‐cultured spheres was dissociated and counted. A total of 5 × 10^6^ cells were injected subcutaneously into the flank of each nude mouse. Tumor initiation and growth were monitored every 3 days. The glioma‐bearing mice were euthanized 28 days after the injections. The flank xenografted tumors were removed and volume was measured. For intracranial models, p53‐depleted U87 cells expressing the firefly luciferase gene were infected with wild‐type p53 or mutant p53‐overexpressing lentivirus. A total of 5 × 10^5^ cells were stereotactically implanted into the brain of individual mice. Tumor growth was monitored via bioluminescent imaging using the IVIS Spectrum system and quantified by Living Image Software. All animal care and handling procedures were performed in accordance with the National Institutes of Health's Guide for the Care and Use of Laboratory Animals. All procedures were approved by the Institutional Review Board of Nanjing Medical University (No. (2020)353). All animal experiments were performed by two blinded technicians.

### Statistical Analysis

Data are presented as mean ± SD. The difference between groups was performed using a two‐tailed Student's t‐test, the Kaplan–Meier method with log‐rank test, or *χ* ^2^‐test Spearman's rank correlation analysis. An F test was used to test variance equality. *p* < 0.05 was considered statistically significant. SPSS 16.0 package (IBM) and Graphpad prism 8.0 software (GraphPad Software) were used for all statistical analyses and data graphing, respectively.

### Ethics Statement

The study was approved by the Institutional Ethics Committee of The Affiliated Wuxi People's Hospital of Nanjing Medical University.

## Conflict of Interest

The authors declare no conflict of interest.

## Supporting information



Supporting Information

Supplemental Tables

Supporting Information

## Data Availability

The data that support the findings of this study are available in the supplementary material of this article.
