# Supplementary material for: Smad1 Promotes Tumorigenicity and Chemoresistance of Glioblastoma by Sequestering p300 From p53
Source: Adv Sci (Weinh). 2024 Dec 4;12(4):2402258. doi: 10.1002/advs.202402258 (PMC11789598; doi:10.1002/advs.202402258)
Supplement: Supplementary file 1 — Supporting Information [file ADVS-12-2402258-s003.docx]

**Smad1 Promotes Tumorigenicity and Chemoresistance of Glioblastoma by Sequestering p300 from p53**

Lingli Gong^1,2$^, Daxing Xu^1,2$^, Kaixiang Ni^4$^, Jie Li^1,2^, Wei Mao^1,2^, Bo Zhang^1,3^, Zhening Pu^1,3^, Xiangming Fang^5^, Ying Yin^1,2^, Li Ji^1,2^, Jingjing Wang^1,2^, Yaling Hu^1,2^, Jiao Meng^1,2^, Rui Zhang^4^, Jiantong Jiao^4*^, Jian Zou^1,2*^

^1.^Department of Laboratory Medicine, The Affiliated Wuxi People's Hospital of Nanjing Medical University, Wuxi People's Hospital, Wuxi Medical Center, Nanjing Medical University, Wuxi, Jiangsu, 214023, China

^2.^Wuxi Medical Center, Nanjing Medical University, Wuxi, Jiangsu, 214023, China

^3.^Center of Clinical Research, The Affiliated Wuxi People's Hospital of Nanjing Medical University, Wuxi, Jiangsu, 214023, China

^4.^Department of Neurosurgery, The Affiliated Wuxi People's Hospital of Nanjing Medical University, Wuxi, Jiangsu, 214023, China

^5.^Department of Radiology, The Affiliated Wuxi People's Hospital of Nanjing Medical University, Wuxi, Jiangsu, 214023, China.

^$^ Equal contributors

Corresponding to: **Jian Zou (lead contact)**, Department of Laboratory Medicine, The Affiliated Wuxi People's Hospital of Nanjing Medical University, 299 Qingyang Road, Wuxi, Jiangsu, 214023, China, Tel: 86-510-85350340, E-mail: zoujan@njmu.edu.cn; **Jiantong Jiao**, Department of Neurosurgery, The Affiliated Wuxi People's Hospital of Nanjing Medical University, 299 Qingyang Road, Wuxi, Jiangsu, 214023, China, Tel: 86-510-85351051, E-mail: jiaojiantong@njmu.edu.cn.

**Supplementary Figures**

**
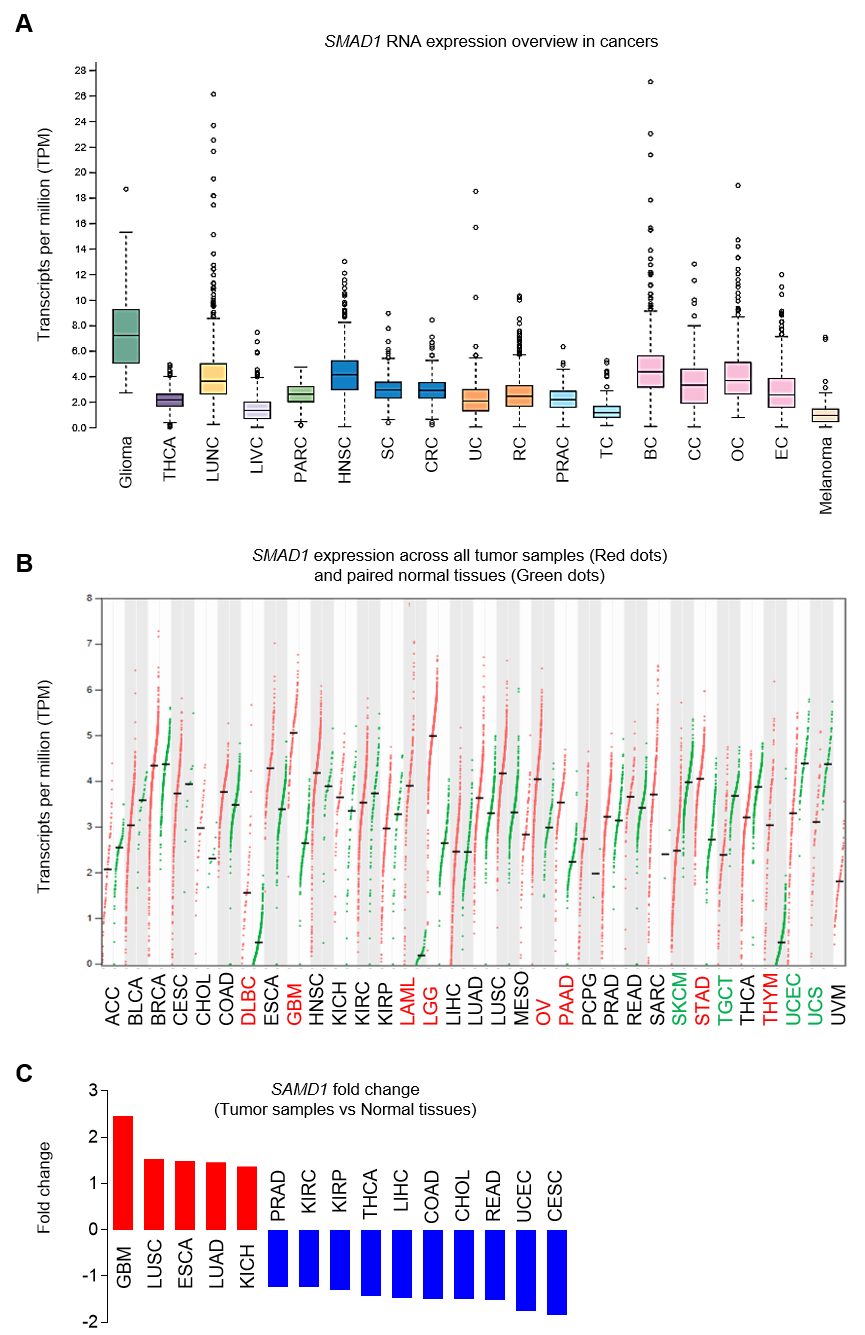
**

**Figure S1.** *SMAD1* is overexpressed in glioblastoma (GBM). A) RNA expression overview of TCGA RNA-seq data derived from The Human Protein Atlas shows gliomas have the highest *SMAD1* expression among cancers. B) Dot plot from GENEPIA shows *SMAD1* expression profile across all tumor samples (Red dots) and paired normal tissues (Green dots). Data includes TCGA normal and GTEx data. Cancers with higher *SMAD1* are shown in red and those with lower *SMAD1* are shown in green. ANOVA, Log2FC > 1, q value < 0.01.C) Comparison of *SMAD1* transcript levels between tumor samples and normal tissues. Results were derived from GCBI based on RNA-seqV2 datasets. ANOVA, Log2FC > 1, *p* < 0.05.

**
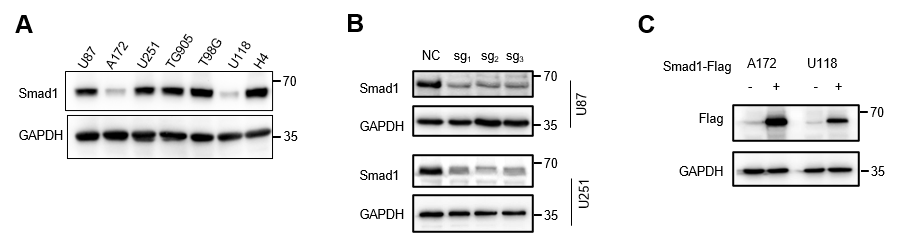
**

**Figure S2.** Verification of CRISPR/Cas9 mediated *SMAD1* knockout and overexpression construction. A) Western blot analysis showing the expression of Smad1 in various glioma cell lines. B) Western blot analysis of Smad1 expression in U87 and U251 cells with CRISPR/Cas9-mediated knockout (KO). GAPDH served as a loading control. C) Western blot analysis of ectopic Smad1 expression in A172 and U118 cells using anti-Flag antibody. GAPDH served as a loading control.


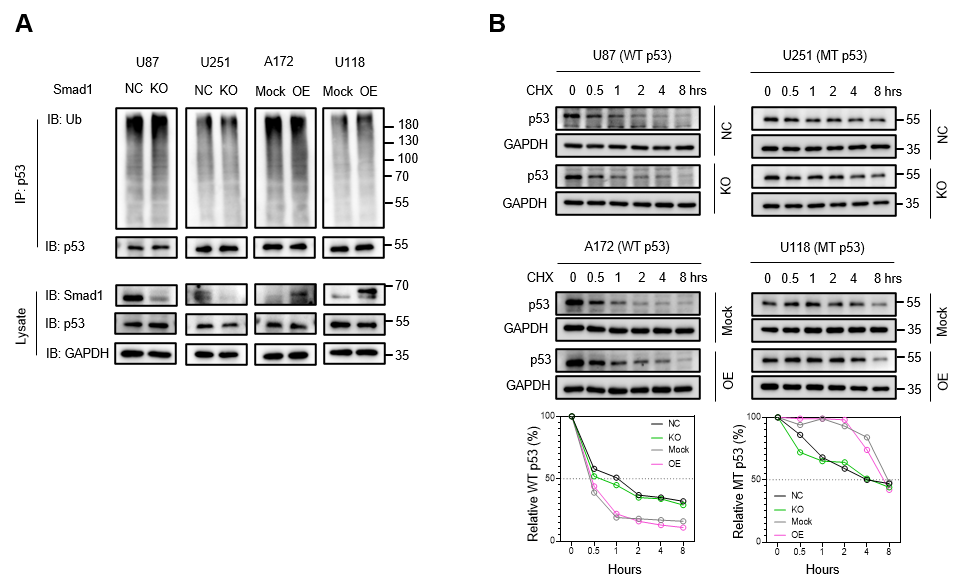


**Figure S3.** The ubiquitination and stability of p53 is not affected by Smad1 in GBM cells. A) Ubiquitination assay of p53 in indicated GBM cells. Cells were incubated with MG132 (20 μM) for 4 hours before harvest. Ubiquitin (Ub)-binding p53 was immunoprecipitated (IP) by p53 antibody and detected by Ub antibody. Target proteins in the total lysate were detected using indicated antibodies. B) Western blot analysis detecting p53 expression in indicated cells treated with Cycloheximide (CHX; 20 μg/mL) for the indicated times. The trend of p53 alterations were shown in the lower panel.


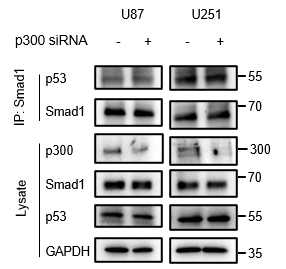


**Figure S4.** p300 knockdown fails to affect the interaction of p53 and Smad1. IP assay showing the interaction between p53 and Smad1 in U87 and U251 cells transfected with p300 siRNA using Smad1 antibody. GAPDH served as a loading control.


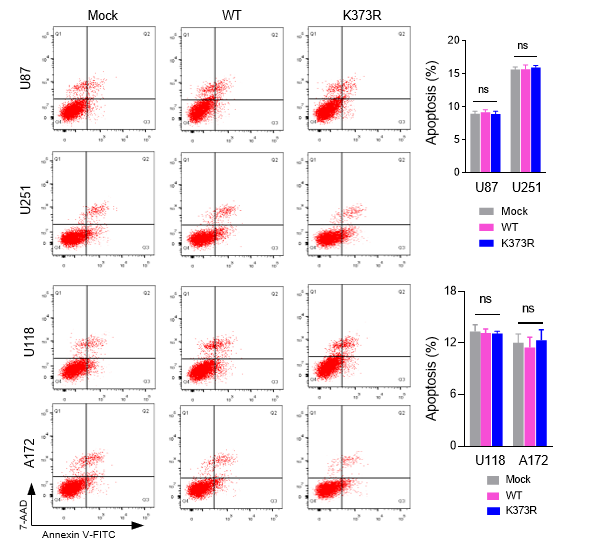


**Figure S5.** Apoptosis assay of p53-KO cells overexpressing indicated Smad1 constructs. Cells were treated with Dox (2 μM) for 24 h and apoptotic cells were analyzed by FACS.


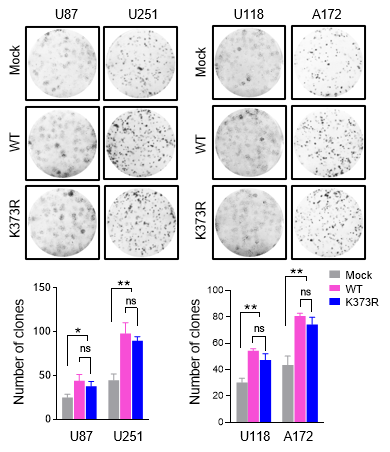


**Figure S6.** Colony formation assay of GBM cells overexpressing indicated Smad1 constructs (n = 3, ***p* < 0.01).

**
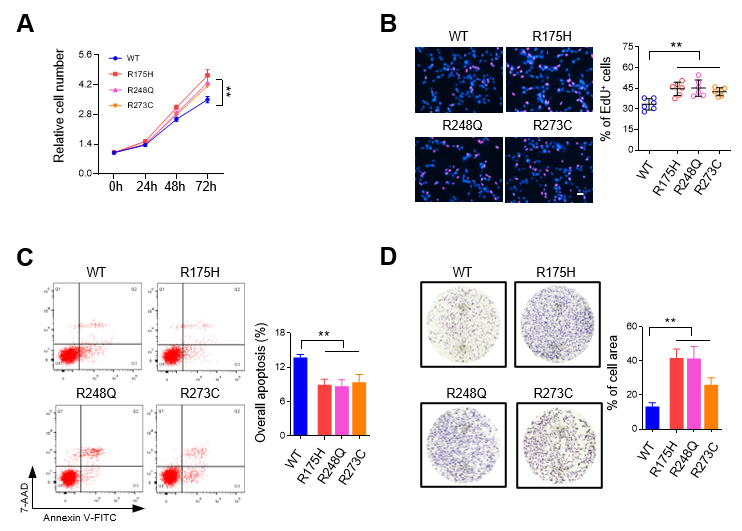
**

**Figure S7.** Missense mutated p53 promotes cell growth, proliferation, invasion, and resistance to chemotherapy in GBM cells. A) Cell growth assay of p53 KO-U87 cells expressing indicated p53 mutants (n = 6, ***p* < 0.01). B) EdU-labeling assay of p53 KO-U87 cells expressing indicated p53 mutants (n = 6, ***p* < 0.01). C) Apoptosis assay of p53 KO-U87 cells expressing indicated p53 mutants. Cells were treated with Dox (2 μM) for 24 h and apoptotic cells were analyzed by FACS (n = 3, ***p* < 0.01). D) Transwell invasion assay of p53 KO-U87 cells expressing indicated p53 mutants (n = 3, ***p* < 0.01).

**
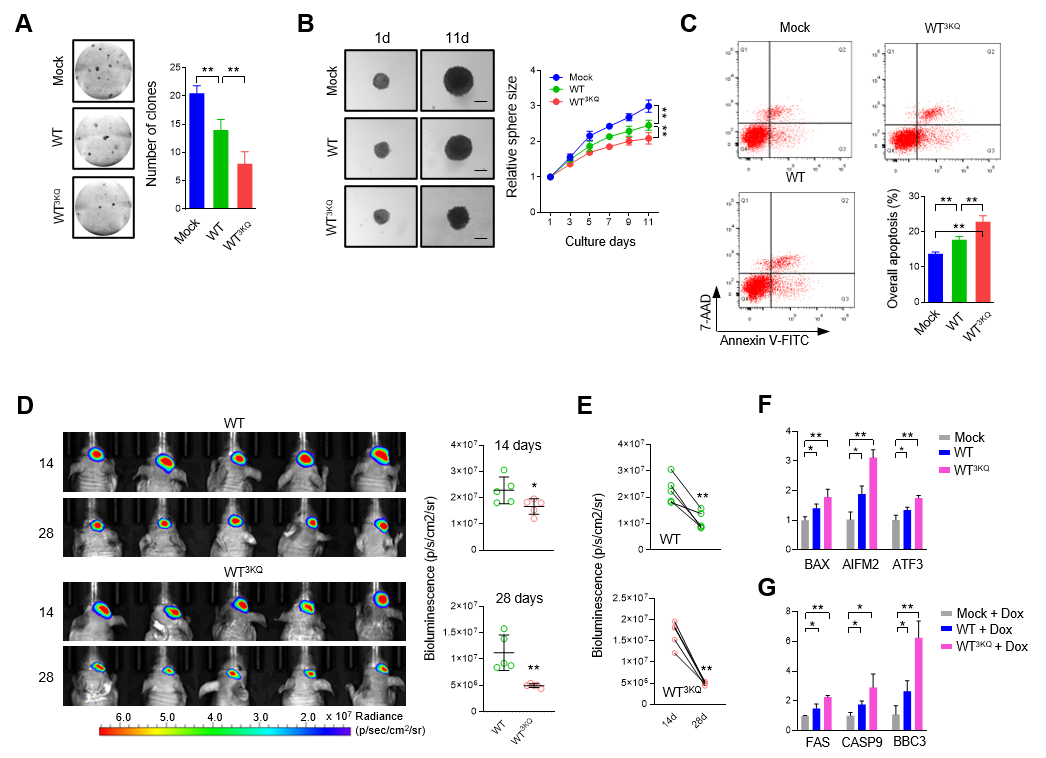
**

**Figure S8.** Acetylation enhances the inhibition of p53 in tumorigenesis and resistance to chemotherapy in GBM cells. A) Colony formation assay of p53 KO-U87 cells expressing indicated p53 constructs (n = 4, ***p* < 0.01). B) 3D spheroids assay of p53 KO-U87 cells expressing indicated p53 constructs (n = 5, ***p* < 0.01). C) Apoptosis assay of p53 KO-U87 cells expressing indicated p53 constructs. Cells were treated with Dox (2 μM) for 24 h and apoptotic cells were analyzed by FACS (n = 3, ***p* < 0.01). D) Images of the derived intracranial tumors and the statistical results of tumor volume (n = 5, **p* < 0.05, ***p* < 0.01). E) The statistical results of tumor volume before and after TMZ treatment (n=5, ***p* < 0.01). F) qRT-PCR assay measuring the indicated genes in p53 KO-U87 cells expressing indicated p53 constructs (n = 3, **p* < 0.05, ***p* < 0.01). G) qRT-PCR assay measuring the indicated genes in p53 KO-U87 cells expressing indicated p53 constructs. Cells were treated with Dox (2 μM) for 24 h before harvest (n = 3, **p* < 0.05, ***p* < 0.01).

**
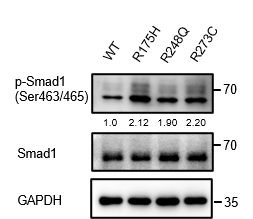
**

**Figure S9.** The phosphorylation of Smad1 is upregulated in cells harboring missense mutant p53. Western blotting assay indicating Smad1 phosphorylation was increased in p53 KO-U87 cells expressing indicated p53 mutants. The relative quantification of Smad1 phosphorylation was listed.


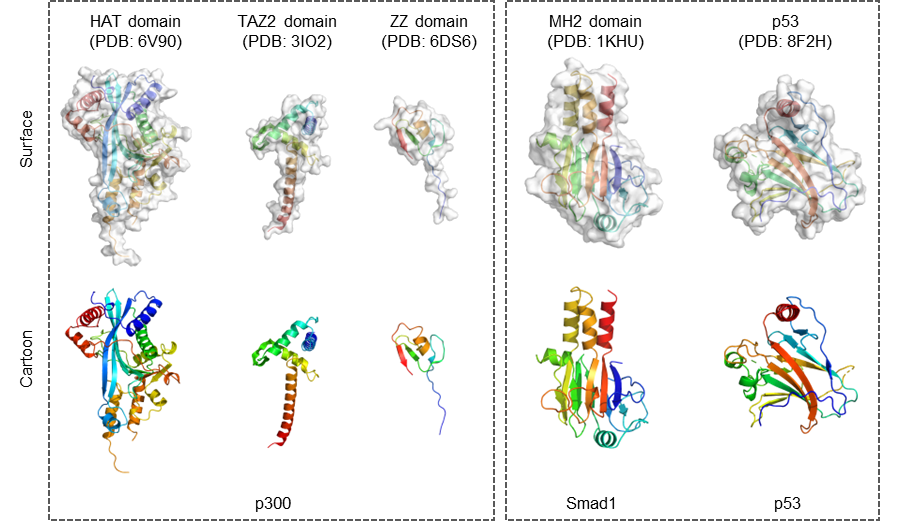


**Figure S10.** 3D structure of p300 functional domains, Smad1 and p53 used in protein-protein docking by HDOCK.


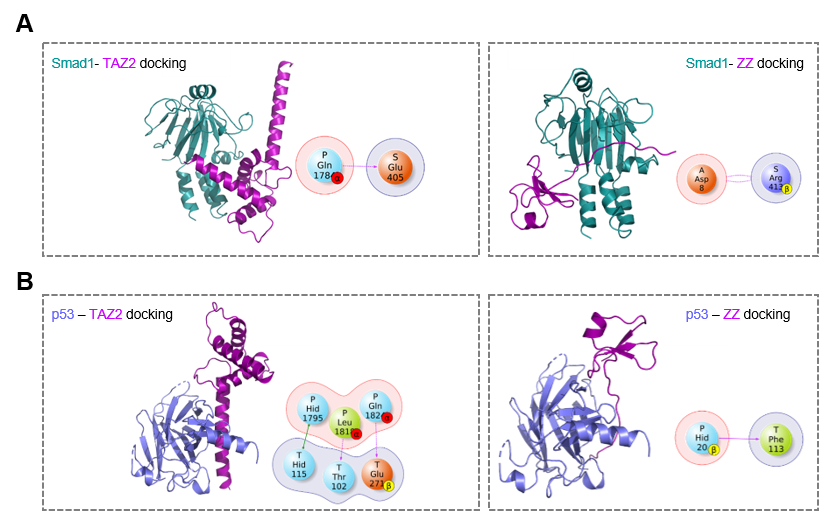


**Figure S11.** The interaction modes of p300 TAZ2 and ZZ domain with Smad1 (A) or p53 (B).


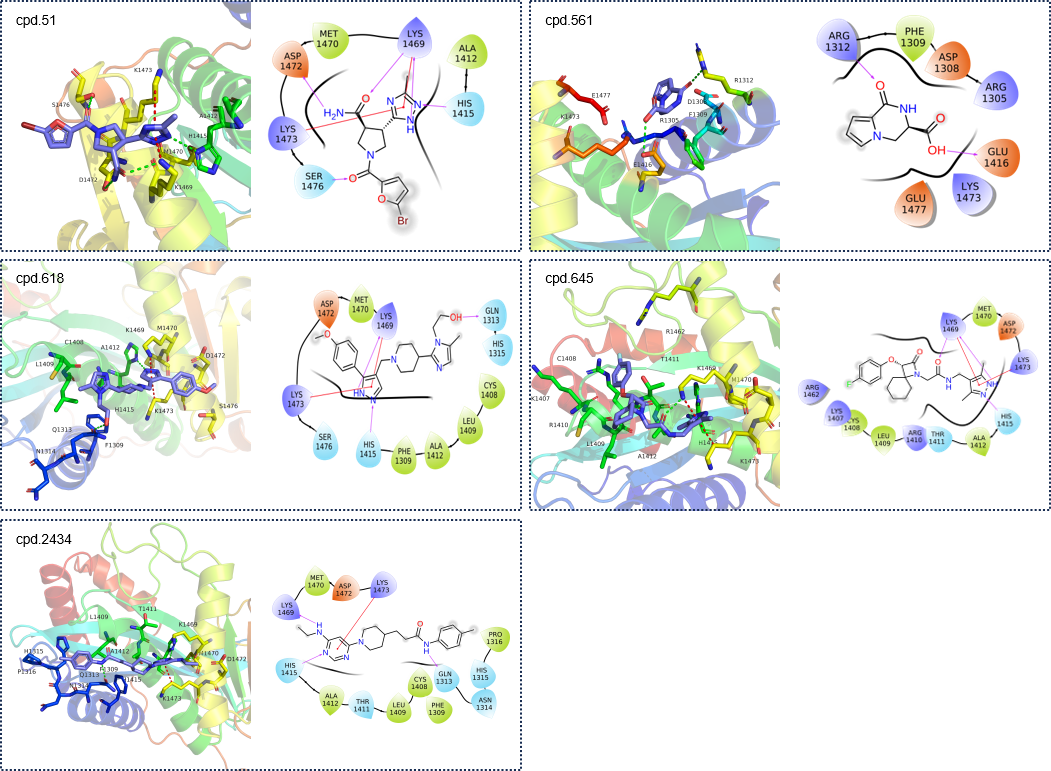


**Figure S12.** p300 (Smad1 binding site) and five candidate molecules binding modes. Interactions were indicated by dashed lines in all 3D plots, hydrogen bonding was indicated by green dashed lines, and cation-π stacking was indicated by red dashed lines.2D plots were indicated by purple arrows for hydrogen bonding, and cation-π stacking was indicated by red straight lines. For amino acid, red were acidic amino acids, green were hydrophobic amino acids, cyan were polar amino acids, and blue were basic amino.


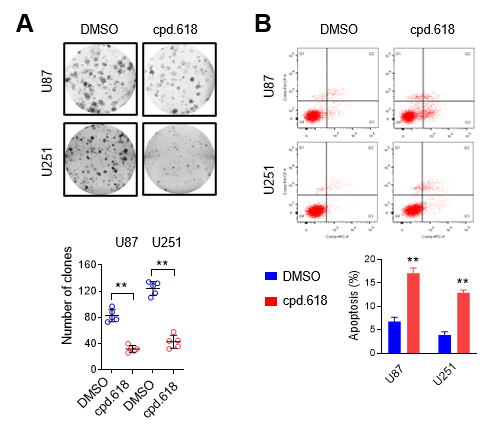


**Figure S13.** cpd.618 inhibits colony formation and promotes apoptosis of GBM cells. A) Colony formation assay of GBM cells with continuous treatment of cpd.618 (100 μM) or DMSO for 10 days. The bar graph (lower panel) showing the statistical results (n = 5, ***p* < 0.01). B) Apoptosis assay of GBM cells treated with cpd.618 (100 μM) or DMSO for 24 h (n = 3, ***p* < 0.01).


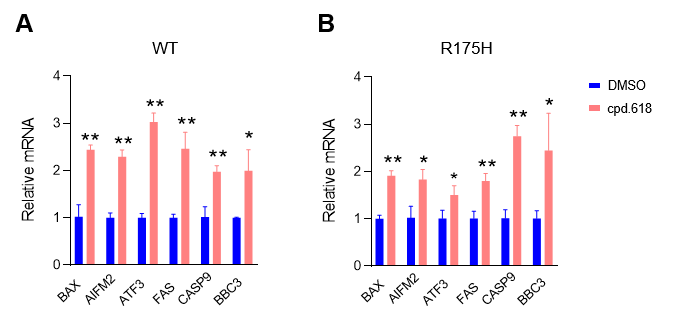


**Figure S14.** cpd.618 promotes the transcription of both wild-type and mutant p53 target genes.

qRT-PCR assay was used to measure the expression of p53-targeted genes in the derived tumors. (n = 3, **p* < 0.05, ***p* < 0.01).

**Supplemental Data**

**Supplemental Data 1.** **Association between Smad1 expression and information of patients with GBM.**

| Status | | Smad1 expression^#^, n | | Total | *p* value* |
| --- | --- | --- | --- | --- | --- |
|  |  | LOW | HIGH | n |  |
| Gender | Male | 14 | 17 | 31 | 0.6058 |
|  | Female | 16 | 13 | 29 |  |
| Age | <55 yrs | 7 | 10 | 17 | 0.5675 |
|  | ≥55 yrs | 23 | 20 | 43 |  |
| Survival^$^ | ≤1 yrs | 6 | 15 | 21 | 0.0524 |
|  | 1-3 yrs | 12 | 10 | 22 |  |
|  | >3 yrs | 9 | 4 | 13 |  |

**p* values were analyzed by Chi-square test.

# According to the immunoreactive scores (IRS) from IHC of GBM tissue array: the cutoff between LOW and HIGH was set at the median IRS of GBM tissues.

$ The total number of patients used in survival analysis was 56, those without survival information were excluded.

**Supplemental Data 2. Association between p53 expression and information of patients with GBM.**

| Status | | p53 expression^#^, n | | Total | *p* value* |
| --- | --- | --- | --- | --- | --- |
|  |  | LOW | HIGH | n |  |
| Gender | Male | 12 | 19 | 31 | 0.0705 |
|  | Female | 18 | 11 | 29 |  |
| Age | <55 yrs | 9 | 8 | 17 | 0.7745 |
|  | ≥55 yrs | 21 | 22 | 43 |  |
| Survival^$^ | ≤1 yrs | 11 | 10 | 21 | 0.6396 |
|  | 1-3 yrs | 14 | 12 | 26 |  |
|  | >3 yrs | 5 | 8 | 13 |  |

**p* values were analyzed by Chi-square test.

# According to the immunoreactive scores (IRS) from IHC of GBM tissue array: the cutoff between LOW and HIGH was set at the median IRS of GBM tissues.

$ The total number of patients used in survival analysis was 56, those without survival information were excluded.

**Supplemental Data 3. Association between Ac-p53 expression and information of patients with GBM.**

| Status | | p53 expression^#^, n | | Total | *p* value* |
| --- | --- | --- | --- | --- | --- |
|  |  | LOW | HIGH | n |  |
| Gender | Male | 16 | 15 | 31 | 0.7961 |
|  | Female | 14 | 15 | 29 |  |
| Age | <55 yrs | 9 | 8 | 17 | 0.7745 |
|  | ≥55 yrs | 21 | 22 | 43 |  |
| Survival^$^ | ≤1 yrs | 11 | 10 | 21 | 0.2744 |
|  | 1-3 yrs | 15 | 11 | 26 |  |
|  | >3 yrs | 4 | 9 | 13 |  |

**p* values were analyzed by Chi-square test.

# According to the immunoreactive scores (IRS) from IHC of GBM tissue array: the cutoff between LOW and HIGH was set at the median IRS of GBM tissues.

$ The total number of patients used in survival analysis was 56, those without survival information were excluded.

**Supplemental Data 4. Sequence of sgRNA targeting *SMAD1.***

| **GENE** | **sgRNA** | **Sequence** |
| --- | --- | --- |
| *SMAD1* | Sg1 | TTAGCTCAGTTCCGTAACTT |
|  | Sg2 | CTATGAGCTCAACAATCGTG |
|  | Sg3 | AAACGGTTCTTATTGTTGGA |
|  | NC | CGCTTCCGCGGCCCGTTCAA |
| *TP53* | sg | CCATTGTTCAATATCGTCCG |
|  | NC | CGCTTCCGCGGCCCGTTCAA |

**Supplemental Data 5. List of antibodies.**

| **Antigen** | **Primary Antibody** | **Dilution** |
| --- | --- | --- |
| **Smad1** | Abnova; H00004086; mouse monoclonal | 1:300 for IHC/mIHC; 1:1,000 for WB |
| **Smad1** | ABCAM; ab33902; rabbit monoclonal | 1:400 for IF |
| **Smad1** | Cell Signaling; 6944; rabbit monoclonal | 1:1000 for WB; 1:100 for IP |
| **p53** | Sigma; P5813; mouse monoclonal | 1:1000 for WB; 1:2000 for IHC/mIHC |
| **p53** | Abcam; ab1101; mouse monoclonal | 1:100 for ChIP |
| **p53（acetyl K373）** | ABCAM; ab62376; rabbit monoclonal | 1:1000 for WB; 1:2000 for IHC/mIHC |
| **p53（acetyl K382）** | ABCAM; ab75754; rabbit monoclonal | 1:1000 for WB |
| **p300** | ABCAM; ab10485; rabbit polyclonal | 1:5000 for WB |
| **p300** | ABCAM; ab54984; mouse monoclonal | 1:200 for IF |
| **Acetylated-Lysine** | ABCAM; ab21623; rabbit polyclonal | 1:1000 for WB |
| **ID1** | SANTA CRUZ; sc-488; rabbit polyclonal | 1:2000 for WB |
| **Nestin** | Millipore; MAB5326; mouse monoclonal | 1:100 for IF |
| **GAPDH** | ThermoFisher; MA5-15738-1MG; mouse monoclonal | 1:2000 for WB |
| **β-actin** | Thermo Fisher; MA5-15739-1MG; mouse monoclonal | 1:1000 for WB |
| **β-tublin** | Thermo Fisher; MA5-16308-1MG; mouse monoclonal | 1:2000 for WB |
| **Normal Rabbit IgG** | Cell Signaling; 2729 | 1:100 for IP or ChIP; 1:500 for IHC or IF |
| **Normal Mouse IgG** | ABCAM; ab188776 | 1:100 for IP; 1:500 for IF |
| **Flag-tag** | Abmart; M20008; mouse monoclonal | 1:5000 for WB; 1:100 for IP |
| **GFP-tag** | Cell Signaling; 2956; rabbit monoclonal | 1:1000 for WB |
| **HA-tag** | ABCAM; ab9110; rabbit polyclonal | 1:5000 for WB; 1:200 for IP |
| **Myc-tag** | Abmart; M20002; mouse monoclonal | 1:2000 for IF; 1:5000 for WB; 1:200 for IP |

**Supplemental Data 6. Primers used for qPCR.**

| **Gene** | **Forward** | **Reverse** |
| --- | --- | --- |
| *BAX* | CCCGAGAGGTCTTTTTCCGAG | CCAGCCCATGATGGTTCTGAT |
| *ATF3* | CGCTGGAATCAGTCACTGTCAG | CTTGTTTCGGCACTTTGCAGCTG |
| *AIFM2* | GACTCCTTCCACCACAATGTGG | CAGCACCATCTGGTTCTTCAGG |
| *FAS* | GGACCCTCCTACCTCTGGTT | CTCCTTCCCTTCTTGGCAGG |
| *CASP9* | TTTGAGGACCTTCGACCAGC | TTAGCGACCCTAAGCAGGAG |
| *BBC3* | TGAAATTTGGCATGGGGTCTG | GCCCTCTACGGGCTCC |
| *TP53* | CCTCAGCATCTTATCCGAGTGG | TGGATGGTGGTACAGTCAGAGC |
| *SMAD1* | TTGGCACAGTCTGTGAACCATGG | GTAACATCCTGGCGGTGGTATTC |
| *GAPDH* | CAACTTTGGTATCGTGGAAGGACTC | AGGGATGATGTTCTGGAGAGCC |

**Supplemental Data 7. Primers used for qPCR of ChIP.**

| **Gene** | **Forward** | **Reverse** |
| --- | --- | --- |
| *BAX* | AGCCTGGGCGTGGGCTATATT | CCAGGCAGGACGTTATAGATGACT |
| *ATF3* | CCACACCACAGACTAACGCT | GCGTGGTCATTTTCTGGAGC |
| *AIFM2* | TGGCCATGCCCAATCTTTCTTA | GACAGATATTAAACACACCACCAAA |
| *FAS* | TCCAGAAACGTCTGTGAGCC | GGGAAGTTGGGGAGGTCTTG |
| *CASP9* | GCTCCGTGCTCCAGTTAGTT | TCGCCTTCAGTGCGATAGAC |
| *BBC3* | CCCTGTGCCTATCAGCAAGT | AACAACCCTACCGAACAGGC |
