# Supplementary material for: Smad1 Promotes Tumorigenicity and Chemoresistance of Glioblastoma by Sequestering p300 From p53
Source: Adv Sci (Weinh). 2024 Dec 4;12(4):2402258. doi: 10.1002/advs.202402258 (PMC11789598; doi:10.1002/advs.202402258)

ADVANCED SCIENCE
Open Access

Supporting Information

for *Adv. Sci.*, DOI 10.1002/advs.202402258

Smad1 Promotes Tumorigenicity and Chemoresistance of Glioblastoma by Sequestering p300 From p53

Lingli Gong, Daxing Xu, Kaixiang Ni, Jie Li, Wei Mao, Bo Zhang, Zhening Pu, Xiangming Fang, Ying Yin, Li Ji, Jingjing Wang, Yaling Hu, Jiao Meng, Rui Zhang, Jiantong Jiao and Jian Zou**

Figure 1F

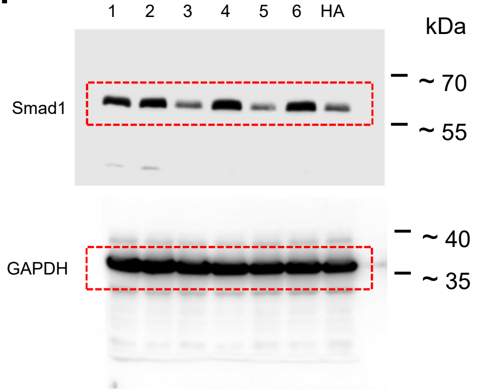

Figure 4A

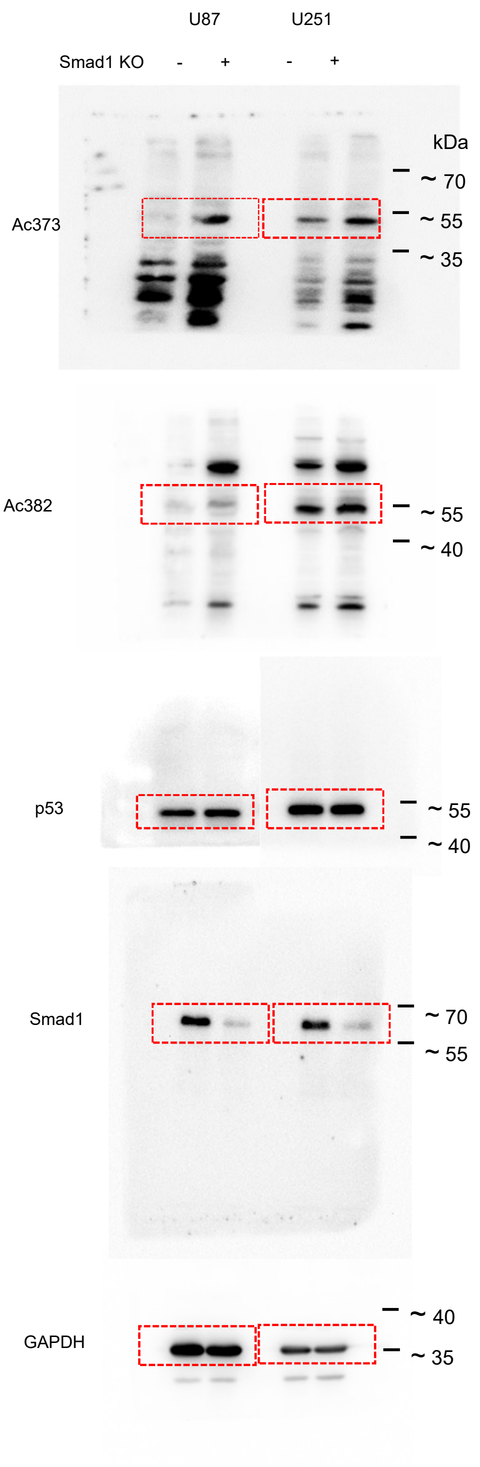

Figure 4B

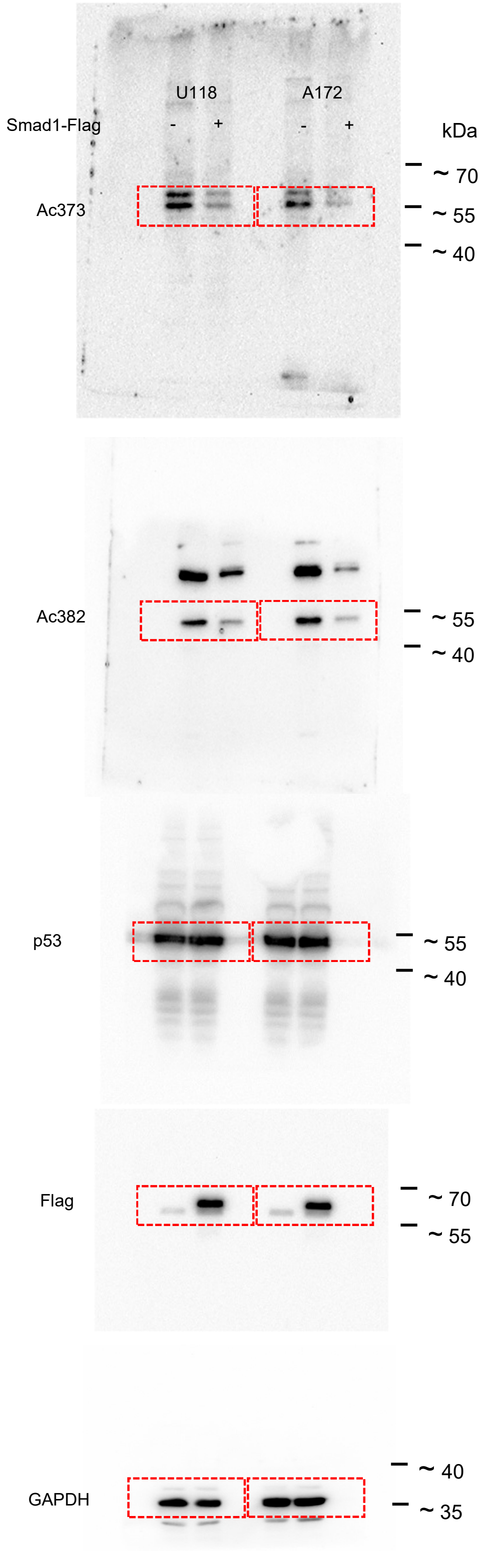

Figure 4C

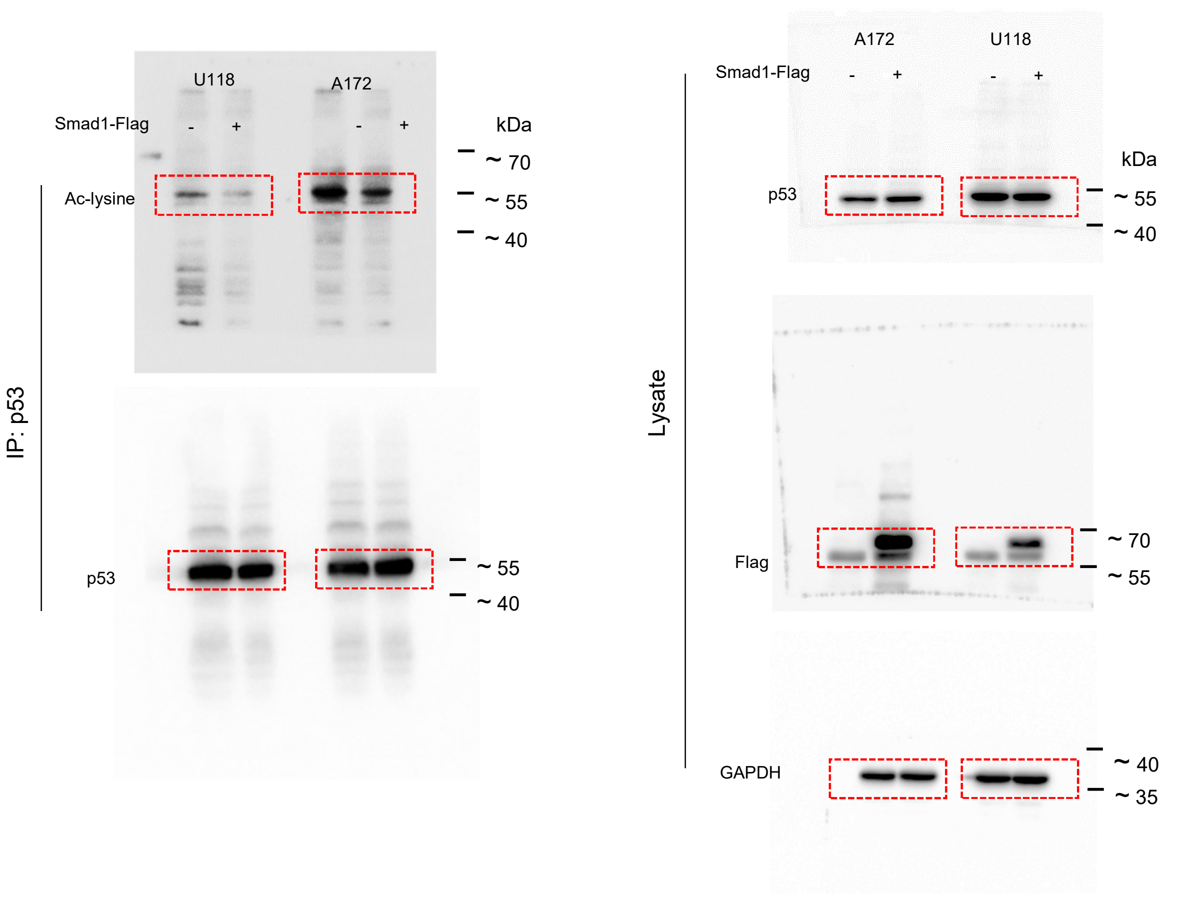

Figure 4D

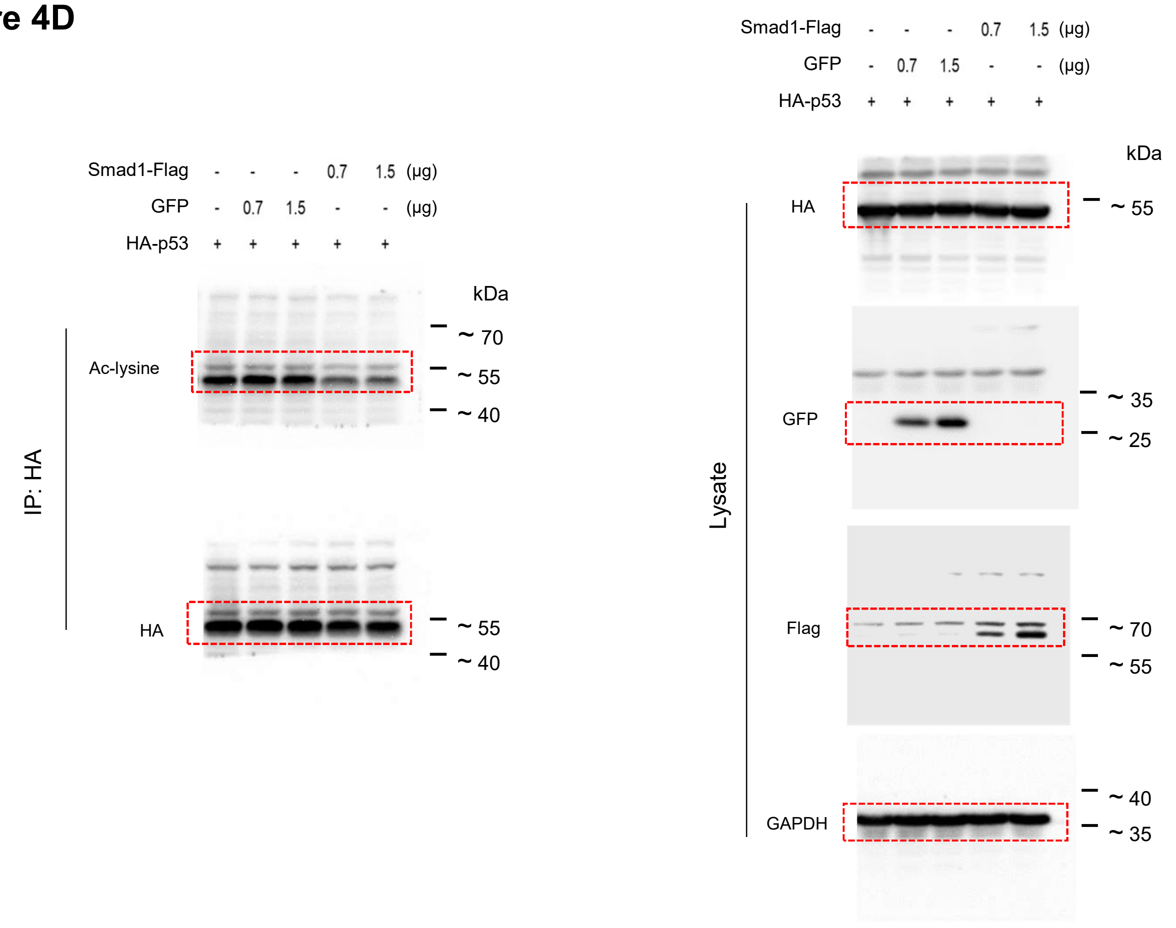

Figure 4E

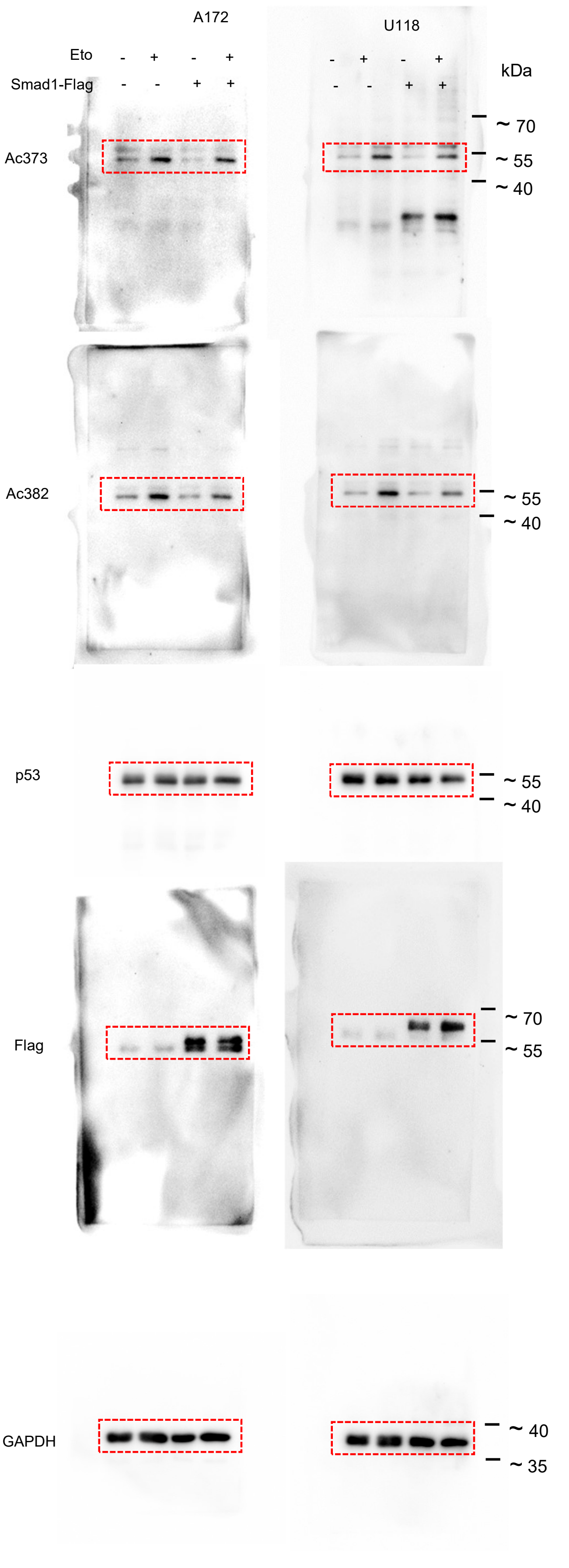

Figure 4F

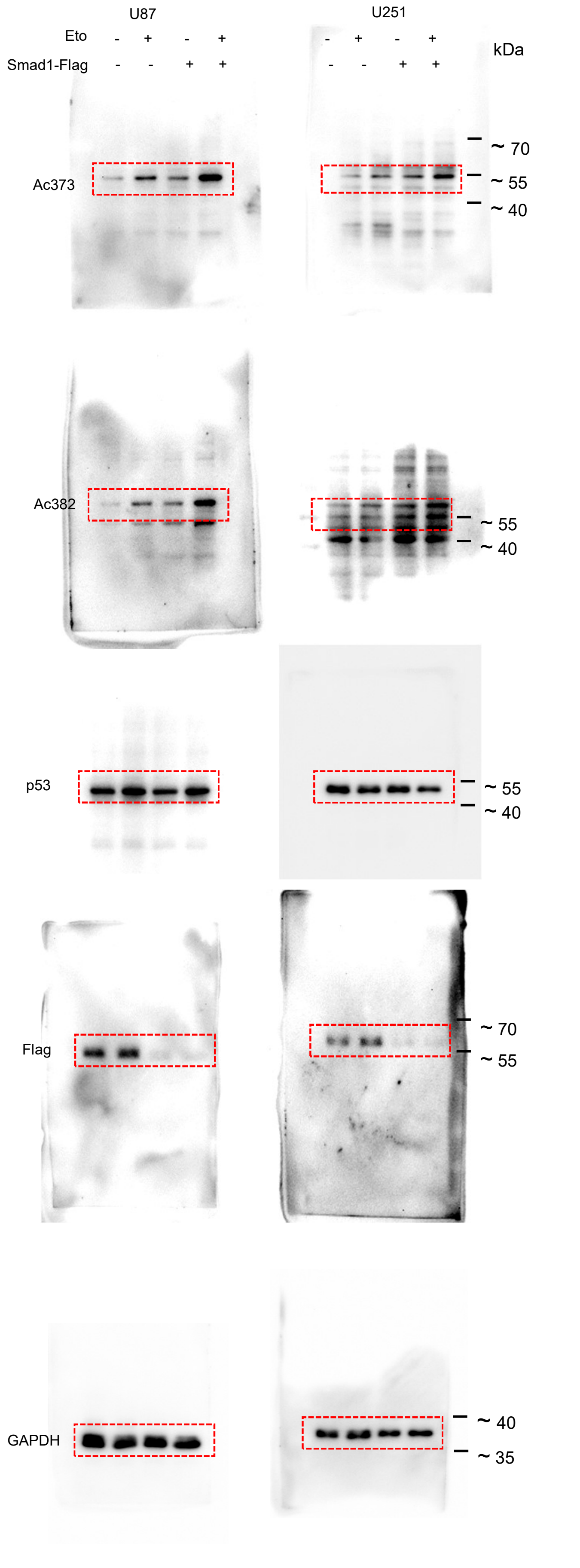

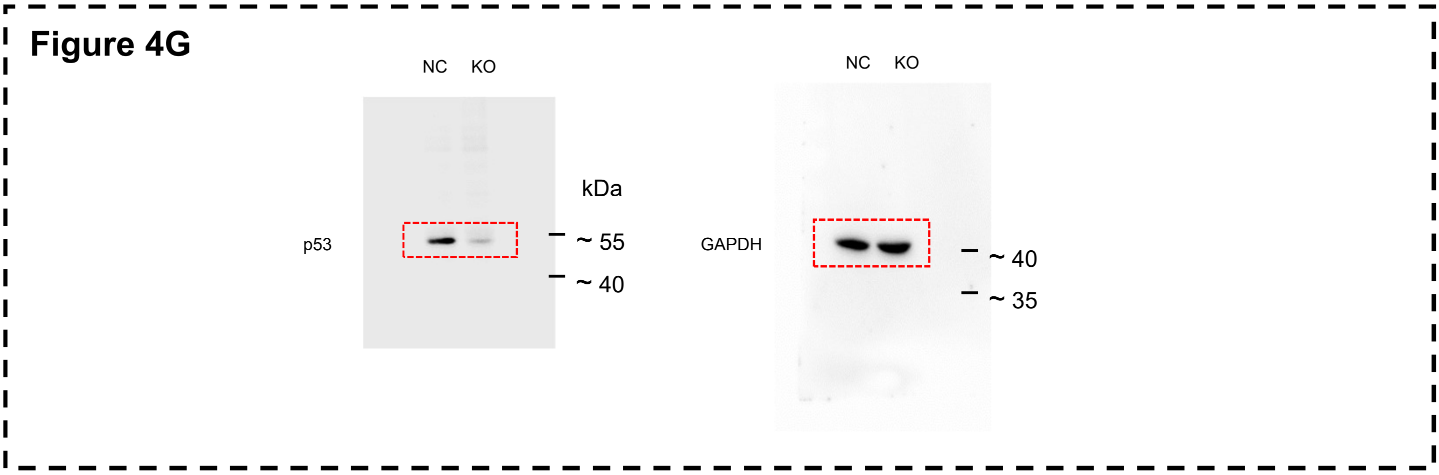
Figure 4G
NC KO
p53
kDa
~ 55
~ 40
NC KO
GAPDH
~ 40
~ 35

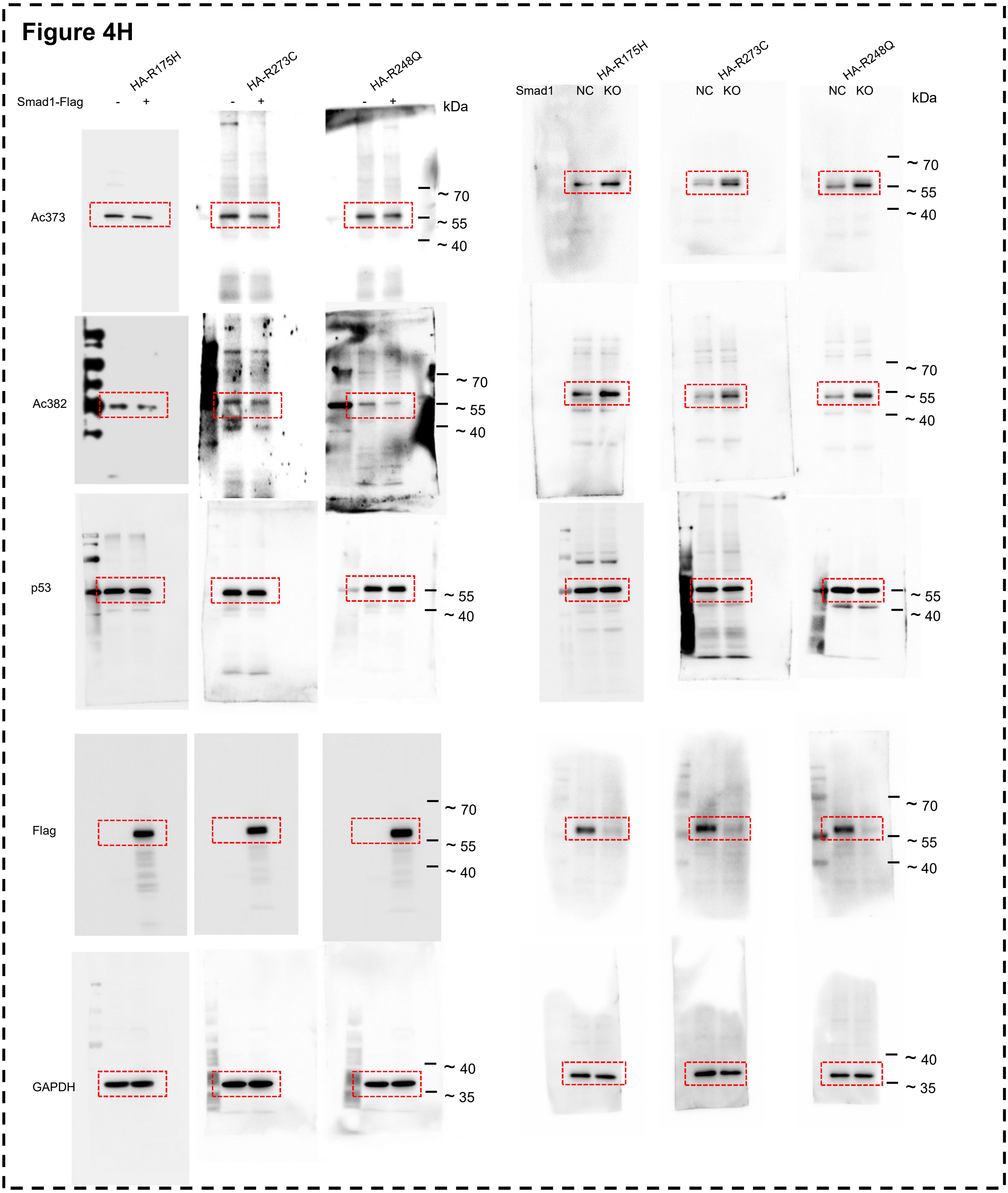
Figure 4H
HA-R175H
HA-R273C
HA-R248Q
Smad1-Flag - +
- +
- +
kDa
Ac373
Ac382
p53
Flag
GAPDH
~ 70
~ 55
~ 40
~ 35
Smad1 NC KO
NC KO
NC KO

Figure 4I

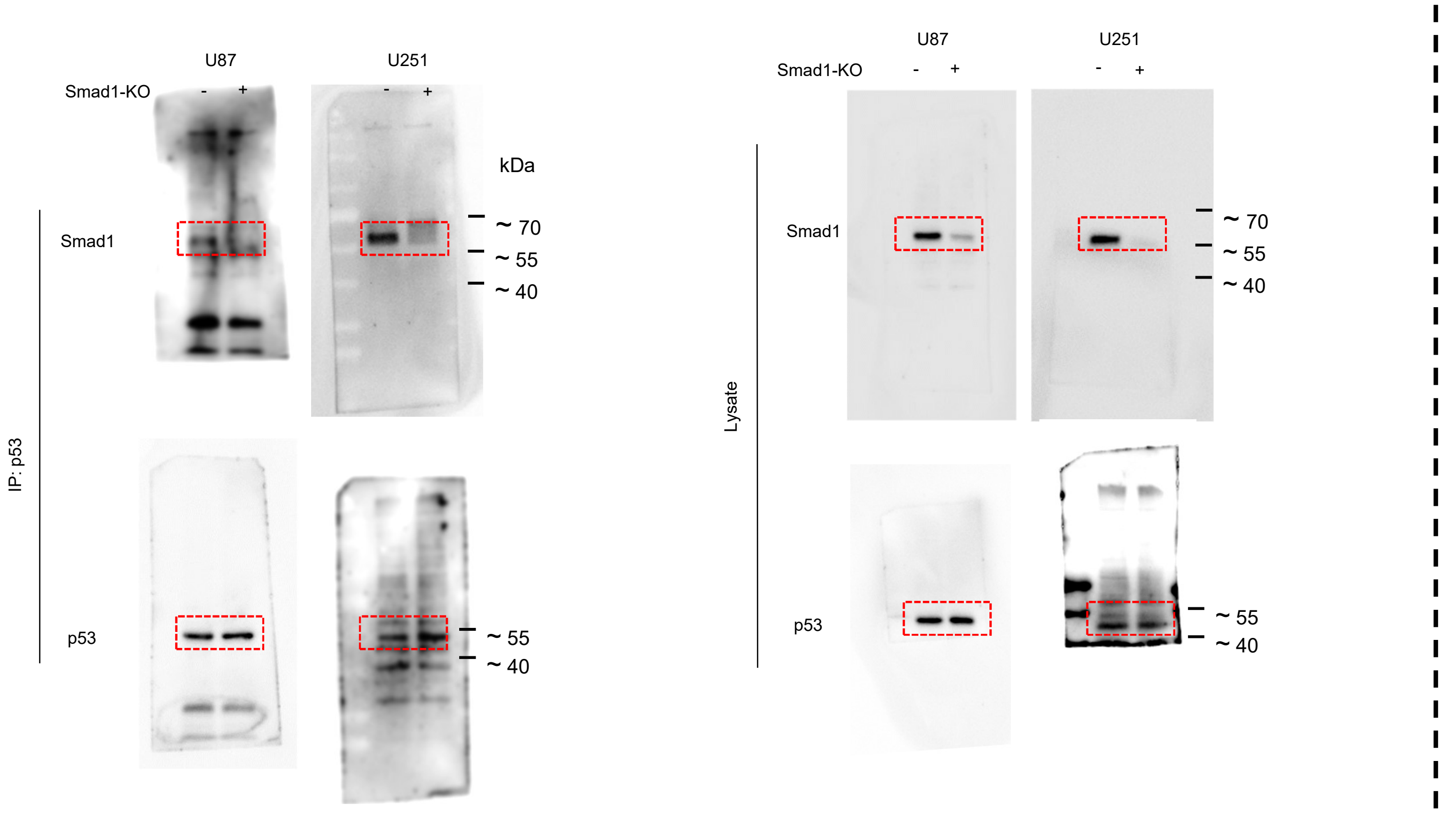

Figure 4L

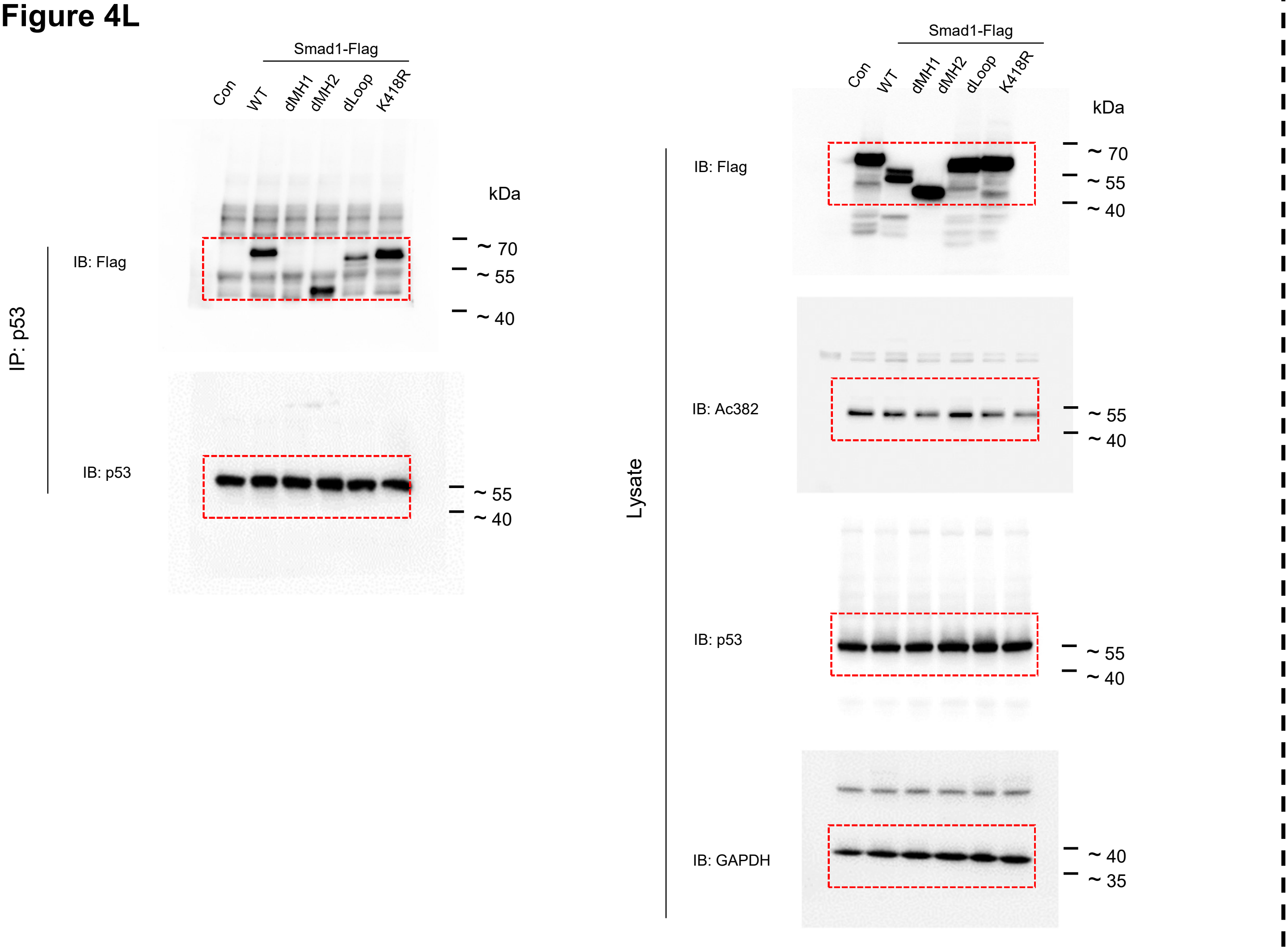

Figure 4M

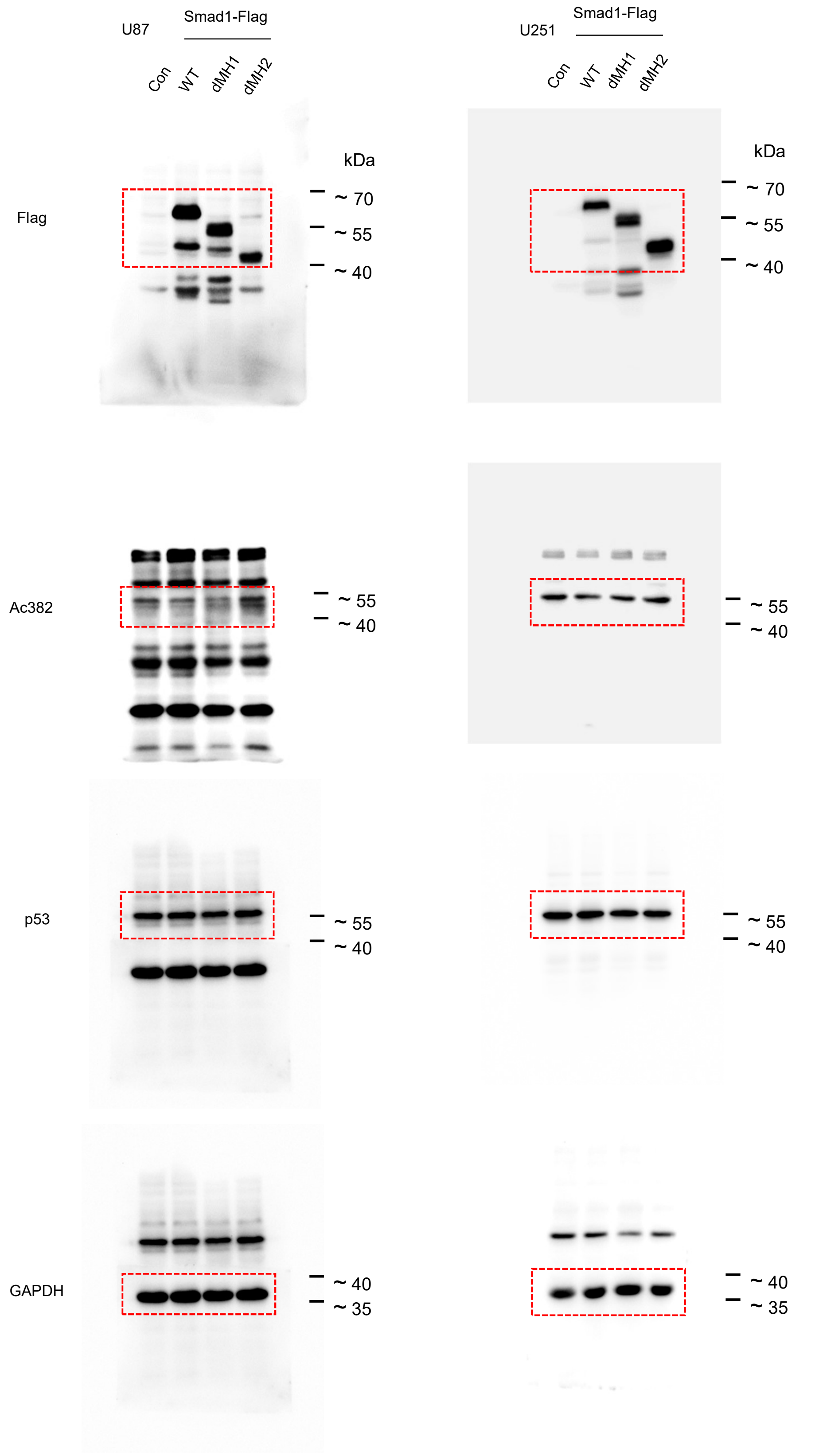
U87
Smad1-Flag
Con
WT
dMH1
dMH2
U251
Smad1-Flag
Con
WT
dMH1
dMH2
kDa
Flag
~ 70
~ 55
~ 40
kDa
~ 70
~ 55
~ 40
Ac382
~ 55
~ 40
~ 55
~ 40
p53
~ 55
~ 40
~ 55
~ 40
GAPDH
~ 40
~ 35
~ 40
~ 35

Figure 5A

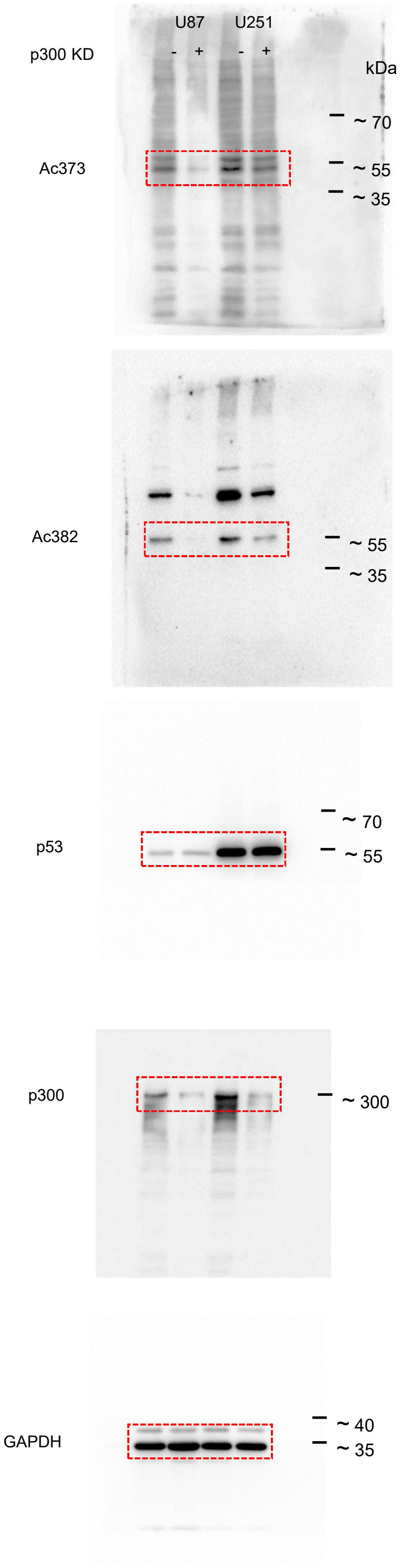

Figure 5B

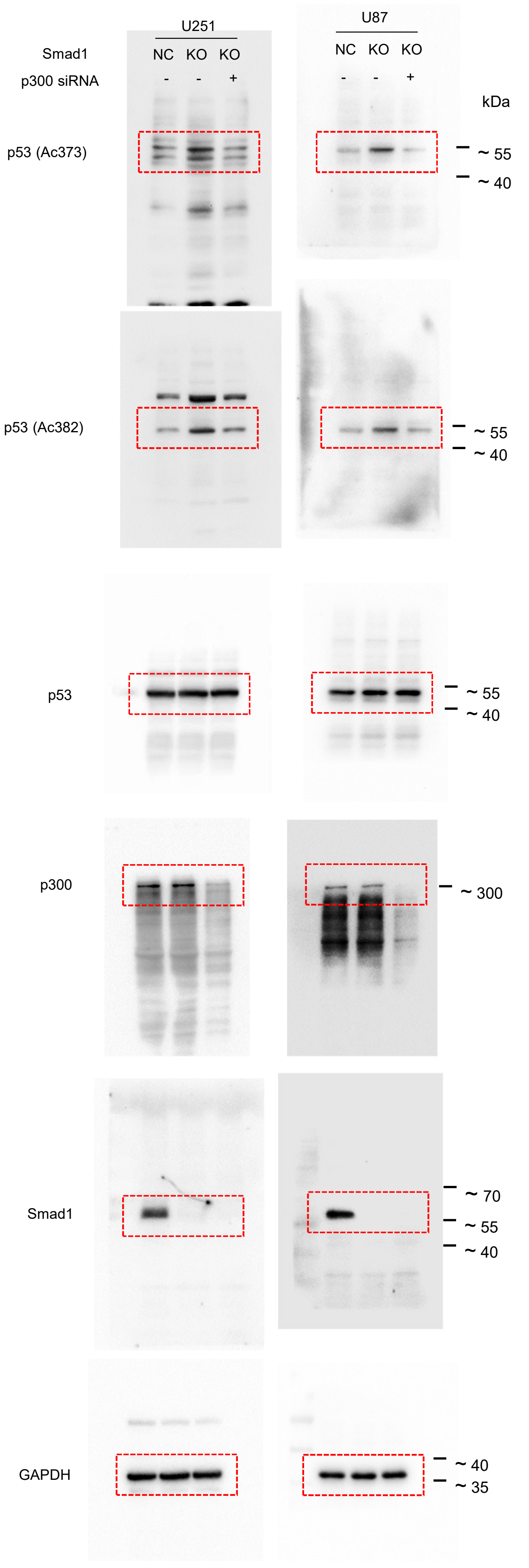

Figure 5C

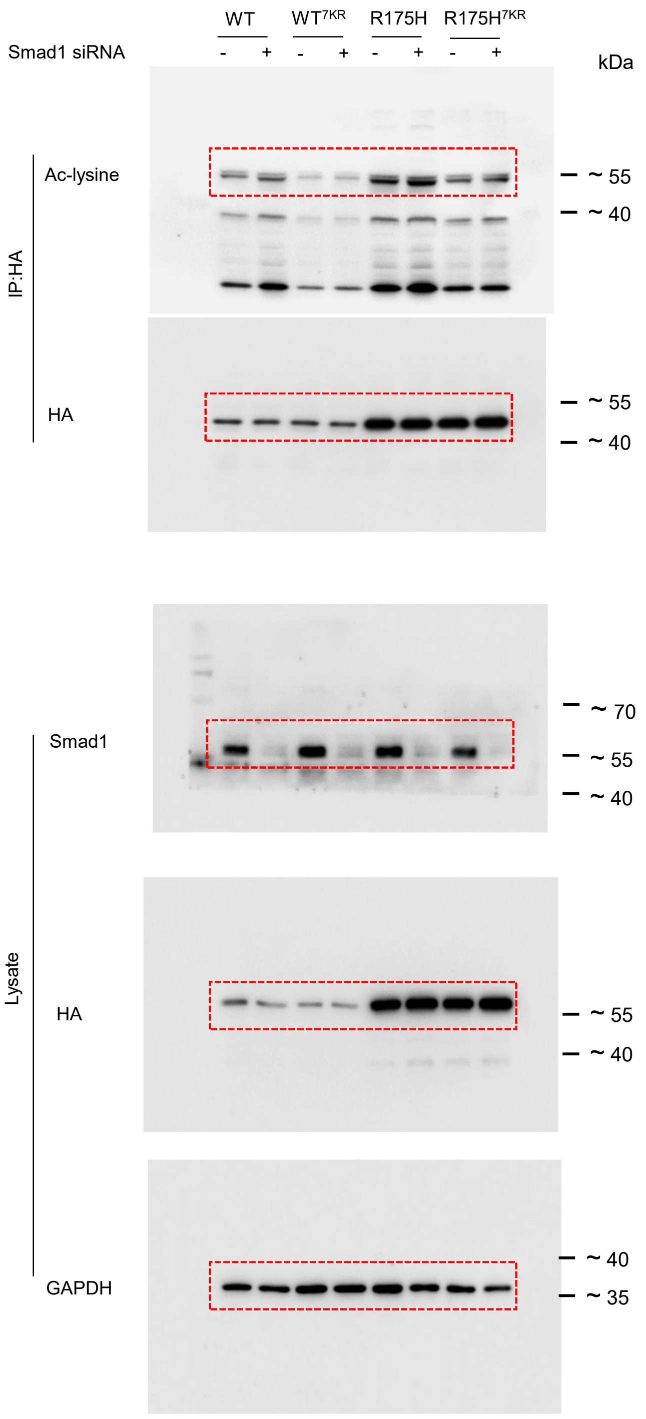

Figure 5D

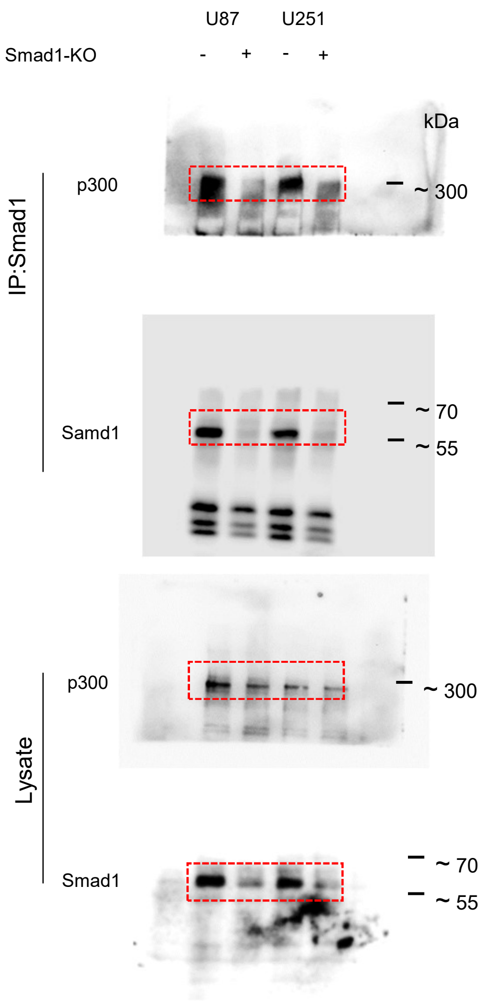

Figure 5F

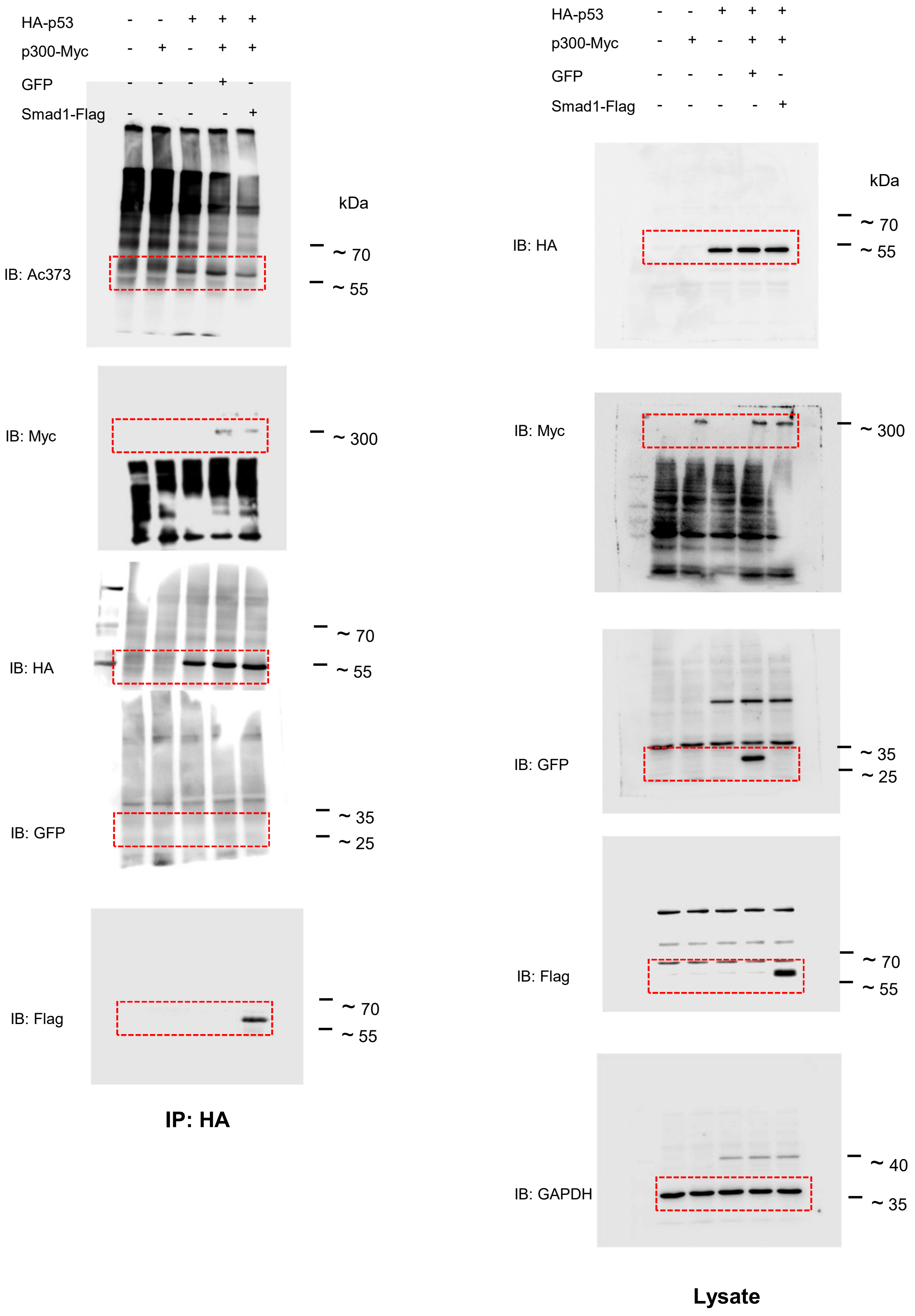

HA-p53 - - + + +
p300-Myc - + - + +
GFP - - - + -
Smad1-Flag - - - - +
IB: Ac373
kDa
~ 70
~ 55
IB: Myc
~ 300
IB: HA
~ 70
~ 55
IB: GFP
~ 35
~ 25
IB: Flag
~ 70
~ 55
IP: HA
HA-p53 - - + + +
p300-Myc - + - + +
GFP - - - + -
Smad1-Flag - - - - +
IB: HA
kDa
~ 70
~ 55
IB: Myc
~ 300
IB: GFP
~ 35
~ 25
IB: Flag
~ 70
~ 55
IB: GAPDH
~ 40
~ 35
Lysate

Figure 5G

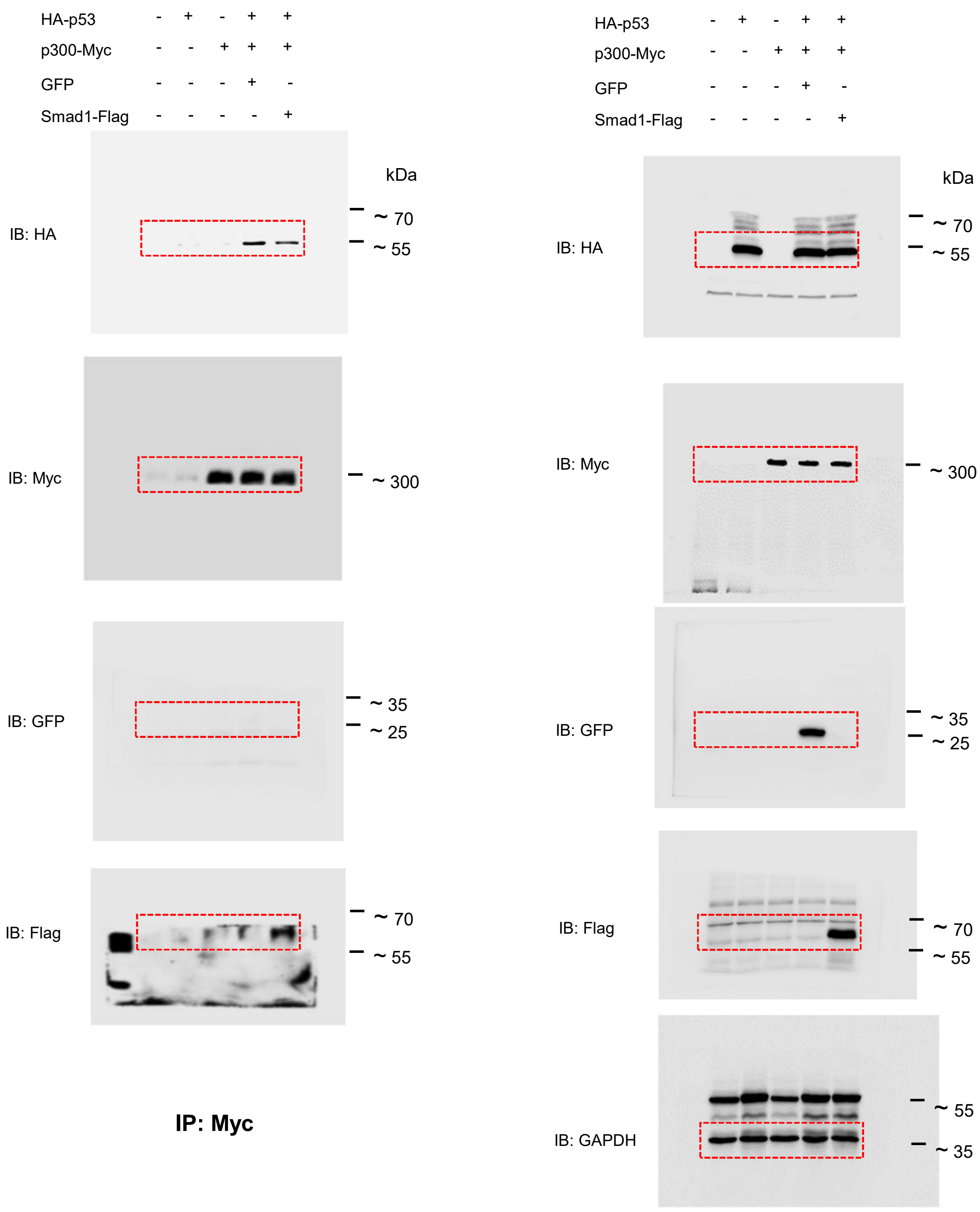

IP: Myc

Lysate

Figure 5H

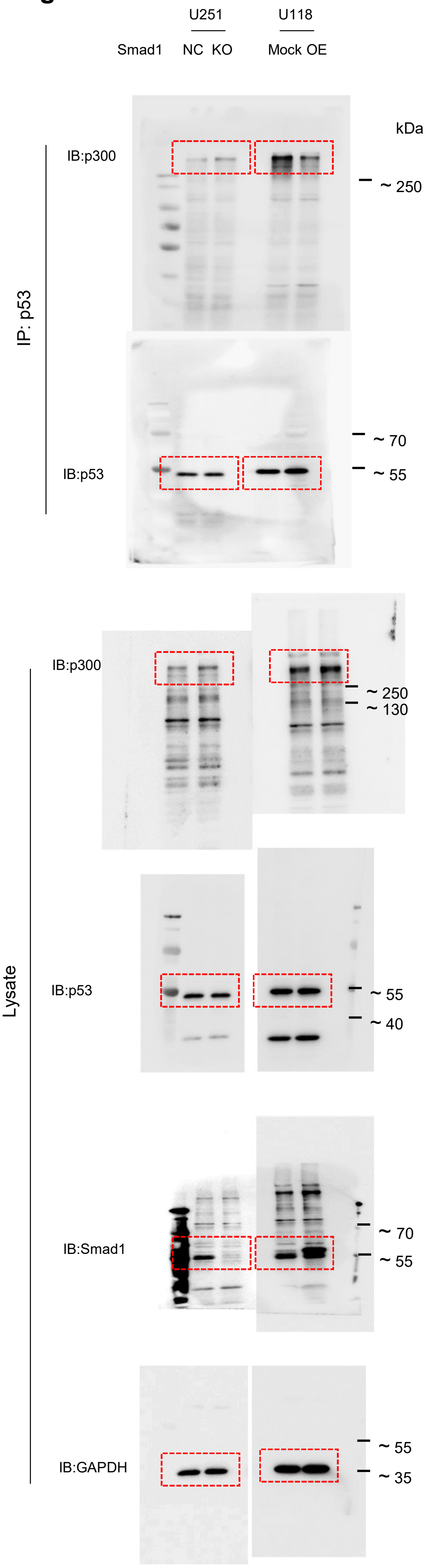

Figure 5I

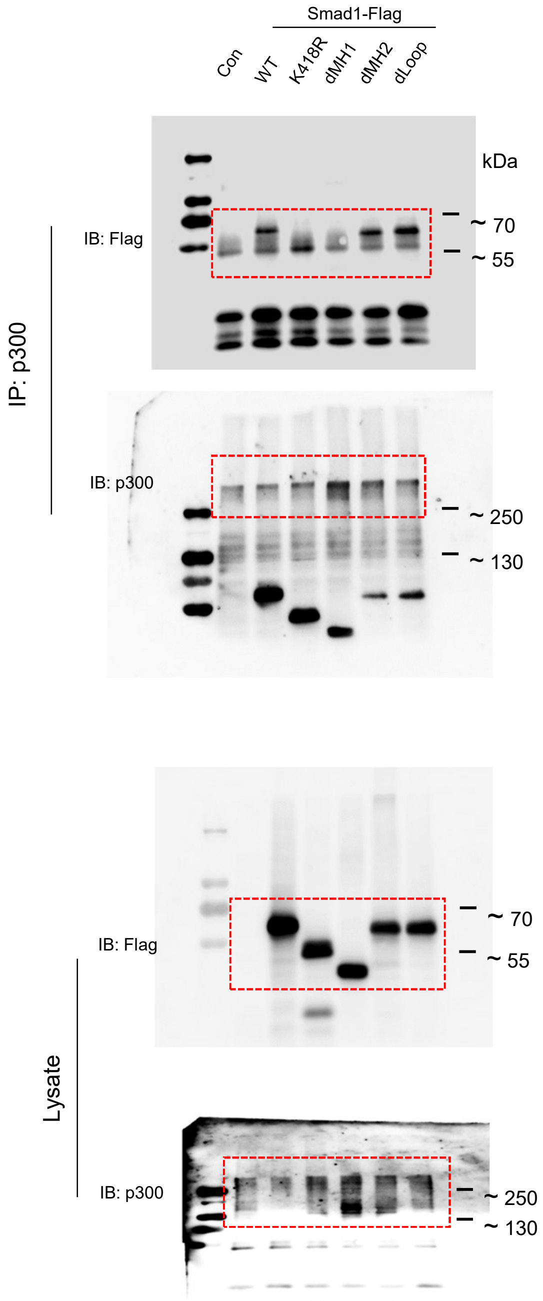

Figure 5J

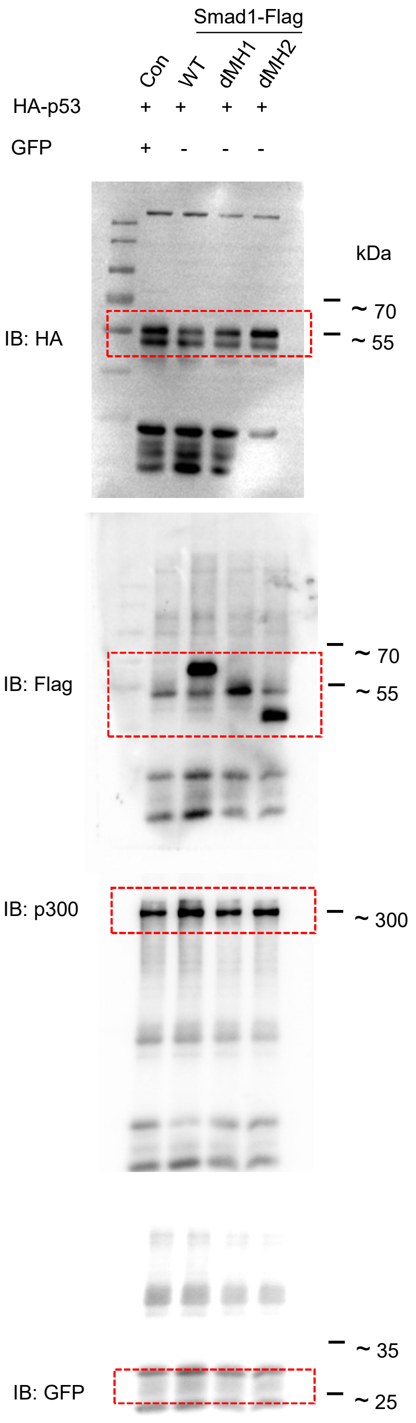

IP: p300

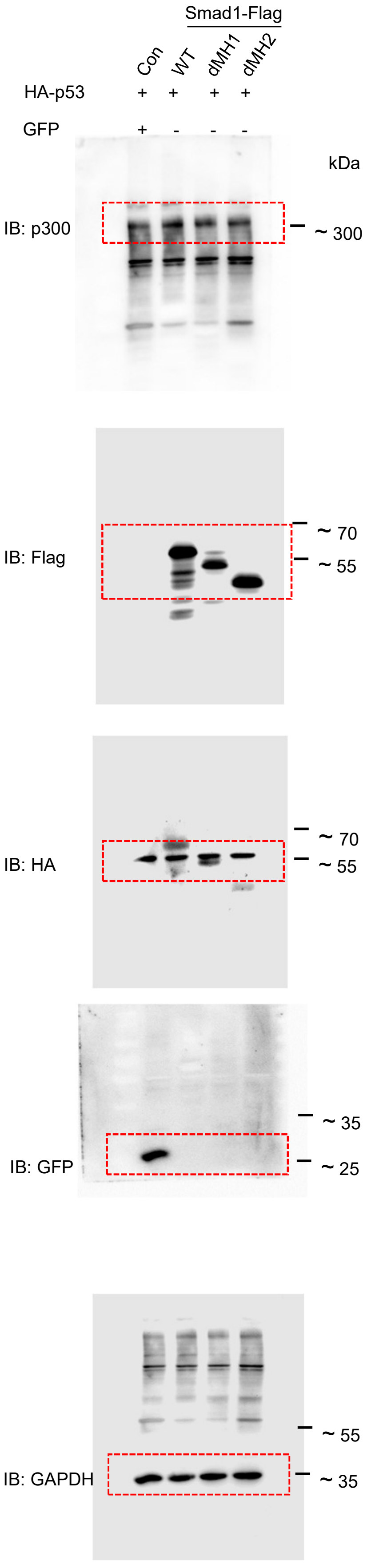

Lysate

Figure 5L

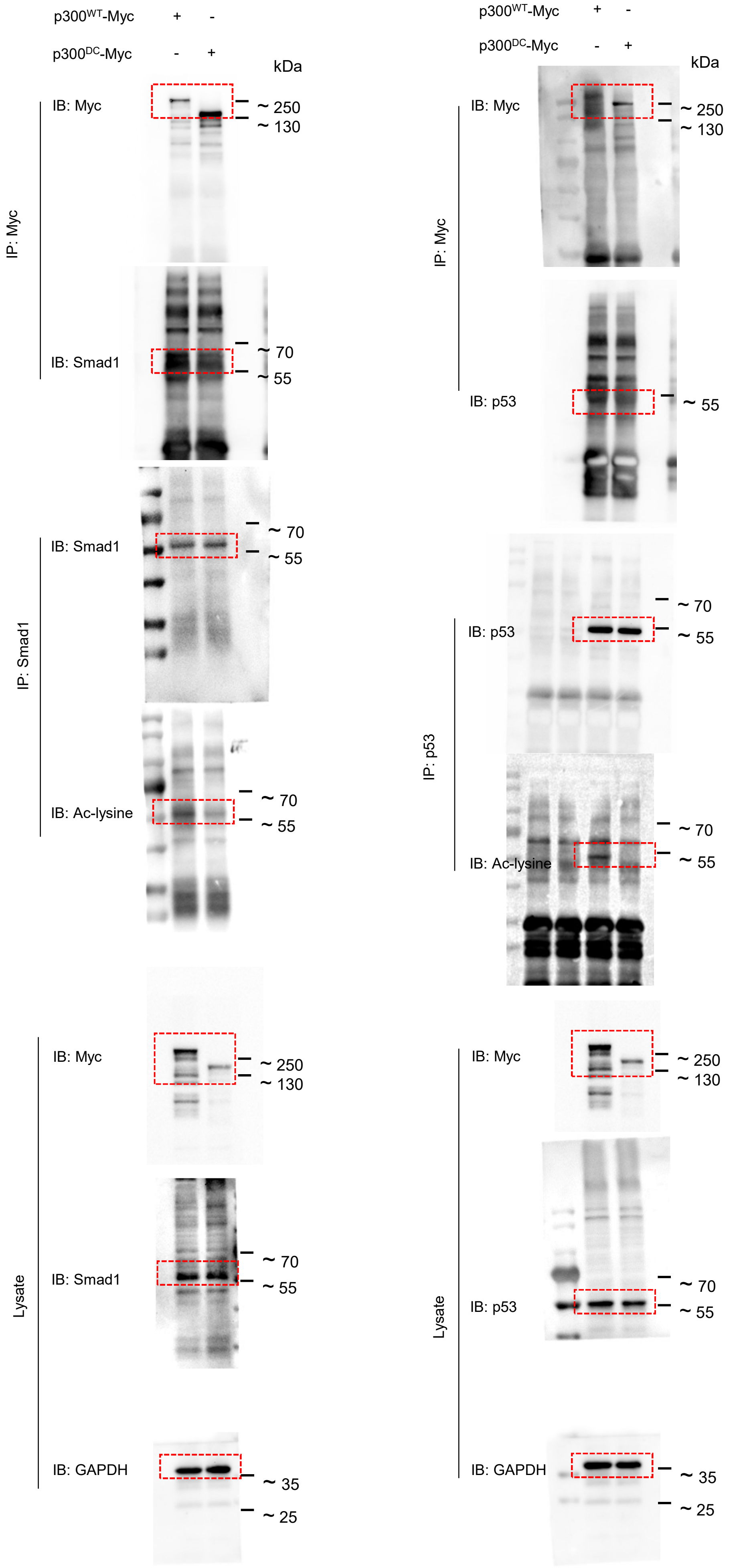

p300WT-Myc + -
p300DC-Myc - +
kDa
IP: Myc
IB: Myc
~ 250
~ 130
IB: Smad1
~ 70
~ 55
IP: Smad1
IB: Smad1
~ 70
~ 55
IB: Ac-lysine
~ 70
~ 55
Lysate
IB: Myc
~ 250
~ 130
IB: Smad1
~ 70
~ 55
IB: GAPDH
~ 35
~ 25
p300WT-Myc + -
p300DC-Myc - +
kDa
IP: Myc
IB: Myc
~ 250
~ 130
IB: p53
~ 55
IP: p53
IB: p53
~ 70
~ 55
IB: Ac-lysine
~ 70
~ 55
Lysate
IB: Myc
~ 250
~ 130
IB: p53
~ 70
~ 55
IB: GAPDH
~ 35
~ 25

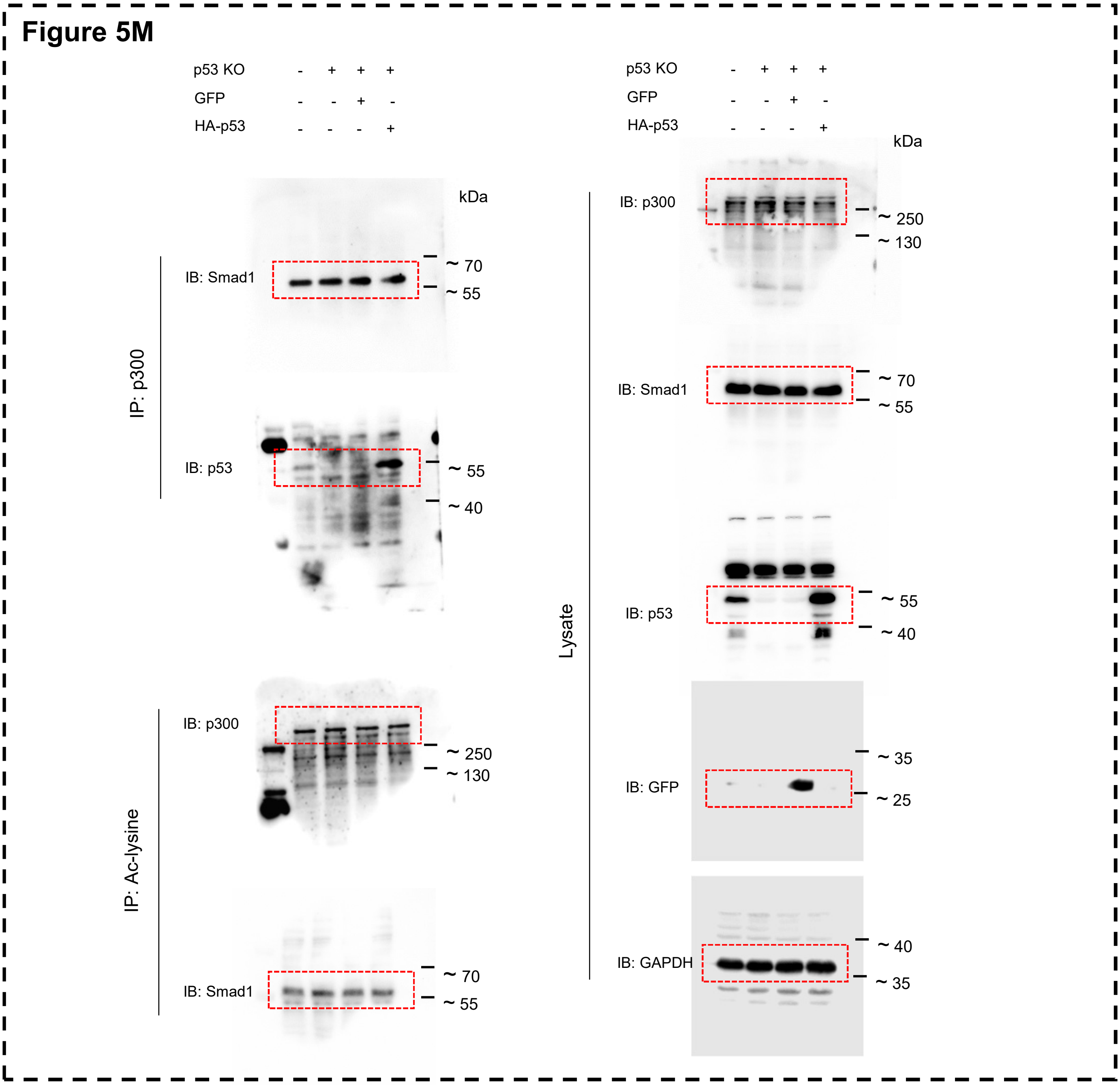
Figure 5M
p53 KO - + + +
GFP - - + -
HA-p53 - - - +
kDa
IP: p300
IB: Smad1
~ 70
~ 55
IB: p53
~ 55
~ 40
IP: Ac-lysine
IB: p300
~ 250
~ 130
IB: Smad1
~ 70
~ 55
Lysate
IB: p300
~ 250
~ 130
IB: Smad1
~ 70
~ 55
IB: p53
~ 55
~ 40
IB: GFP
~ 35
~ 25
IB: GAPDH
~ 40
~ 35

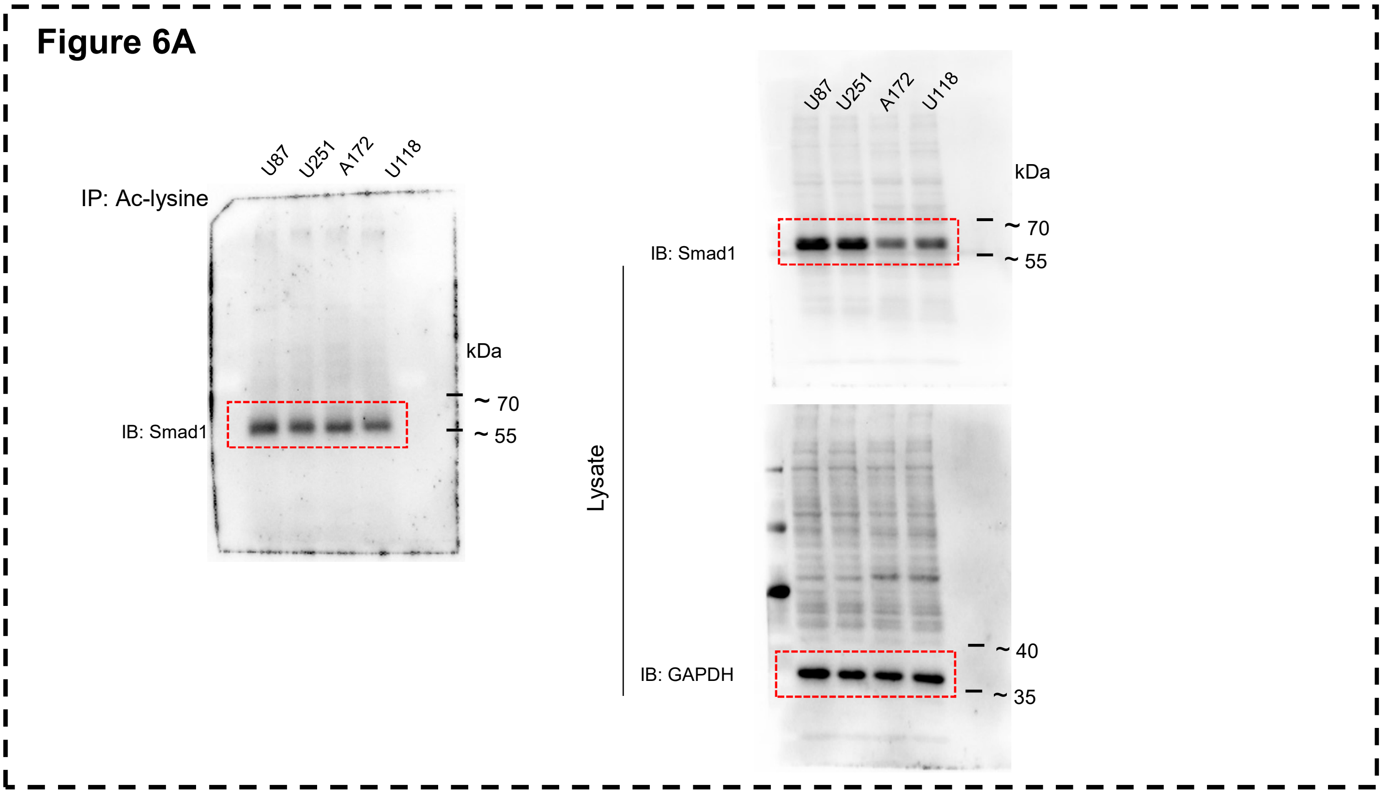
Figure 6A
IP: Ac-lysine
U87
U251
A172
U118
kDa
IB: Smad1
~ 70
~ 55
Lysate
IB: Smad1
~ 70
~ 55
IB: GAPDH
~ 40
~ 35

Figure 6B

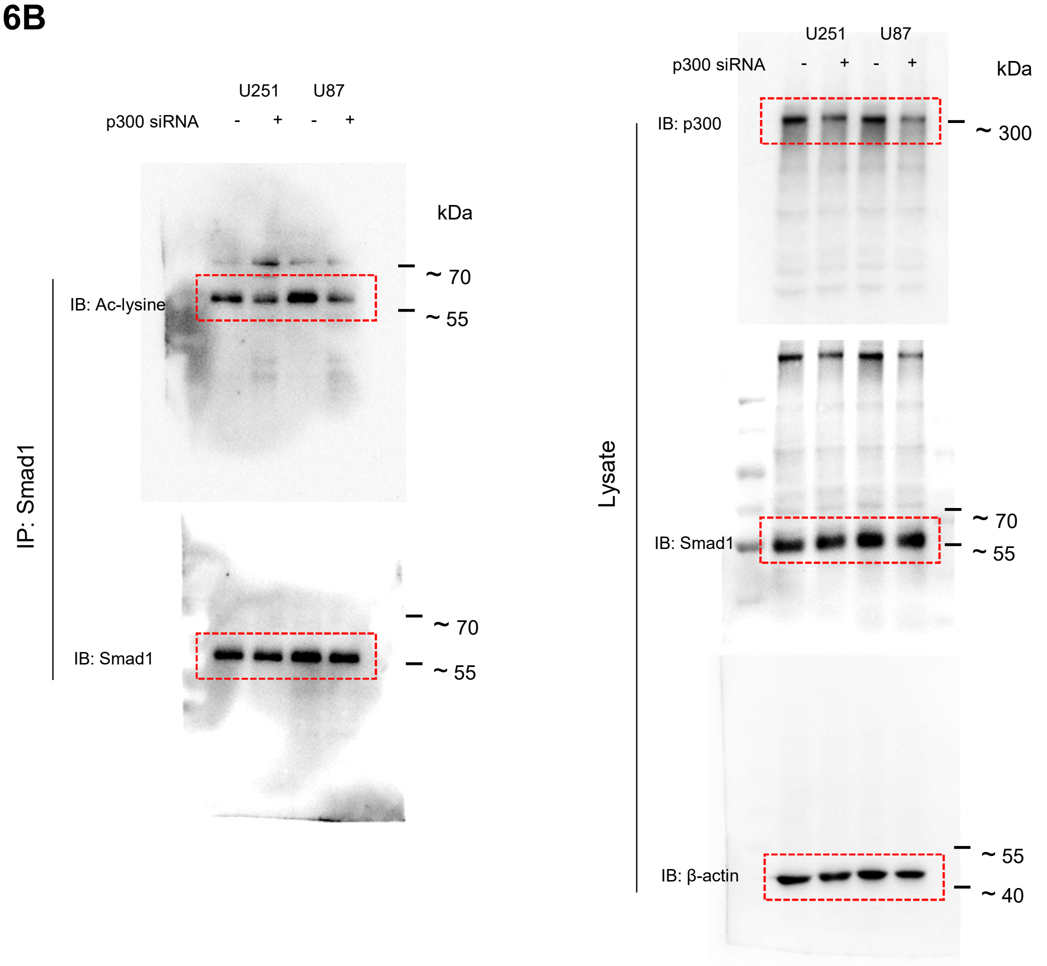

Figure 6D

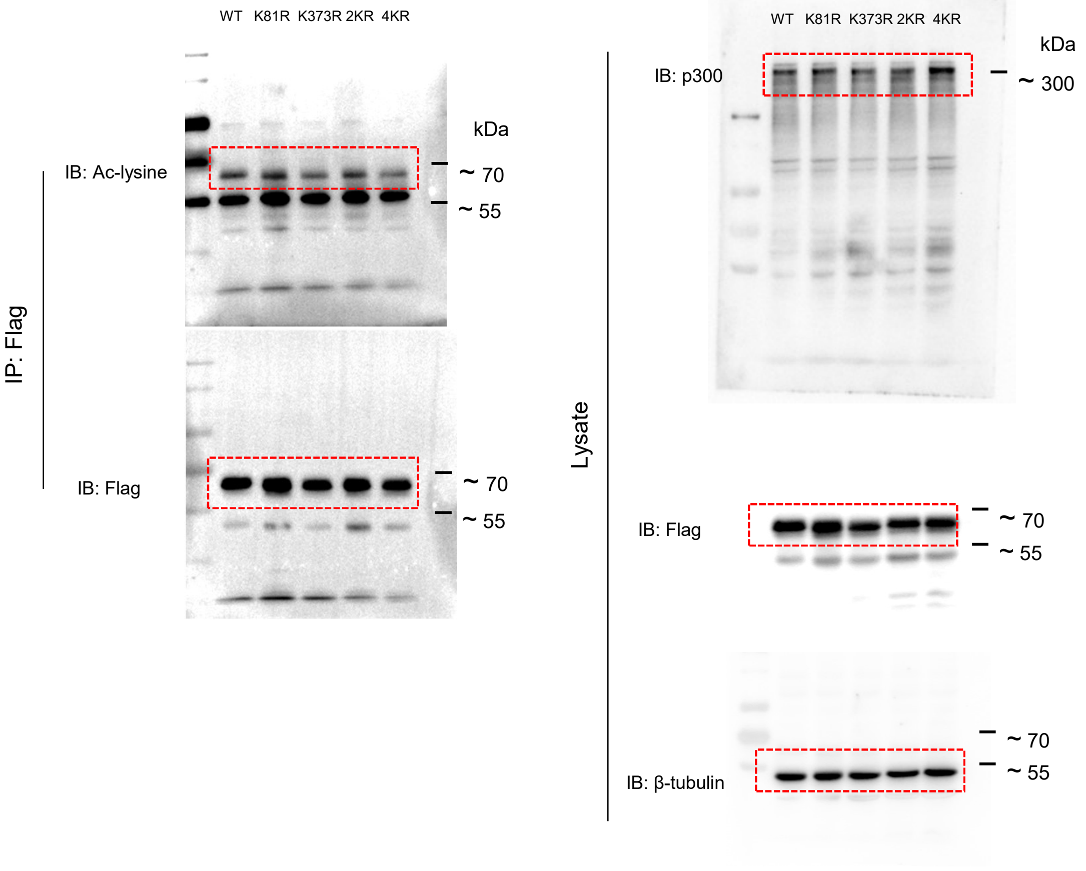

Figure 6F

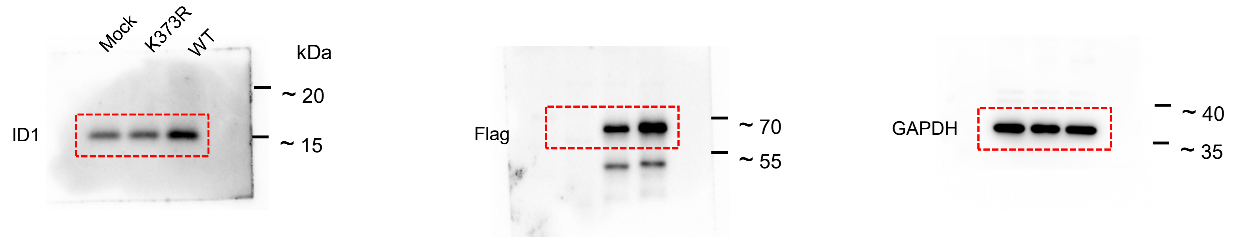

Figure 6K

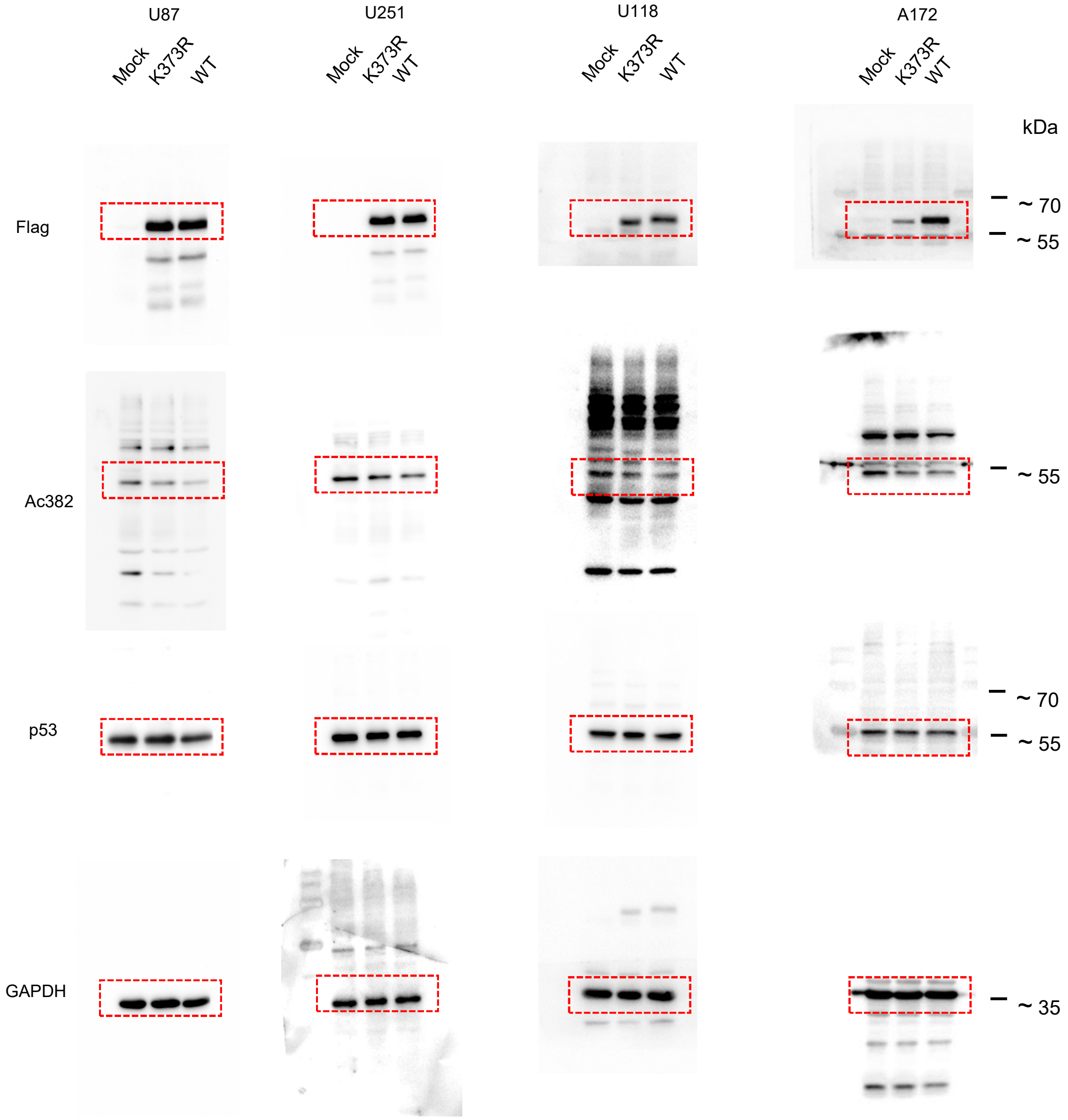

Figure 7J

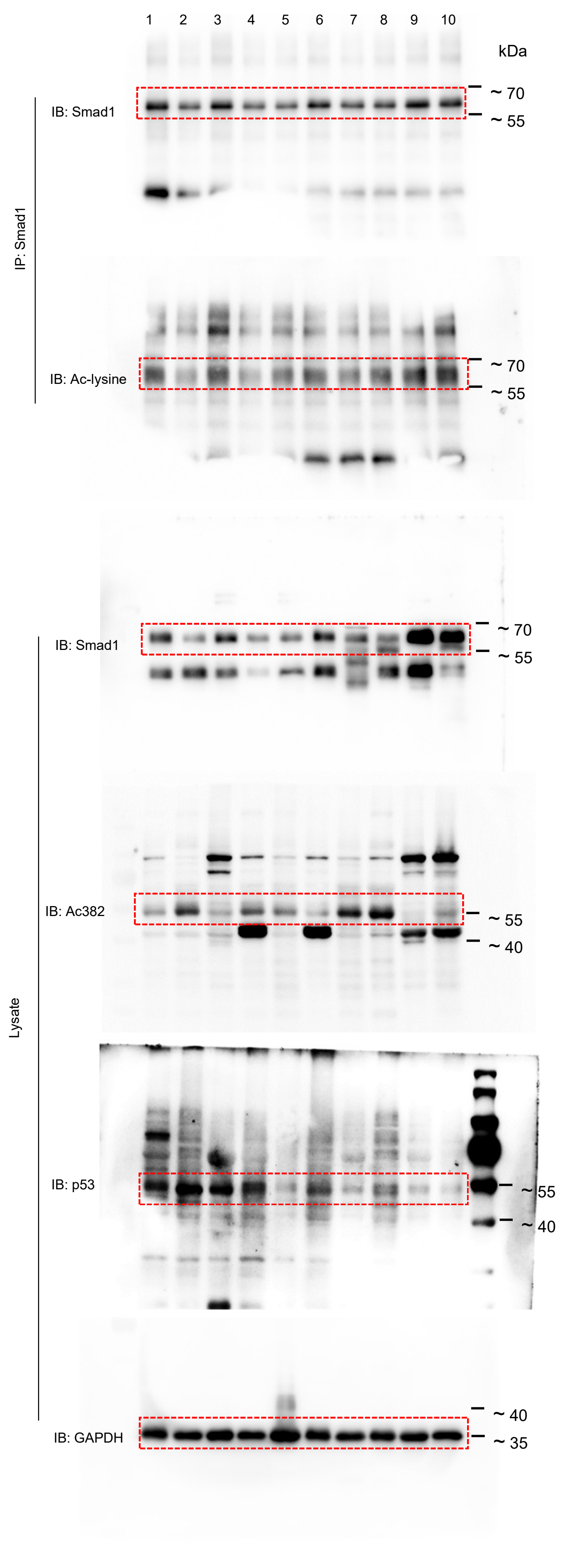

Figure 8K

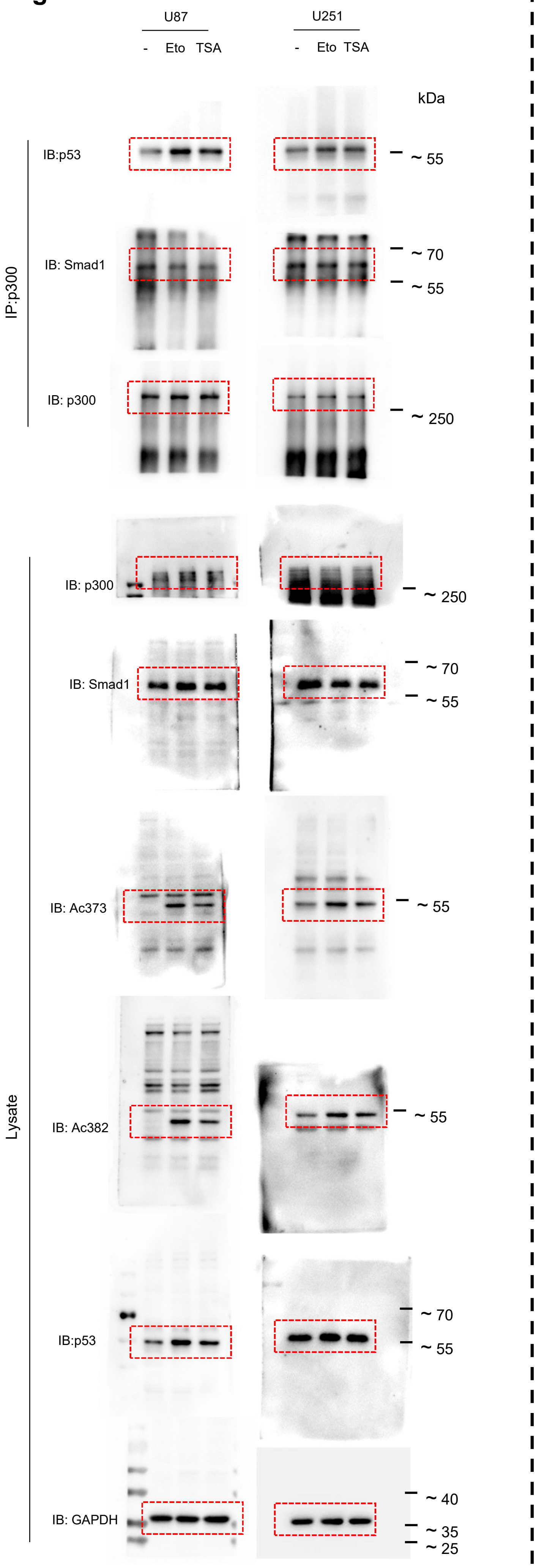

Figure 8L

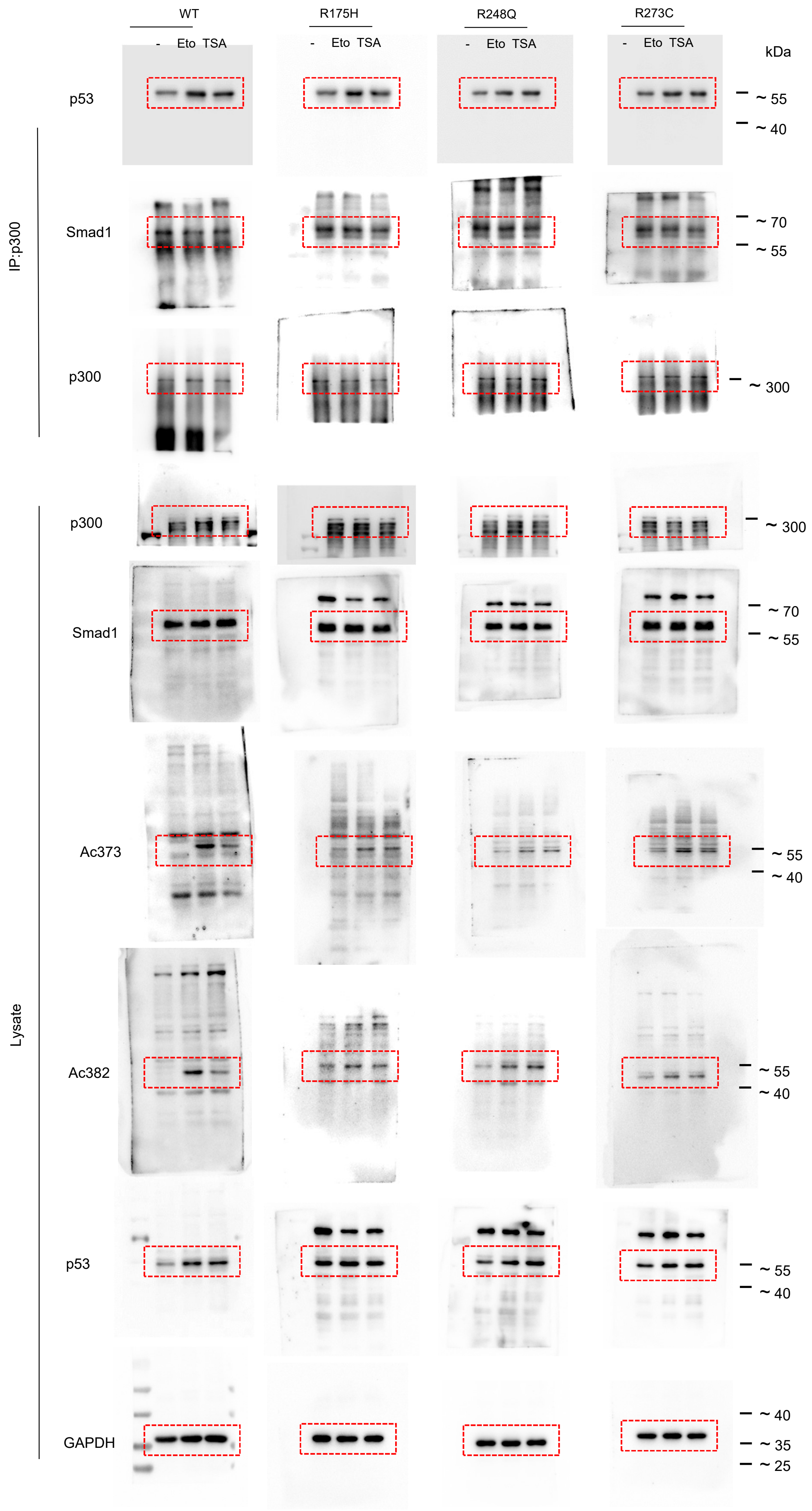

WT
R175H
R248Q
R273C
- Eto TSA
- Eto TSA
- Eto TSA
- Eto TSA
kDa
p53
~ 55
~ 40
IP:p300
Smad1
~ 70
~ 55
p300
~ 300
Lysate
p300
~ 300
Smad1
~ 70
~ 55
Ac373
~ 55
~ 40
Ac382
~ 55
~ 40
p53
~ 55
~ 40
GAPDH
~ 40
~ 35
~ 25

Figure 9D

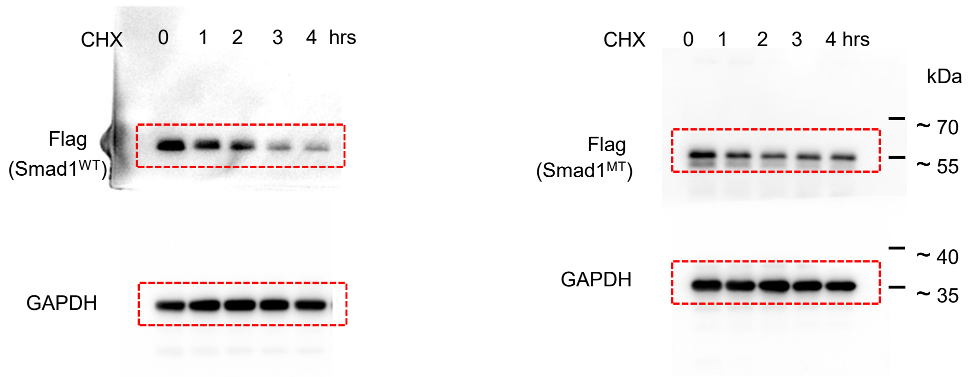

Figure 9E

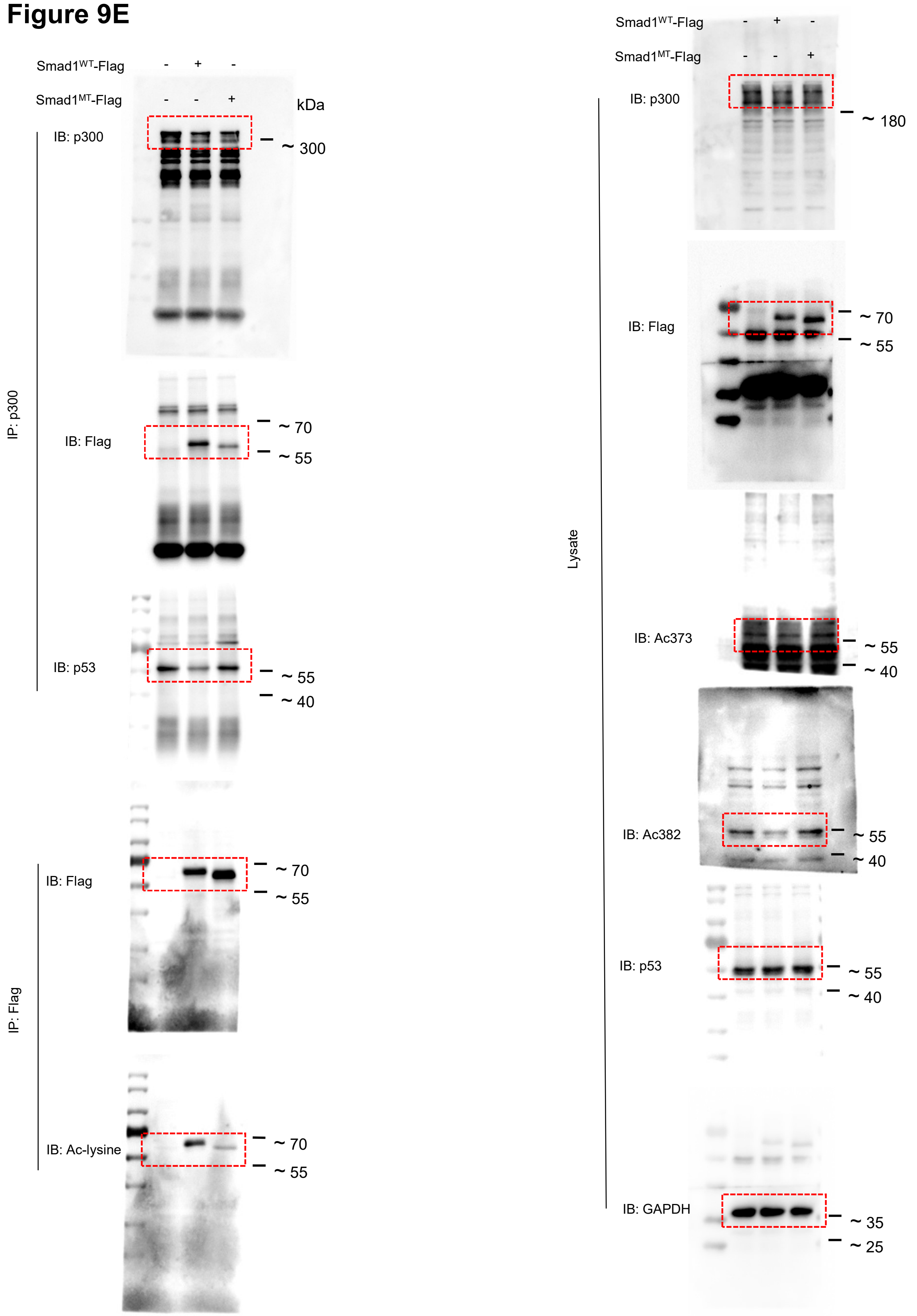

Figure 9H

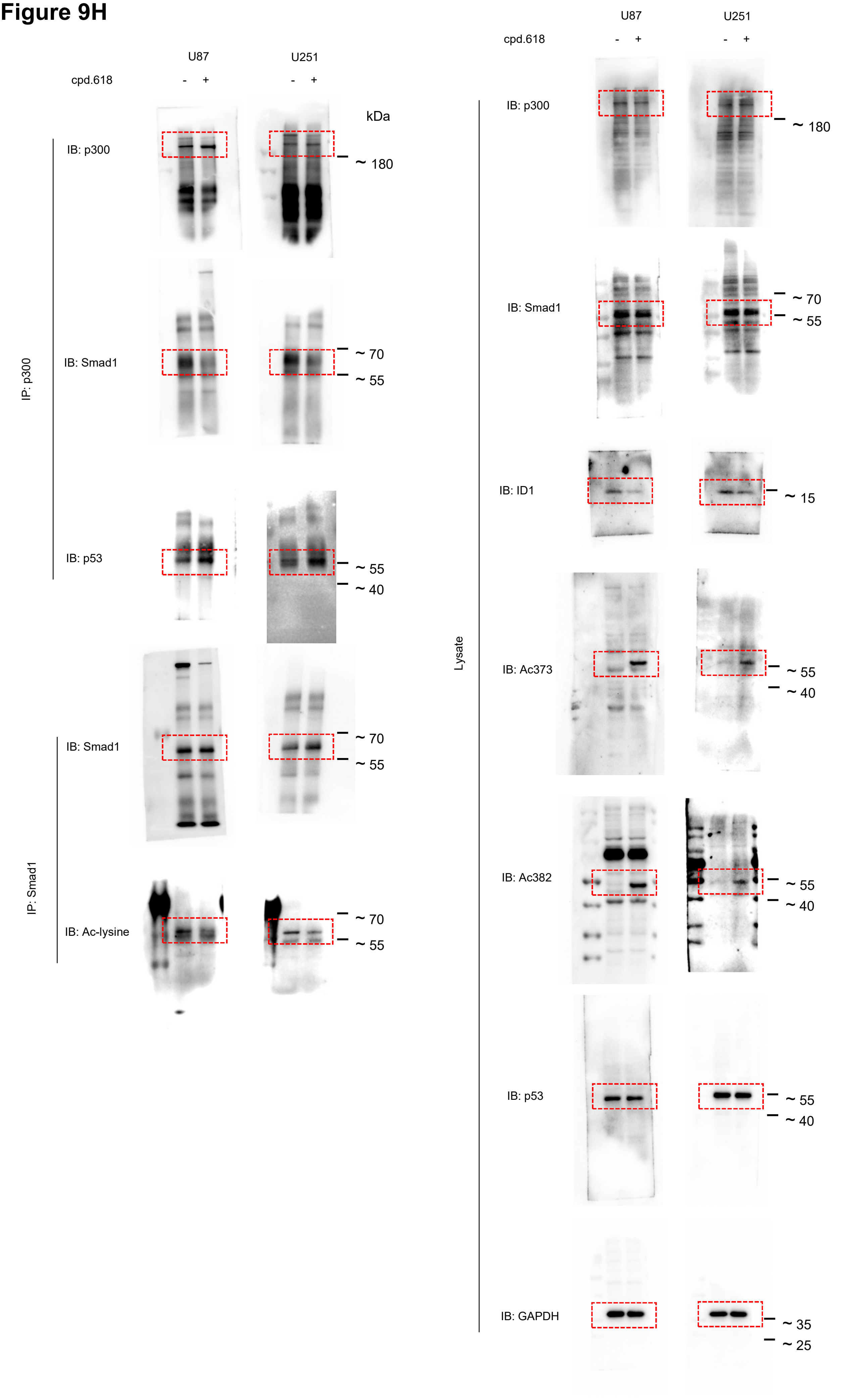

U87
U251
cpd.618
- +
- +
kDa
IP: p300
IB: p300
~ 180
IB: Smad1
~ 70
~ 55
IB: p53
~ 55
~ 40
IP: Smad1
IB: Smad1
~ 70
~ 55
IB: Ac-lysine
~ 70
~ 55
Lysate
IB: p300
~ 180
IB: Smad1
~ 70
~ 55
IB: ID1
~ 15
IB: Ac373
~ 55
~ 40
IB: Ac382
~ 55
~ 40
IB: p53
~ 55
~ 40
IB: GAPDH
~ 35
~ 25

Figure 9L

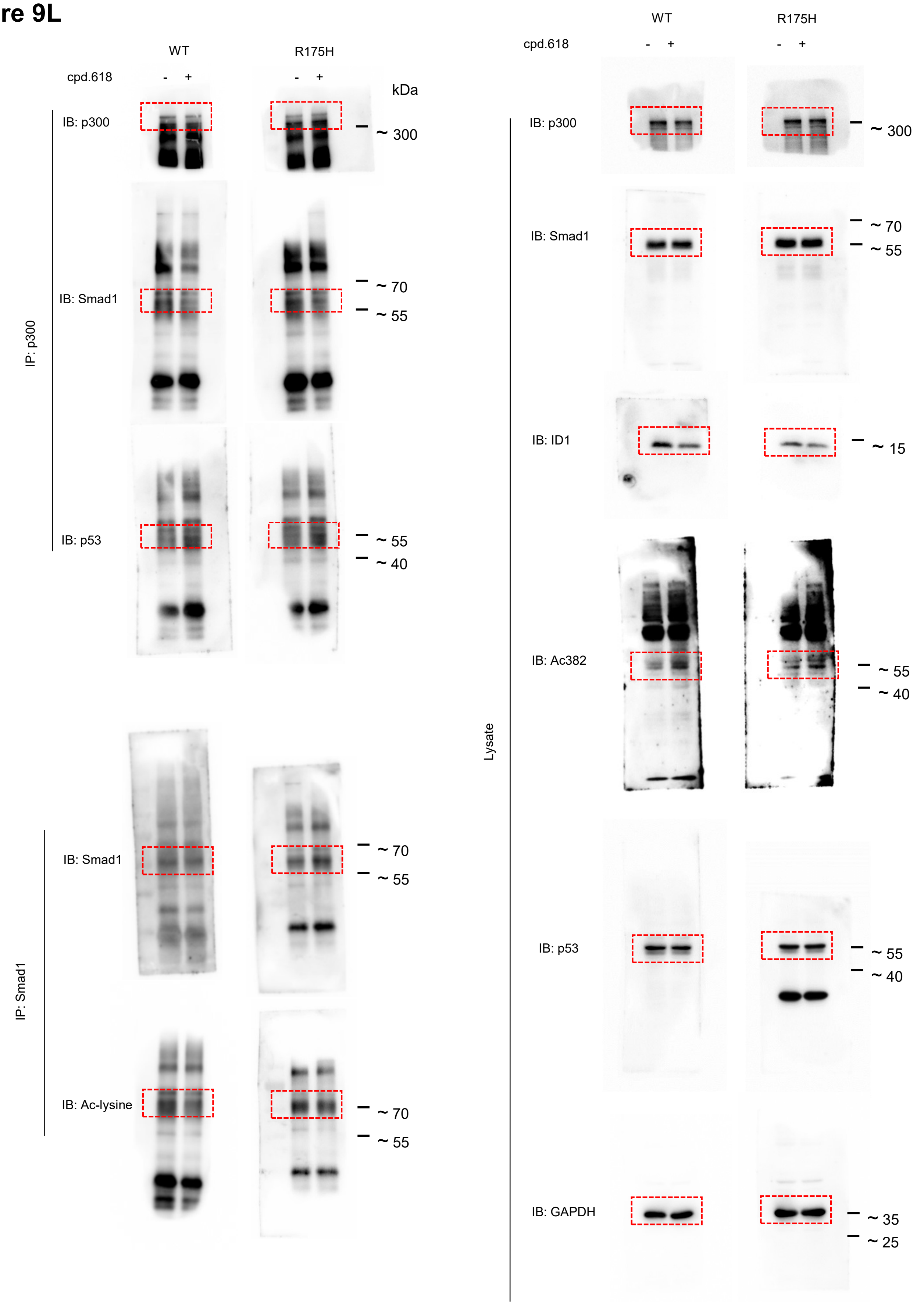

WT
R175H
cpd.618
- +
- +
kDa
IB: p300
~ 300
IB: Smad1
~ 70
~ 55
IB: p53
~ 55
~ 40
IP: p300
IB: Smad1
~ 70
~ 55
IB: Ac-lysine
~ 70
~ 55
IP: Smad1
WT
R175H
cpd.618
- +
- +
IB: p300
~ 300
IB: Smad1
~ 70
~ 55
IB: ID1
~ 15
IB: Ac382
~ 55
~ 40
IB: p53
~ 55
~ 40
IB: GAPDH
~ 35
~ 25
Lysate

Supplementary Fig.2A

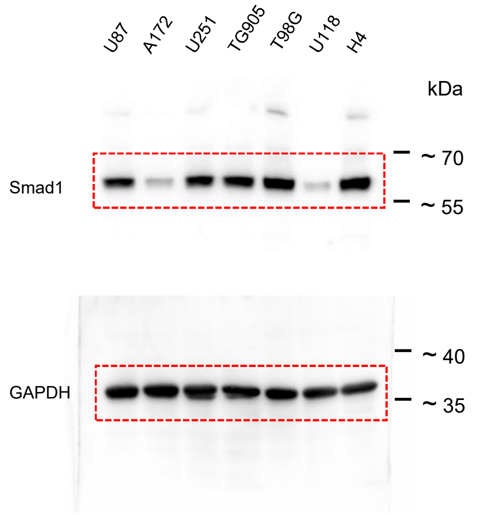

Supplementary Fig.2C

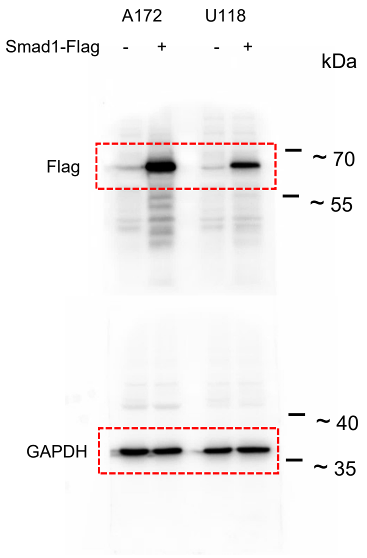

Supplementary Fig.2B

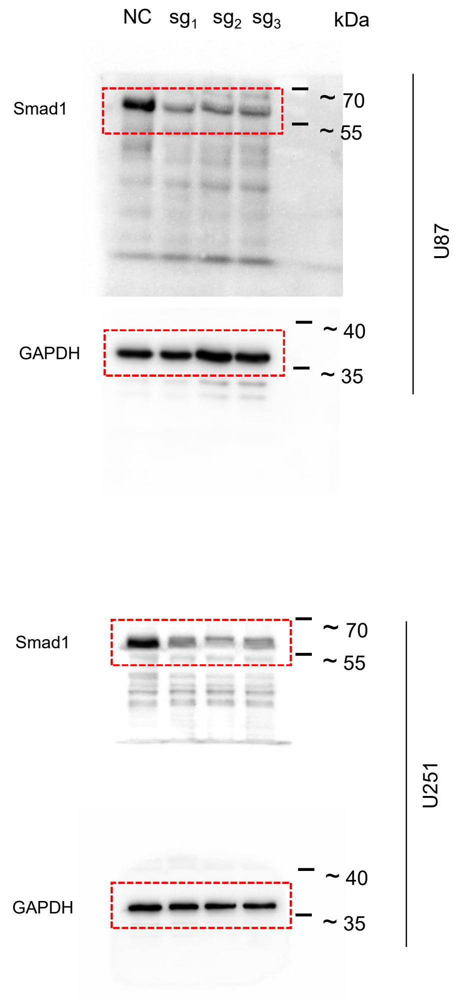

Supplementary Fig.3A

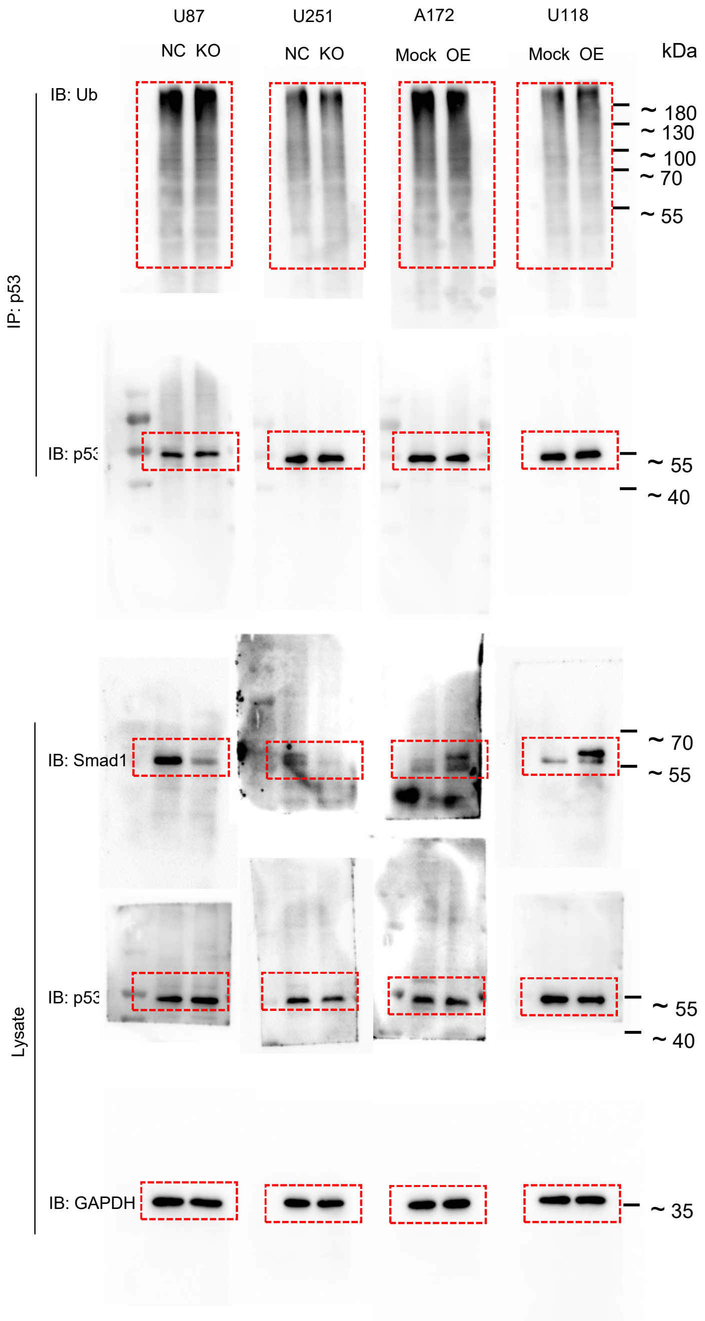

Supplementary Fig.3B

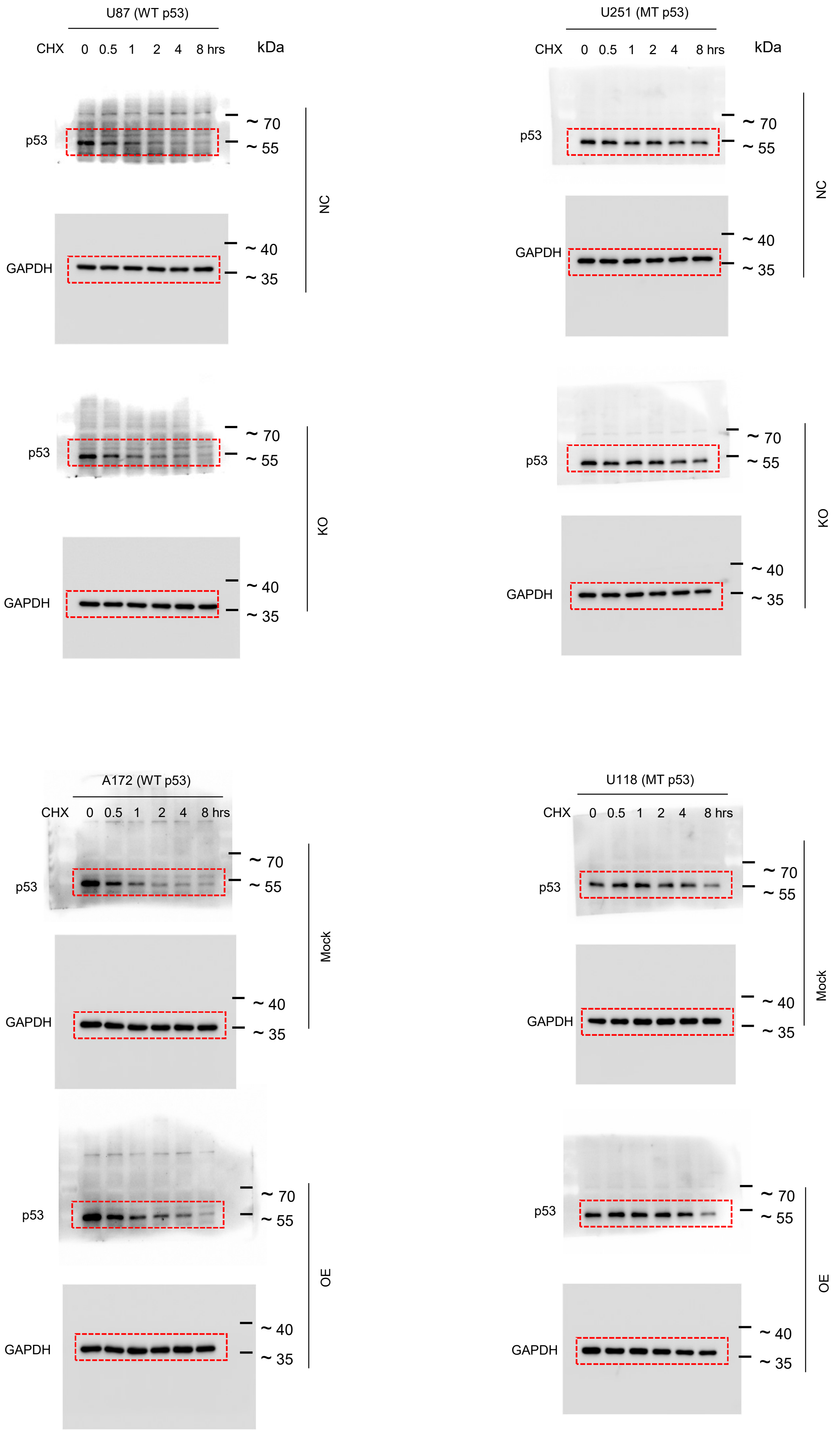

Supplementary Fig.4

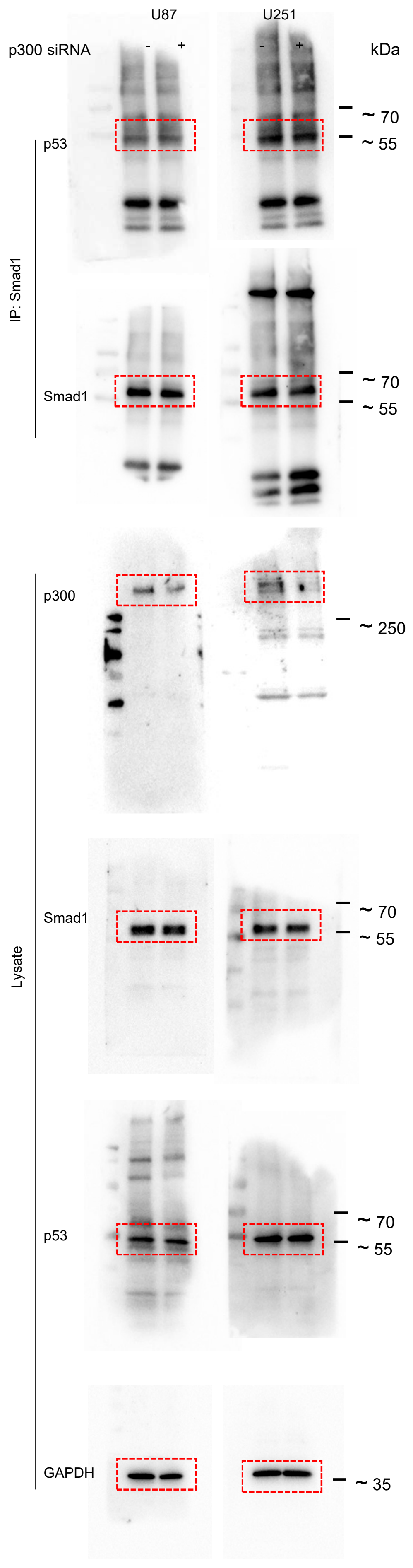

Supplementary Fig.9

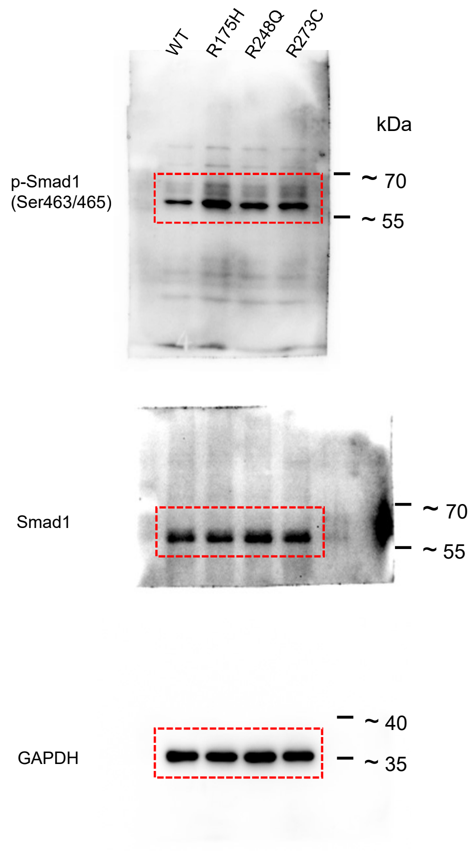

Supplement: Supplementary file 3 — Supporting Information [file ADVS-12-2402258-s001.pdf]
